# Transcriptomic Profiling of Apple Calli With a Focus on the Key Genes for ALA-Induced Anthocyanin Accumulation

**DOI:** 10.3389/fpls.2021.640606

**Published:** 2021-03-26

**Authors:** Jie Zheng, Longbo Liu, Huihui Tao, Yuyan An, Liangju Wang

**Affiliations:** ^1^School of Life Sciences, Huaibei Normal University, Huaibei, China; ^2^College of Horticulture, Nanjing Agricultural University, Nanjing, China

**Keywords:** ALA, anthocyanin accumulation, apple fruit calli, differentially expressed genes, *MdMYB10*, *MdMYB9*, *MdMATE8*

## Abstract

The red color is an attractive trait of fruit and determines its market acceptance. 5-Aminolevulinic acid (ALA), an eco-friendly plant growth regulator, has played a universal role in plant secondary metabolism regulation, particularly in flavonoid biosynthesis. It has been widely reported that ALA can up-regulate expression levels of several structural genes related to flavonoid metabolism and anthocyanin accumulation. However, the molecular mechanisms behind ALA-induced expression of these genes are complicated and still far from being completely understood. In this study, transcriptome analysis identified the differentially expressed genes (DEGs) associated with ALA-induced anthocyanin accumulation. Kyoto Encyclopedia of Genes and Genomes (KEGG) analysis showed that the flavonoid biosynthesis (ko00941) pathway was significantly enhanced in the ALA-treated apple calli at 24, 48, and 72 h after the treatment. Expression pattern revealed that ALA up-regulated the expression of the structural genes related to not only anthocyanin biosynthesis (*MdCHS*, *MdCHI*, *MdF3’H*, *MdDFR*, *MdANS*, and *MdUFGT*) but also anthocyanin transport (*MdGST* and *MdMATE*). Two R2R3-MYB transcription factors (MdMYB10 and MdMYB9), which are the known positive regulators of anthocyanin biosynthesis, were significantly induced by ALA. Gene overexpression and RNA interference assays demonstrated that *MdMYB10* and *MdMYB9* were involved in ALA-induced anthocyanin biosynthesis. Moreover, MdMYB10 and MdMYB9 might positively regulate the transcription of *MdMATE8* by binding to the promoter region. These results indicate that *MdMYB10* and *MdMYB9* modulated structural gene expression of anthocyanin biosynthesis and transport in response to ALA-mediated apple calli coloration at the transcript level. We herein provide new details regarding transcriptional regulation of ALA-induced color development.

## Introduction

Fruit coloration acts as one of the most important factors determining commodity value of red cultivars. Finding eco-friendly, efficient, and easy-to-operate methods to improve fruit color has long been a major concern of fruit researchers. 5-Aminolevulinic acid (ALA), a key precursor in tetrapyrrole biosynthesis, has drawn increasing attention due to its important regulation roles in multiple physiological processes, including plant development, secondary metabolism, and fruit ripening (Akram and Ashraf, 2013). Promoting red fruit coloration is one of the most outstanding roles of ALA, which has been widely demonstrated in apple (Wang et al., 2006; Xie et al., 2013; Zheng et al., 2017), grape (Watanabe et al., 2006), pear (Xiao et al., 2012), peach (Guo et al., 2013; Ye et al., 2017), litchi (Feng et al., 2015), and strawberry (Li et al., 2016), indicating great application potential of ALA in modern fruit production. However, the molecular mechanism underlying ALA-induced fruit coloration is largely unknown.

The red pigment of fruit is mainly caused by anthocyanin, which is the major component of flavonoids. Anthocyanin is synthesized from the cytoplasmic face of endoplasmic reticulum in the cytosol and then transported into the vacuoles (Bae et al., 2006). In plant, anthocyanin biosynthesis starts from phenylalanine and is genetically catalyzed by the key enzymes encoded by structural genes, including *PAL*, *CHS*, *CHI*, *F3’H*, *DFR*, *LDOX*/*ANS*, and *UFGT*. The common biosynthetic pathways of anthocyanin have been well characterized in *Zea mays* (Wienand et al., 1986), petunia (Britsch and Grisebach, 1986), snapdragon (Martin et al., 1991), *Arabidopsis thaliana* (Shirley et al., 1995), and other plant species. Subsequently, a number of flavonoid biosynthesis pathways have been characterized in fruit crops, including apple (Takos et al., 2006), pear (Fischer et al., 2007), grape (Sparvoli et al., 1994), and strawberry (Pillet et al., 2015). Pigment also associated with anthocyanin transport. It was described that several members of glutathione *S*-transferase (GST) and the multidrug and toxic compound extrusion (MATE) family were involved in anthocyanin transport, such as *Zea mays Bronze2* (*bz2*) (Marrs et al., 1995), petunia *AN9* (Mueller et al., 2000), *Arabidopsis TT12* and *TT19* (Debeaujon et al., 2001), and grape *AM1* and *AM3* (Gomez et al., 2009). With regard to the preliminary mechanism, several studies suggest that ALA-promoted anthocyanin accumulation is closely related to higher expression levels of flavonoids biosynthetic genes, including *CHS*, *DFR*, *ANS*, and *UFGT* (Guo et al., 2013; Xie et al., 2013; Feng et al., 2016; Ye et al., 2017).

In general, the structural genes of anthocyanin biosynthesis are coordinately regulated at transcriptional level by a MBW ternary complex of MYB, basic helix-loop-helix (bHLH) domains, and WD40 proteins (Xu et al., 2015). In higher plants, MYB transcription factors (TFs) are one of the largest TF families characterized by the conserved MYB DNA-binding domain. R2R3 subfamily with two adjacent MYB domains are the principal member of MYB, which plays a crucial role in secondary metabolism, stress response, meristem formation, and the cell cycle (Martin and Paz-Ares, 1997). Based on the conservation of amino acid motifs in C terminal domains and of the DNA-binding domain, R2R3-MYB proteins have been divided into 25 subgroups (SG) in *Arabidopsis* (Dubos et al., 2010). In apple, 229 MYB protein families, containing 222 typical R2R3 MYB proteins, were subdivided into 45 subgroups (Cao et al., 2013). Among them several SGs were involved in the regulation of flavonoid biosynthesis. The SG6 mainly associated with anthocyanin biosynthesis and accumulation by activating structural genes, such as *AtMYB75/PAP1*, *AtMYB90/PAP2*, *AtMYB113*, and *AtMYB114* in *Arabidopsis* (Gonzalez et al., 2008), *PhAN2* in petunia (Quattrocchio et al., 1999), *PpMYB10.3* in peach (Zhou et al., 2015, 2016), *PyMYB10* in pear (Feng et al., 2010), and *MdMYB10/MdMYB1/MdMYBA* in apple (Takos et al., 2006; Ban et al., 2007; Espley et al., 2007). Members of SG5 were suggested responsible for proanthocyanidins (PAs) synthesis, including *AtMYB123/TT2* (Nesi et al., 2001), *FaMYB9* and *FaMYB11* (Schaart et al., 2013), *VvMYBPA2* (Terrier et al., 2009), *MdMYB11* (An et al., 2015), and *MdMYB12* (Wang et al., 2017). Moreover, MdMYB9, belonged to the SG5 subfamily, bound to the promoters of *ANS*, *ANR*, and *LAR* and promoted the accumulation of anthocyanins and PAs (An et al., 2015). SG7 typically regulating flavonol synthesis were described in *Arabidopsis* (Stracke et al., 2007), grape (Czemmel et al., 2009), and apple (Wang et al., 2017). In addition, some R2R3-MYB proteins of SG4, which encode transcriptional repressors, also exhibited different effects on flavonoids synthesis. In transgenic tobacco, overexpression of *MdMYB3* resulted in anthocyanin accumulation and pigmentation (Vimolmangkang et al., 2013). In contrast, *FaMYB1* acted as the transcriptional repressors to reduce expression of late flavonoid biosynthesis genes in tobacco (Aharoni et al., 2001). To date, ALA-promoted expression of anthocyanin biosynthetic genes has also been linked to several regulatory genes. *MdMYB10* and *MdbHLH33* appeared to play positive roles in ALA-induced anthocyanin accumulation in apple (Zheng et al., 2018), while in peach skin, ALA significantly activated the expression of *PpMYB10* and *PpWD40* but not *PpbHLH3* (Ye et al., 2017). It seems that R2R3-MYB TFs of SG6 respond to ALA-stimulated coloration, but the directly conclusive evidences are limited. Thus, transcriptional network is necessary for us to better understand the molecular mechanism of ALA-induced anthocyanin accumulation.

Under artificial condition, calli can be continuously and uniformly produced to efficiently occupy the available space without seasonal restrictions and also provide a homogeneous system observation and analysis of different treatment effects. Lately, calli was widely used as the model system to identify key candidate genes’ functions and elucidate the mechanism of PGRs on plant growth regulation, especially in flavonoid metabolism (Ji et al., 2015; Sun et al., 2017; Premathilake et al., 2020). Therefore, in the present study, apple fruit calli undergo a transcriptome profiling analysis to identify the differentially expressed genes (DEGs) between the ALA-treated and untreated control apple calli at three illuminating time points. Two key candidate TFs identified by RNA-seq were then proved to regulate ALA-induced coloration in different transgenic cell lines. Our findings enrich the knowledge regarding the molecular mechanism behind ALA-improved fruit coloration, which can substantially accelerate the study of ALA functions and its application in modern fruit production.

## Materials and Methods

### Plant Materials and Sample Treatment

The apple calli used in this study were induced from the flesh of “Fuji” apple (*Malus domestica* Borkh.), which were collected from commercial apple orchards of eastern China, Fengxian in Jiangsu Province. Calli were grown on MS medium (1 mg L^–1^ 6-BA and 1 mg L^–1^ 2, 4-D) at 22°C in dark, and were subcultured at 20-day intervals (An et al., 2015). For sample treatment, calli cultured in separate flasks were gathered and randomly transferred to liquid medium with (ALA) or without (Control) 50 mg L^–1^ ALA for 3 h in a shaking incubator in dark. For anthocyanin induction, all samples were re-transferred to MS solid medium and cultured at 22°C under light of 100 μmol m^–2^ s^–1^ photon flux density. Calli were harvested after being illuminating for 24, 48, and 72 h, respectively. At each time point, approximate 3 g apple calli were sampled after light irradiation and prepared three replicates for each treatment. A total of 18 independent libraries were subjected to transcriptome sequencing. Control calli were named as Control-24 (including C1, C2, and C3; C, Control), Control-48 (including C7, C8, and C9), and Control-72 (including C13, C14, and C15). ALA-treated calli were named ALA-24 (including T4, T5, and T6; T, treatment), ALA-48 (including T10, T11, and T12), and ALA-72 (including T16, T17, and T18).

### Extraction and Determination of Anthocyanin Content

Anthocyanin content in calli was measured according to Xie et al. (2013) with slight modifications. Calli were incubated in 1% (v/v) HCl-methanol for 24 h at room temperature in dark. After centrifugation at 8000 × *g* for 15 min, the absorbance of upper aqueous phase was measured at 530, 620, and 650 nm with a spectrophotometer. The content of anthocyanin was expressed as nmol of cyanidin-3-galactoside in 1 g of fresh sample using a molar extinction coefficient of 3.43 × 10^4^ (Ubi et al., 2006). Mean values were obtained from three independent replicates.

### RNA Extraction, Library Preparation, and RNA-Seq Analysis

Total RNA was isolated from calli using the RNAprep pure Plant Kit (Tiangen, Beijing, China) following the manufacturer’s instructions. DNase I was used for treating total RNA samples, and then mRNA was purified from total RNA using poly-T oligo-attached magnetic beads. The quality of RNA was examined by an Agilent 2100 Bioanalyzer (Agilent Technologies, Palo Alto, CA, United States). About 3 μg of high-quality RNA per sample was used to construct the RNA-seq libraries, and a total of 18 libraries (six for per time point) were generated using NEBNext^®^ Ultra^TM^ RNA Library Prep Kit. The library preparations were sequenced for paired-end reads with the HiSeq X system (Illumina, San Diego, CA, United States) by Novogene (Beijing, China). The Illumina raw data have been deposited in the NCBI sequence read archive (SRA) database under accession number PRJNA525304.

To obtain the high-quality reads (clean reads), raw RNA-seq reads were filtered by removing the adaptor, reads with unknown sequences “*N*” > 10%, and low-quality reads (Qphred ≤ 20 bases). Clean reads were then mapped to apple genome (*Malus domestica* v3.0.a1) using TopHat v2.0.12 (Trapnell et al., 2009; Velasco et al., 2010). HTSeq v0.6.1 was used to count the reads numbers mapped to each gene (Anders et al., 2014). Gene expression levels were estimated with FPKM (Fragments Per Kilobase of transcript per Million mapped reads) (Trapnell et al., 2010).

To identify DEGs between the control and ALA treatment, read counts as imputing data were normalized by DESeq (Anders and Huber, 2010). False discovery rate (FDR) was controlled by *p* values adjusted according to Benjamini and Hochberg’s approach. Genes with an absolute value of the log_2_ (Fold Change) ≥ 1 and FDR < 0.05 were identified as DEGs. The principal component analysis (PCA) was conducted with the internal steps of the *R* package version 3.5.3^1^. GOseq *R* package was used to analyze Gene Ontology (GO) enrichment of DEGs (Young et al., 2010). GO terms with a corrected *p* < 0.05 were considered significantly enriched. The KOBAS software was applied to identify the significantly enriched KEGG pathways among the DEGs (Mao et al., 2005).

### Phylogenetic Analysis

All the phylogenetic trees were generated using the maximum-likelihood (ML) method with 1000 bootstraps in MEGA X^2^. The accession numbers and sequences of MYB transcription factors in multiple plant species that were included in the phylogenetic analysis are listed in Supplementary Table 1. The sequences of 21 MdMATE members of clade I and 24 different known MATEs are listed in Supplementary Table 2.

### Construct Expression Vectors and *Agrobacterium*-Mediated Transformation System of Apple Calli

For overexpression of *MdMYB10* and *MdMYB9*, their CDSs were amplified by PCR from cDNA of “Fuji” apple fruit flesh calli. The PCR product was cloned into the pBI121 plant transformation vector downstream of the CaMV 35S promoter. To silence *MdMYB9*, *MdMYB10*, and *MdMATE8* expression, a partial CDS of *MdMYB10* (421 bp), *MdMYB9* (507 bp), and *MdMATE8* (429 bp) was transferred to RNAi vector (pHELLSGATE2, pHG2) through Gateway BP reaction (Invitrogen). The recombinant and empty plasmids were transformed into competent *A. tumefaciens* strain EHA105 using the freeze/thaw method. One single transformed *A. tumefaciens* colony was inoculated in 30 ml of YEB medium supplemented with corresponding antibiotics and grown at 28°C with shaking at 150 r.p.m. When OD_600_ of culture liquid reached approximately 0.5, *A. tumefaciens* cells were centrifuged at 4000 × *g* for 5 min, and resuspended in 10 ml of infiltration buffer (10 mM MES, pH 5.6, and 100 μM acetosyringone). The fresh calli were dipped into *A. tumefaciens* suspension for 15 min at room temperature. The calli were then co-cultured on solid MS medium containing 2,4-D and 6-BA for 3 days at 25°C in dark. Subsequently, the calli were retransferred to MS medium (250 mg L^–1^ carbenicillin and 30 mg L^–1^ kanamycin) for positive transgenic selection after being washed five to eight times with sterile water. The anthocyanin contents of calli were measured according to the method described above. The expression levels in apple transgenic calli lines were confirmed by qRT-PCR amplification. All assays were replicated at least three times.

### *Cis*-Acting Element Analysis

*Cis*-acting elements in the 1800-bp promoter region of *MdMATE8* were identified using the PlantCARE database^3^.

### Yeast One-Hybrid (Y1H) Assay

The promoter sequence (1800 bp) of *MdMATE8* (MDP0000175055) was inserted into the pHIS2.1 vector (Clontech, Palo Alto, CA, United States), which contained the reporter gene *HIS3*. The coding sequences (CDS) of *MdMYB10* and *MdMYB9* were cloned into the pGADT7 vector (Clontech) to form *pGADT7-MdMYB10* and *pGADT7-MdMYB9*, respectively. To detect the suitable concentration of 3-AT, the yeast strain Y187 transformed with the recombinant pHIS2 vectors were grown on SD/-Trp/-His medium containing different concentrations of 3-amino-1,2,4-triazole (3-AT). The constructs p53HIS2.1 and pGADT7-53 were used as the positive control. Negative controls were transformed with p53HIS2.1 + pGADT7, p53HIS2.1 + pGADT7-MdMYB10, p53HIS2.1 + pGADT7-MdMYB9, or pHIS2.1-MdMATE8pro + pGADT7, respectively. The transformants were selected on double dropout medium (DDO, SD/-Trp-Leu) media. The transformed yeast cells were plated onto triple dropout medium (TDO, SD/-Trp-Leu-His) containing 80 mM 3-AT, and cell growth was examined.

### Total RNA Isolation and Quantitative PCR Expression Analysis

As for qPCR assay, total RNA was extracted using the cetyltrimethylammonium bromide (CTAB) method. The first strand of cDNA synthesis was performed using the TransScript^®^ One-Step gDNA Removal and cDNA Synthesis Supermix (Transgen Biotech, China). The qRT-PCR reaction system was performed utilizing ChamQ SYBR qPCR master mix (Vazyme, Nanjing, China). The *MdUBQ* served as the housekeeping gene, and relative expressions of genes were calculated using the 2^−ΔΔCT^ method (Livak and Schmitten, 2010). Each sample was quantified in triplicate. All qRT-PCR primers are listed in Supplementary Table 3.

### Statistics Analysis

All of the results were collected from at least three independent parallel experiments. Statistical analysis was carried out using SPSS 20.0 statistical program. Significant differences (*p* < 0.05) of data were compared with control or among treatments by analysis of variance (ANOVA) and Duncan’s multiple range test. Graphs were generated in Excel 2016 and Origin Pro 7.5.

## Results

### Rapid Anthocyanin Accumulation in Apple Flesh Calli After ALA Treatment

**FIGURE 1:**
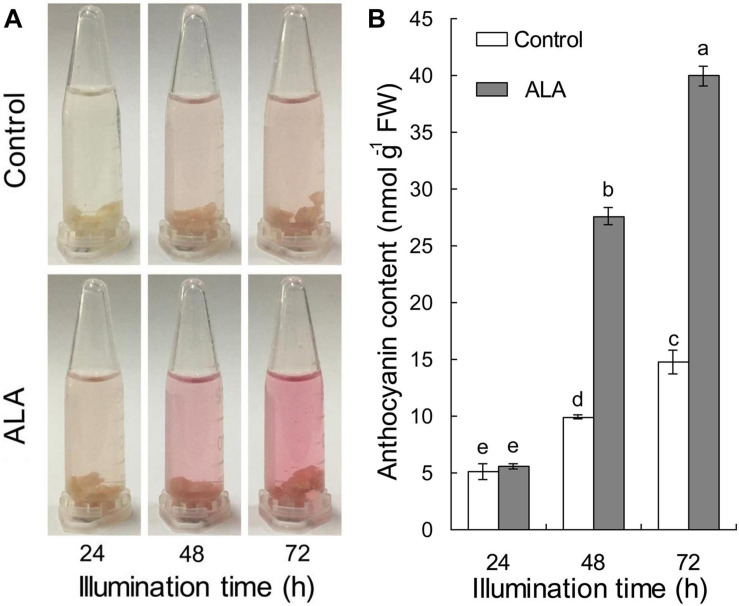
Time courses of anthocyanin accumulation after ALA treatment in fruit calli. The calli were incubated with 50 mg L^–1^ ALA or deionized water (Control) for 3 h under dark and then cultured in solid MS medium under light of 100 μmol m^–2^ s^–1^ photon flux density at 22°C for 72 h. **(A)** The process of color development in calli under light. **(B)** Anthocyanin content in calli. The different small letters represent significant differences (*p* < 0.05).

To verify the effect of ALA on coloration development, the apple flesh calli were transferred to liquid Murashige and Skoog (MS) medium with (ALA) or without (Control) 50 mg L^–1^ ALA for 3 h in a shaking incubator in dark, and calli anthocyanin content was measured after being illuminated for 24, 48, and 72 h, respectively. Results showed that red color cumulatively increased in calli with time, and ALA exhibited an attractively promotive effect on coloration after illuminating for 48 h (Figure 1A). The anthocyanin content of ALA-treated calli did not significantly increase at 24 h, but reached 2.78- and 2.70-fold, respectively, of the control at 48 and 72 h (Figure 1B). These results suggest that ALA significantly induces anthocyanin accumulation in apple flesh calli. Therefore, this artificial calli system can be used to identify the candidate genes that are related to ALA-induced anthocyanin accumulation.

### Overview of RNA Sequencing

A total of 514,483,442 raw reads were obtained from ALA-treated and control calli and were sampled at 24, 48, and 72 h. After quality filtering process, the number of clean reads for each library ranged from 22.7 to 32.0 million and the rate of clean reads/raw reads ranged from 93.82 to 96.26%. The Q30 values for all libraries were above 89.2% (Supplementary Table 4). An average of 90.62% reads were mapped to apple reference genome sequence, and 83.0% to 84.9% reads were mapped to exon region. Additionally, the correlation coefficient analysis showed that biological replicated libraries for two treatments per illumination time had highly consistent transcriptome profiles (Supplementary Table 4). These results reveal that the data we obtained are reliable and qualified for the following analysis.

### Analysis of DEGs

**FIGURE 2:**
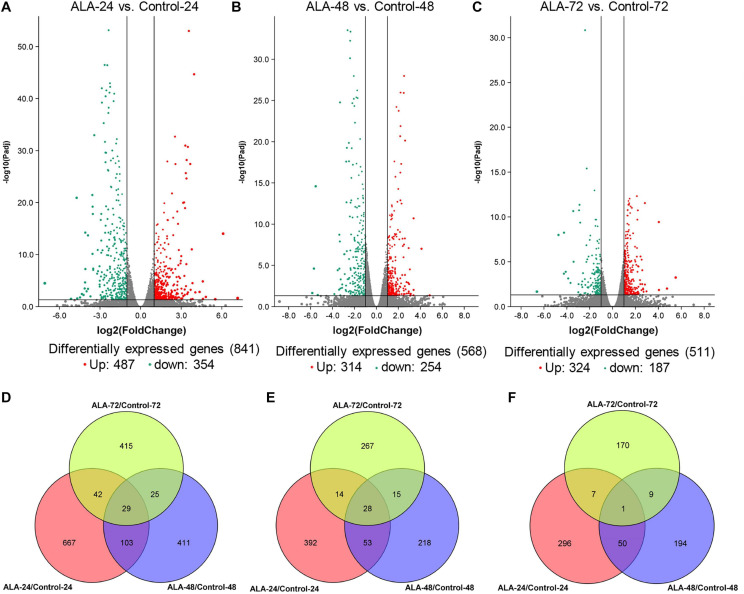
Differentially expressed genes (DEGs) identified by RNA-seq analysis in ALA-treated and control calli after illuminating for 24, 48, and 72 h. **(A–C)** Volcano plot of the RNA-seq showing DEGs in red and green. The *X*-axis represents the fold change in ALA-24 versus Control-24, ALA-48 versus Control-48, and ALA-72 versus Control-72, respectively. The *Y*-axis represents the negative –log_10_-transformed *p* values (*p-adj* < 0.05) for differences between the samples. **(D)** Quantity of total DEGs. **(E)** Quantity of up-regulated DEGs. **(F)** Quantity of down-regulated DEGs. Overlapping areas shows the shared DEGs at different time points.

To investigate differential gene expression between ALA-treated and control apple calli, gene expression levels were calculated based on the FPKM values (Supplementary Table 5). The DEGs were identified and filtered by a threshold of FDR ≤ 0.05 and an absolute Log_2_ (fold-change) value ≥ 1. A total of 1692 DEGs were identified as DEGs between ALA-treated and control calli (Supplementary Table 6). Except for the control calli illumination at 72 h, the PCA of the transcriptomic data revealed a high similarity among the three biological replicates within each treatment (Supplementary Figure 1). Among treatments, a clear separation of the ALA treatments from the control calli was observed. At 24 h after the treatment, the expression level of 487 and 354 genes were up- and down-regulated, respectively, in ALA-treated apple calli compared with control levels (Figure 2A). The expression level of 314 and 254 genes was up- and down-regulated, respectively, in ALA-treated apple calli relative to the corresponding control levels at 48 h after the treatment (Figure 2B). Furthermore, the expression level of 324 and 187 genes was up- and down-regulated, respectively, in ALA-treated apple calli versus control levels at 72 h (Figure 2C). More DEGs were found at 24 h between control and ALA-treated calli. DEGs among different time points were also identified. A total of 667, 411, and 415 DEGs were exclusively detected at 24, 48, and 72 h, respectively, suggesting that these genes might be involved in ALA-regulated physiology at a specific stage (Figures 2D–F). There were 29 common DEGs in all three illumination stages. Among these DEGs, 16 structural genes involved in flavonoid metabolism were identified (Figures 2D–F). These results imply that ALA stimulates significant changes of transcriptional level.

### GO Annotation and KEGG Pathway Analyses

**FIGURE 3:**
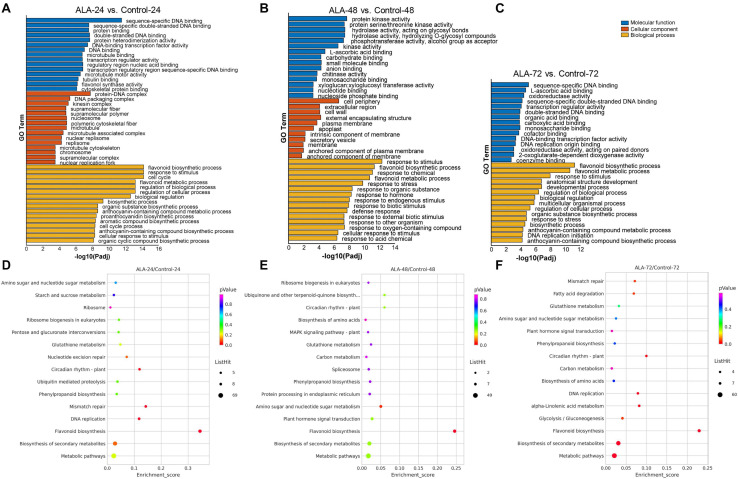
Gene ontology (GO) classifications and KEGG pathways enrichment of DEGs. **(A–C)** GO categories assigned to DEGs in ALA-24 versus Control-24 (A), ALA-48 versus Control-48 **(B)**, and ALA-72 versus Control-72 **(C)**, respectively. The *X*-axis represents the negative –log_10_-transformed *p* values (*p-adj* < 0.05) for differences between the samples. The left *Y*-axis shows categories according to the annotation of GO. **(D–F)** KEGG pathways of DEGs in ALA-24 versus Control-24 **(D)**, ALA-48 versus Control-48 **(E)**, and ALA-72 versus Control-72 **(F)**, respectively. The *Y*-axis and *X*-axis present the KEGG pathways and the enrichment scores, respectively. Dot size corresponds to the number of distinct genes, whereas dot color reflects the *p-adj* value.

Gene ontology (GO)-based term classification was performed to provide insights into DEG function. Among the total of 1692 DEGs, 1293 genes were annotated according to the GO database and were classified as “biological process,” “cellular component,” and “molecular function” (Figures 3A–C and Supplementary Table 7). However, the enriched items did not cover cellular components in the calli samples of ALA-72 versus Control-72 (Figure 3C). The two largest subcategories in the “biological process” category were “response to stimulus” including 523 DEGs and “biological regulation” including 466 DEGs. In addition, the subcategory of “flavonoid biosynthetic process” were notably enriched in the “biological process” at all three time points (Figures 3A–C). For the “cellular component” category, “membrane” including 506 DEGs and “intrinsic component of membrane” including 361 DEGs were the most abundant classes. Among the “molecular function” category, the two largest subcategories were “small molecule binding” and “anion binding” and included 346 and 340 DEGs, respectively (Supplementary Table 7).

To further understand the biological pathways activated by ALA at each time point, Kyoto Encyclopedia of Genes and Genomes (KEGG) pathways enrichment were conducted. Corresponding to GO enrichment, the KEGG pathway analysis showed that the flavonoid biosynthesis (mdm00941) pathway was significantly enhanced in ALA-treated calli at all three time points after the treatment (Figures 3D–F). A total of 21, 15, and 14 DEGs were enriched in flavonoid biosynthesis at 24, 48, and 72 h, respectively (Supplementary Table 8). Among them, 11 DEGs were significantly enriched at all three time points. These results imply that flavonoid biosynthesis pathway significantly responds to ALA stimulation.

### DEGs Related to Anthocyanin Biosynthesis and Transport

**FIGURE 4:**
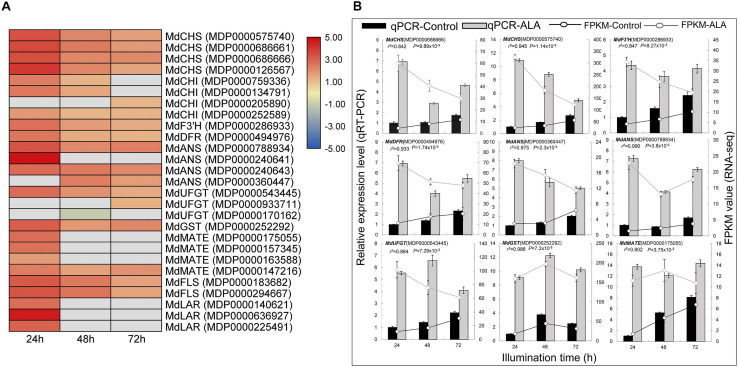
Expression pattern of genes involved in flavonoid biosynthesis and transport. **(A)** Heat map representation of transcriptional profiles of DEGs in the flavonoid biosynthesis pathway. The depth of color represents the changes of the value of log_2_ [fold change (FC)], and red represents up-regulation and blue represents down-regulation. The gray represents the relative differential expression level of those genes that did not reach screening threshold of DEGs in one or two time points after ALA treatment. **(B)** Verification of the expression of DEGs in the flavonoid biosynthesis pathway by qRT-PCR.

Anthocyanin biosynthesis is a dynamic and complex process catalyzed by multiple enzymes in the phenylpropanoid pathway. Here, 17 DEGs known to be involved in anthocyanin biosynthesis were identified according to GO and KEGG enrichment analysis (Figure 4). For example, *MdCHS* (MDP0000126567, MDP0000575740, MDP0000686661, and MDP0000686666), *MdCHI* (MDP0000134791, MDP0000252589, MDP0000759336, and MDP0000205890), and *MdF3’H* (MDP0000286933) were identified as the early biosynthetic genes (EBGs) in the early steps of the flavonoid biosynthesis pathway. Additionally, *MdDFR* (MDP0000494976), *MdANS* (MDP0000788934, MDP0000240641, MDP0000240643, and MDP0000360447), and *MdUFGT* (MDP0000543445, MDP0000933711, and MDP0000170162) were annotated as the late biosynthetic genes (LBGs) that were specifically involved in anthocyanin biosynthesis. Except for *MdUFGT* (MDP0000170162), which was down-regulated, 16 DEGs were significantly up-regulated in the ALA-treated apple calli, especially at 24 h after the treatment (Figure 4A). These results indicate that high expression levels of these structural genes in ALA-treated calli might be the main reason leading to anthocyanin accumulation.

Flavonoid transporter also plays a vital role in flavonoid accumulation. The MDP0000252292, annotated as *MdGSTF6*, was up-regulated in ALA-treated calli, which has been proven to play a role in anthocyanin transporter of apple peel (Jiang et al., 2019). We also identified four *MdMATE* genes that were more highly expressed in ALA-treated calli, indicating that these genes might be involved in ALA-induced flavonoid accumulation (Figure 4A).

Moreover, two *MdFLS* (MDP0000183682, and MDP0000294667) and three *MdLAR* (MDP0000140621, MDP0000636927, and MDP0000225491) were identified, suggesting that ALA may also affect flavonol and proanthocyanidin (PA) biosynthesis (Figure 4A).

In addition, we noticed that several structural genes could not respond to ALA-induced flavonoid metabolism at all three illumination time points, such as *MdCHI* (MDP0000759336 and MDP0000134791), *MdANS* (MDP0000240641 and MDP0000360447), *MdUFGT* (MDP0000933711 and MDP0000170162), *MdMATE* (MDP0000175055, MDP0000157345, and MDP0000163588), and *MdLAR* (MDP0000140621, MDP0000636927, and MDP0000225491) (Figure 4A). Therefore, the results reveal that some genes of flavonoid metabolism respond to ALA only in specific time points.

To verify the sequencing data, the relative expression levels of selected structural genes were further analyzed by qRT-PCR. The expression levels of these EBGs and LBGs were up-regulated to above 4.5-fold by ALA treatment (Figure 4B). These results suggest that these structural genes may play vital roles in ALA-induced anthocyanin accumulation.

### Transcriptional Regulation of ALA-Induced Anthocyanin Metabolism

The changes of structural gene expression are controlled by transcriptional factors, which plays the key role in plant responding to external stimulates. In this study, a total of 171 DEGs predicted to encode transcription factors (TFs) were identified by iTAK software. The MYB family members were the predominant TF genes in response to the ALA treatment, followed by the AP2-EREBP, and WRKY. Moreover, bHLH family genes were also differentially expressed (Supplementary Table 9).

**FIGURE 5:**
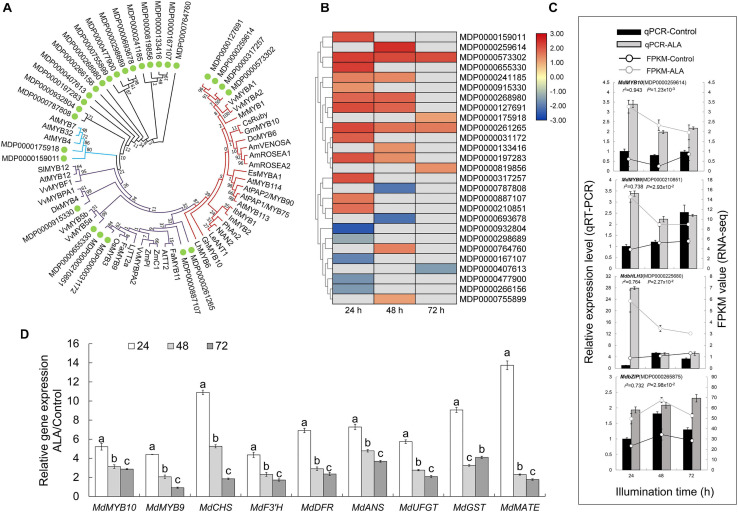
Analysis of differentially expressed MYB transcription factor genes related to putative flavonoid biosynthesis. **(A)** Phylogenetic tree with 39 phenylpropanoid pathway regulatory MYB TFs and 27 MYB from DEGs. **(B)** Heat map presenting the expression patterns of differentially expressed of 27 MYB. The depth of color represents the changes of the value of log_2_ [fold change (FC)], and red represents up-regulation and blue represents down-regulation. The gray represents genes that did not belong to DEGs at the corresponding time points. **(C)** Verification of the expression of four differentially expressed transcription factor genes by qRT-PCR analysis. **(D)** QRT-PCR analysis of the relative expression of *MdMYB10* and *MdMYBR9* and genes related to anthocyanin biosynthesis and transport in apple flesh calli at 24, 48, and 72 h after ALA treatment. The different letters in each gene represents significant differences (*p* < 0.05).

To further identify the key members of MYB family in the transcriptional regulation of ALA-induced anthocyanin accumulation in apple calli, a phylogenetic tree was constructed based on 39 selected R2R3-MYB proteins of phenylpropanoid pathway regulators and 27 MYB from DEGs (Figure 5A and Supplementary Table 1). Four candidate MYB transcription factors (MDP0000127691, MDP0000259614, MDP0000317257, and MDP0000573302) belonged to SG6, which phylogenetically related to positive regulators of anthocyanin biosynthesis such as VvMYBA1, VvMYBA2, and MrMYB1 (Niu et al., 2010; Rinaldo et al., 2015). Several candidate MYB TFs were revealed as the regulators of PA biosynthesis because they were grouped with SG5 MYB TFs including AtTT2, VvMYBPA2, FaMYB9, and OsMYB3. Additionally, two MYB TFs were identified as potential repressors, which branched with AtMYB4, AtMYB7, and AtMYB32 of SG4 (Fornalé et al., 2014).

The expression profiles of the 27 MYBs showed that most of these genes were more highly expressed in the ALA-treated apple calli than in the control (Figure 5B). Moreover, most members of SG4-6 MYB TFs predicted as the candidates involved in ALA-regulated flavonoid metabolism were up-regulated and peaked at 24 or 48 h after the treatment, indicating that these TFs could rapidly respond to ALA stimulation. The relative expression levels of four selected transcription factor genes determined by qRT-PCR were consistent with the FPKM values based on the RNA-sequencing (Figure 5C). In Figure 5B, although *MdMYB10* (MDP0000259614) and *MdMYB9* (MDP0000210851) were not screened as DEGs at two time points in RNA-seq, *MdMYB10* was up-regulated to 1.6-fold by ALA at 24 h (*p* < 0.05) and *MdMYB9* was improved to 0.8-fold at 48 h (*p* = 0.01) after ALA-treatment. These results indicated that the expression levels of these two TFs were induced by ALA treatment, which was basically consistent with our qRT-PCR results in Figure 5C.

Among 12 MYB TFs of SG4-6, *MdMYB10* (MDP0000259614 and MDP0000127691) and *MdMYB9* (MDP0000210851) were characterized as known anthocyanin regulatory MYB transcription factors in apple (An et al., 2015; El-sharkawy et al., 2015). Their expression profiles were positively correlated with structural genes of anthocyanin accumulation under ALA treatment (Figure 5D and Supplementary Table 10). These results demonstrate that *MdMYB10* and *MdMYB9* may play key roles in ALA-regulated coloration.

### Regulation Roles of *MdMYB10* and *MdMYB9* in ALA-Induced Anthocyanin Biosynthesis

To verify the roles of *MdMYB10* and *MdMYB9* in ALA-induced pigmentation, they were transiently overexpressed or silenced in “Fuji” apple fruit calli. The expression levels in their corresponding overexpression line were 12.4 and 6.7 times, respectively, higher than that in the control, which transformed the empty pBI121 plasmids (Supplementary Figures 2A,B). Moreover, exogenous ALA further up-regulated *MdMYB10* or *MdMYB9* expression level in their OE transgenic lines (Supplementary Figures 2A,B). In the RNA interference lines, the expression of *MdMYB10* and *MdMYB9* were approximately decreased to 61% and 55%, respectively, compared with the control, which was transformed with empty pHG2 plasmids (Supplementary Figures 2C,D). However, ALA did not significantly affect *MdMYB10* and *MdMYB9* expression in their RNAi calli. These results indicate that we obtain useable transformed apple calli of two key candidate genes.

**FIGURE 6:**
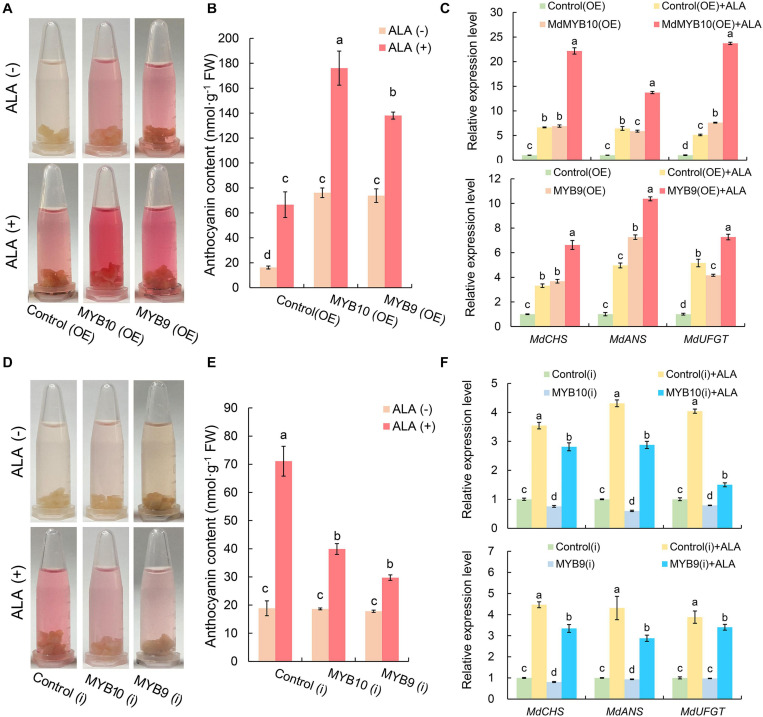
Effect of ALA on coloration level and anthocyanin content as well as the corresponding gene expression in *MdMYB10* and *MdMYB9* transgenic calli. Color **(A)** and anthocyanin content **(B)** in transgenic calli; OE, calli infiltrated with the plasmid for overexpressing of target genes; Control (OE), calli infiltrated with an empty pBI121 vector. **(C)** The expression of anthocyanin biosynthesis genes in *MYB10*(OE) and *MYB9*(OE). Color **(D)** and anthocyanin content **(E)** in transgenic calli; RNAi, calli infiltrated with the plasmid for silencing of target genes; Control(i), calli infiltrated with an empty pHG2 vector. **(F)** The expression of anthocyanin biosynthesis genes in *MYB10*(i) and *MYB9*(i). The different letters in each gene represent significant differences (*p* < 0.05).

Under light condition, the OE calli of *MdMYB10* and *MdMYB9* turned red more rapidly than the control(OE) (Figure 6A). Spectrophotometric analysis showed that *MdMYB10*(OE) and *MdMYBR9*(OE) accumulated significantly higher anthocyanin level, and the content was approximately 4.7 and 4.6 times, respectively, as high as that of the control(OE) calli (Figure 6B). Results confirmed their positive roles in anthocyanin biosynthesis. When exogenous ALA was applied, the color of *MdMYB10* and *MdMYB9* OE calli turned to deep red (Figure 6A), and the anthocyanin content further increased by 2.3 and 1.9 times, respectively, compared with each corresponding OE calli without ALA treatment (Figure 6B). Consistent with the color changes, overexpression of *MdMYB10* or *MdMYB9* up-regulated the expression level of structural genes of anthocyanin biosynthesis, and the effect was further promoted by ALA (Figure 6C). These results indicate that the promotive effect of ALA-induced anthocyanin accumulation is notably enhanced when *MdMYB10* or *MdMYB9* is overexpressed.

In the *MdMYB10* and *MdMYB9* RNAi lines, the coloration level and the anthocyanin content did not obviously change (Figures 6D,E), compared with control(i). ALA treatment also induced anthocyanin accumulation in *MYB10*(i) and *MYB9*(i); however, the promotive effects of ALA were markedly blocked and the anthocyanin content was 56% and 42%, respectively, of the control(i) treated with ALA (Figures 6D,E). The expression level of several structural genes decreased in the calli of *MYB10*(i) or *MYB9*(i) (Figure 6F). Compared with control(i) + ALA, silence of *MdMYB10* or *MdMYB9* significantly suppressed ALA-induced expression of structural genes (Figure 6F). These results demonstrate that ALA-induced anthocyanin biosynthesis is closely related to the normal transcriptional expression of *MdMYB10* and *MdMYB9*.

### *MdMATE8* Involved in ALA-Regulated Anthocyanin Accumulation

**FIGURE 7:**
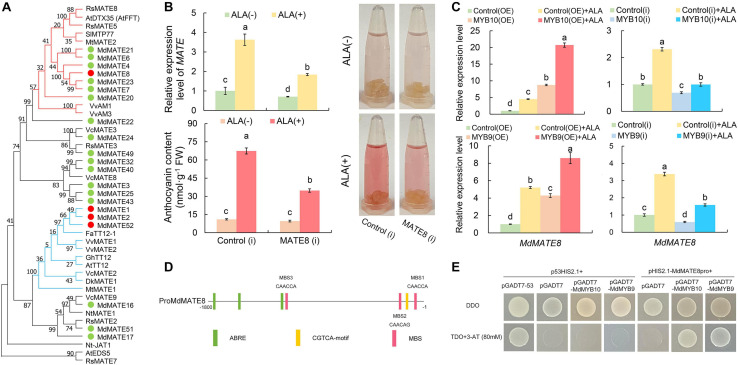
Functional characterization of *MdMATE8*. **(A)** Phylogenetic analysis of MdMATE proteins and selected MATE proteins from other species. **(B)** The expression levels, colors, and anthocyanin contents in the *MdMATE8* transgenic calli with or without ALA treatment. **(C)** Transcript levels of *MdMATE8* in *MdMYB10* and *MdMYB9* transgenic calli with or without ALA treatment. **(D)** The promoter region of *MdMATE8*. **(E)** Y1H assay showing the interaction between MdMYB10/MdMYB9 and the *MdMATE8* promoter. The constructs p53HIS2.1 + pGADT7-53 were used as the positive control. The constructs p53HIS2.1 + pGADT7, p53HIS2.1 + pGADT7-MdMYB10, p53HIS2.1 + pGADT7-MdMYB9, or pHIS2.1-MdMATE8pro + pGADT7 were used as the negative controls, respectively. The different letters in each gene represent significant differences (*p* < 0.05).

In anthocyanin accumulation pathway, GSTs and MATEs are putatively associated with the vacuolar sequestration of anthocyanins. Recently, it was proved that ALA promoted anthocyanin accumulation through *GST* pathway (Fang et al., 2020), which was consistent with differentially expressed *MdGSTF6* (MDP0000252292) in ALA-treated calli. Meanwhile, we also identified four *MdMATE* genes. To comprehensively analyze MATE transporter, the genome-wide analysis of the *MATE* genes in apple genome was conducted. At least 53 genes were initially isolated and named *MdMATE1* to *MdMATE53* according to their chromosomal information (Supplementary Figure 3A). A total of 53 MdMATE, 56 AtMATE, and 45 OsMATE were classified into four clades with ML (Maximum Likelihood) method (Supplementary Figure 3B). Some members of clade I were clustered with At3g59030 (AtTT12), and they may be involved in the transport of flavonoids (Debeaujon et al., 2001). Therefore, a phylogenetic analysis between 21 MdMATE members of clade I and 24 different known MATEs was conducted to further predict the functions of the MdMATE proteins (Figure 7A). One of the four *MdMATE* DEGs, named MdMATE8, was contained in seven MdMATE members, which were phylogenetically related to anthocyanin transporters such as MtMATE2 in barrel medic (*Medicago truncatula*) (Zhao et al., 2011), SlMTP77 in tomato (*Solanum lycopersicum*) (Mathews et al., 2003), and VvAM1 and VvAM3 in grapevine (*Vitis vinifera*) (Gomez et al., 2011), indicating that *MdMATE8* might be involved in the anthocyanin accumulation in the apple calli. In addition, the other three DEGs (*MdMATE1*, *MdMATE2*, and *MdMATE52*) were clustered together with PA-transporting MATEs, such as FaTT12-1, VvMATE1, VvMATE2, AtTT12, and MtMATE1 (Figure 7A), implying that these three *MdMATE* might function in ALA-regulated PA accumulation. Thus, we chose *MdMATE8* as the key candidate gene to analyze its molecular function in apple calli coloration and ALA-induced anthocyanin accumulation.

Transient assays with apple calli were performed to test the function of *MdMATE8*. RNAi significantly decreased the expression of *MdMATE8* to 58.5%, but the color levels and anthocyanin content did not change in comparison with control(i) calli (Figure 7B). However, the promotive effect of ALA on calli pigment was significantly impaired in the *MdMATE8*(i) transformed calli, and the anthocyanin content was 51.6% of the control(i) treated with ALA (Figure 7B). These results suggest that *MdMATE8* may play important roles in ALA-induced anthocyanin accumulation.

Furthermore, the expression levels of *MdMATE8* were detected in *MdMYB10* and *MdMYB9* transgenic calli lines (Figure 7C). Consistent with the anthocyanin content changes (Figures 7A,B), the expression level of *MdMATE8* was significantly up-regulated in *MdMYB10* or *MdMYB9* overexpression calli, but down-regulated in their RNAi calli. The relative expression levels of *MdMATE8* were more highly expressed in ALA-treated OE calli than in the control(OE) + ALA calli. However, the promotive capacity of ALA on expression of *MdMATE8* was obviously inhibited in ALA-treated *MdMYB10* or *MdMYB9* RNAi calli, compared with the control(i) + ALA calli. These findings indicate that *MdMYB10* and *MdMYB9* were probably involved in transcriptional expression regulation of *MdMATE8* in ALA-up-regulated anthocyanin accumulation.

To explore the region upstream of *MdMATE8*, the *MdMATE8* promoter sequence was analyzed using the PlantCARE online tools. Some hormone response elements were detected in the promoter, including ABA (ABRE) and MeJA (CGTCA-motif) response elements. In addition, several putative MYB *cis*-elements were found in the promoter regions of *MdMATE8*, including CAACCA and CAACAG, as illustrated in Figure 7D. With the results above, we speculated that MdMYB10 or MdMYB9 might affect the transcriptional activity of the *MdMATE8.* To validate this possibility, we conducted the Y1H assay. The results showed that the transformants harboring the plasmid of pHIS2.1-MdMATE8 could not grow on SD/-Trp/-His media containing 80 mM of 3-AT (Supplementary Figure 4). Screening was carried out on TDO medium containing 80 mM 3-AT (Figure 7E). Like the positive control cells containing p53HIS2.1 + pGADT7-53, the yeast cells transformed with pHIS2.1-MdMATE8pro + pGADT7-MdMYB10, or pHIS2.1-MdMATE8pro + pGADT7-MdMYB9 were able to grow on TDO medium with 80 mM 3-AT, whereas the negative control yeast cells (p53HIS2.1 + pGADT7, p53HIS2.1 + pGADT7-MdMYB10, p53HIS2.1 + pGADT7-MdMYB9, and pHIS2.1-MdMATE8pro + pGADT7) were able to grow only on DDO medium (Figure 7E). These results showed that MdMYB10 and MdMYB9 can bind to the *MdMATE8* promoter (Figure 7E). Therefore, all of these results suggest that MdMYB10 and MdMYB9 are involved in regulation of the expression of *MdMATE8* in response to the ALA stimulation.

## Discussion

### ALA Effectively Induces Anthocyanin Accumulation in Calli Culture System

In red cultivars, the coloration of fruit largely determines fruit nutritional and commercial value. It has been well-documented that ALA plays an effective role in promoting fruit color formation (Wang et al., 2006; Xiao et al., 2012; Guo et al., 2013; Xie et al., 2013; Feng et al., 2016; Ye et al., 2017; Zheng et al., 2017). In orchards, exogenously sprayed high concentration ALA on fruit clusters of litchi at 45 days after full bloom significantly increased the anthocyanin content (Feng et al., 2015). Likewise, both exogenous 300 mg L^–1^ ALA and bagging remarkably promoted “Yunhongli 2” coloration at the early stage of fruit coloration (Xiao et al., 2012). Recently, direct root irrigation of ALA also improved fruit coloration and nutrition quality, and this approach was recommended when encountering fruit bag barriers (Zheng et al., 2018). Under lab conditions, researches showed that exogenous ALA elevated anthocyanin accumulation in fruit cubes placed into the growth chamber (Xie et al., 2013). The above researches suggest that ALA has a promising application prospect in fruit production. However, the mechanisms underlying ALA-induced anthocyanin accumulation remain largely unknown. Here, the calli induced from apple flesh also showed significantly higher anthocyanin content under ALA treatment than control after illuminating at 48 and 72 h (Figure 1), providing a more efficient way to study the regulation roles of ALA on anthocyanin accumulation.

### ALA Rapidly Stimulates Transcriptome Changes

Secondary metabolites are catalytically synthesized by a series of enzymes in plant (Kim et al., 2004). Substantiality changes in the quantity and activity of these enzymes depend on regulation at the transcriptional or protein level (Xie et al., 2012; Yang et al., 2013; Jiang et al., 2014). Thus, regulation of transcription or protein levels is the fundamental reason of secondary metabolic rate changes. In litchi, the anthocyanin contents in pericarp remarkably increased after bags were removed 1–7 days; however, the greatest number of up-regulated DEGs was found between 0- and 1-day libraries (Zhang et al., 2016). Cyanidin-3-galactoside, the main cyanidin pigment in “Starkrimson” apple skin, rapidly accumulated at 4–8 days after bag was removed and peaked at 8 days, while the transcript levels of *MdMYB1-1*, *MdbHLH3-2*, and *MdUFGT4* increased immediately when exposed to light at 0–2 days and then gradually decreased (Meng et al., 2015). Therefore, there appears a time course discrepancy between mRNA or protein expression level and secondary metabolite accumulation. Here, we found similar results. The DEGs were enriched at 24 h after ALA treatment (Figures 2A–C), but anthocyanin significantly accumulated at 48 h and 72 h (Figure 1), implying that ALA-induced changes of gene expression levels may be the main reason for anthocyanin accumulation in calli. Moreover, the KEGG analysis revealed that the flavonoid biosynthesis pathway was significantly enhanced in the ALA-treated apple calli compared with the untreated control (Figures 3D–F). Thus, the transcriptional data we obtained provides valuable information on elucidating the mechanism behind ALA-regulated color formation.

### ALA Regulates Expressions of Structural Genes of Anthocyanin and Other Flavonoids Metabolism

Higher levels of anthocyanin are largely dependent on the higher transcript levels of a series of structural genes. Researches have reported that exogenous ALA up-regulated the expression levels of several key structural genes in the phenylpropanoid pathway (Guo et al., 2013; Xie et al., 2013; Feng et al., 2016; Ye et al., 2017; Zheng et al., 2018). Chalcone synthase (CHS), the key enzyme in the first committed step of the flavonoid biosynthetic pathway, catalyzes the stepwise condensation of 4-coumaroyl-CoA and malonyl-CoA into yellow chalcone (Martin, 1993). Exogenous ALA notably enhanced *CHS* gene expression in peach (Guo et al., 2013; Ye et al., 2017), apple (Xie et al., 2013; Feng et al., 2016; Zheng et al., 2017), and apple calli (Figure 4A). Leucoanthocyanidin dioxygenase (LDOX/ANS) and flavonoid 3-O-glucosyltransferase (UFGT) are the enzymes at the end of plant anthocyanin biosynthetic pathway, which catalyze colorless leucoanthocyanidins into colorful and stable anthocyanin (Kim et al., 2003). ALA strongly induced gene expression or enzyme activities of ANS and UFGT in peach (Guo et al., 2013), pear (Xiao et al., 2012), and apple (Xie et al., 2013). Similarly, here, both *ANS* and *UFGT* were increased by ALA at transcript levels (Figure 4A). Meanwhile, transcriptome analysis also showed that ALA significantly promoted expression levels of four *CHI*, one *F3’H*, and one *DFR* (Figure 4A). These results indicate that the biosynthetic genes of anthocyanin significantly respond to ALA induction.

For anthocyanin sequestration, several members of GST- and MATE-encoded enzymes are required for anthocyanin transport from cytoplasm to vacuole. Some members of *GST* have been identified to be responsible for anthocyanin transport in *Arabidopsis* (Kitamura et al., 2004), grapes (Gomez et al., 2011), litchi (Hu et al., 2016), peach (Zhao et al., 2020), and apple (El-sharkawy et al., 2015; Jiang et al., 2019). Some *MATE* family members also showed positive correlation with anthocyanin transport (Mathews et al., 2003; Marinova et al., 2007; Frank et al., 2011; Gomez et al., 2011; Zhao et al., 2011). In the present study, one *GST* and four *MATE* genes were up-regulated by ALA in calli (Figure 4A). Among them, *MdGSTF6* has shown the positive role in anthocyanin accumulation in apple and ALA-treated apple calli (Jiang et al., 2019; Fang et al., 2020). MdMATE8, an ortholog of MtMATE2 (Zhao et al., 2011), SlMTP77 (Mathews et al., 2003), and VvAM1 and VvAM3 (Gomez et al., 2011), was predicted as the anthocyanin transporter (Figure 7A). Transient expression assays showed that the RNA interference of *MdMATE8* notably inhibited anthocyanin accumulation in calli after ALA treatment (Figure 7B). These results suggest that *MdGSTF6* and *MdMATE8* may act as the key anthocyanin transporters for ALA-induced coloration.

In addition to anthocyanin, flavonol and proanthocyanidin are major subclasses of flavonoids. Anthocyanin and flavonol biosynthesis are produced from dihydroflavonol via two different branches, which are catalyzed by DFR or FLS, respectively. Overexpressing *DFR* or inactivation of *FLS* promoted anthocyanin accumulation (Davies et al., 2003; Stracke et al., 2009), while overexpression of *FLS1* decreased seed coat pigmentation (Nguyen et al., 2016), suggesting existence of the substrate competition between *FLS* and *DFR*. Interestingly, root application of ALA not only promoted anthocyanin accumulation but also increased flavonol content (Zheng et al., 2017). ALA-improved flavonol accumulation has also been reported in guard cells of apple leaves and *Arabidopsis* cotyledons (An et al., 2016; Liu et al., 2016). Here, our results further showed that ALA simultaneously increased the transcription level of *MdFLS* and *MdDFR* (Figure 4A). Meanwhile, the expression of the key genes involved in PA biosynthesis (*MdLARs*) was up-regulated by ALA (Figure 4A). Additionally, three *MATE* DEGs were grouped with PA transporters (Figure 7A), and both *MdMATE1* (MDP0000163588) and *MdMATE2* (MDP0000157345) showed functional similarity to TT12 from *A. thaliana* and acted as vacuolar flavonoid/H^+^-antiporters active in PA accumulating cells (Debeaujon et al., 2001; Frank et al., 2011). These results demonstrate that ALA not only induces anthocyanin accumulation but also may participate in regulating other flavonoids’ metabolism. The knowledge of ALA affecting flavonol and proanthocyanidin accumulation pathways require further research.

### MdMYB10 and MdMYB9 Are Involved in ALA-Induced Anthocyanin Accumulation in Apple Calli

Anthocyanin biosynthesis regulation particularly focuses on the MBW regulatory complex (Xu et al., 2015). R2R3-MYB TFs of SG6 predominantly play vital roles in modulation of anthocyanin metabolism in plant (Dubos et al., 2010). Among them, *AtPAP1*, *AtPAP2*, *AtMYB113*, and *AtMYB114* have been demonstrated to control anthocyanin biosynthesis in *Arabidopsis* seedling vegetative tissues (Gonzalez et al., 2008). *PhAN2* is predominantly correlated to anthocyanin biosynthesis in petunia flowers (Quattrocchio et al., 1999). Putative ortholog proteins of MYB10 of 20 different rosaceous fruits have been demonstrated to be involved in anthocyanin production (Lin-Wang et al., 2010). *PyMYB10* has been characterized as being associated with anthocyanin biosynthesis in red-skinned pear cultivars (Feng et al., 2010). In strawberry, *FaMYB10* activated the early and late steps of phenylpropanoid and flavonoid biosynthesis (Medina-Puche et al., 2014). In apple, it has been proposed that *MdMYB1/A* were responsible for the red coloration of the apple peel, while *MdMYB10* mainly for the whole fruit as well as foliage (Peng and Moriguchi, 2013). Therefore, *MdMYB10* is involved in anthocyanin biosynthesis, mainly in apple flesh. In the present study, we found that *MdMYB10* expression was significantly increased and positively correlated with EBGs and LBGs of anthocyanin biosynthetic pathway in ALA-tread calli (Figures 5B,D). This result suggests that *MdMYB10*, a typical SG6 anthocyanin regulator, may be directly responsible for ALA-induced anthocyanin biosynthesis. Here, another typical R2R3-type MYB of SG5, *MdMYB9*, was screened out as a potential positive regulator mediated ALA-triggered color development (Figures 5A,B,D). In “Orin” apple transgenic calli, MdMYB9 has been functionally characterized as a positive regulator of MeJA-induced anthocyanin and PA production (An et al., 2015). Recent researches showed that MdMYB9 was involved in several TFs that regulated anthocyanin and PA biosynthesis in apple, such as MdNAC52 (Sun et al., 2019), MdBBX37 (An et al., 2020), and MdERF1B (Zhang et al., 2018). Therefore, *MdMYB9* may also play a role in ALA-regulated anthocyanin accumulation.

On the basis of transient assays in apple calli, we confirmed that *MdMYB10* and *MdMYB9* are responsible for controlling anthocyanin biosynthesis in apple fruit (Figures 6A,B) (Espley et al., 2007; An et al., 2015). Previous studies observed that *MdMYB10* and *MdMYB9* could directly bind to the promoter region of *MdDFR* and *MdANS*, respectively (Espley et al., 2007; An et al., 2015). In the present study, overexpression of *MdMYB10* and *MdMYB9* significantly activated several key genes of the anthocyanin biosynthesis, and the effect was further enhanced in ALA-treated calli (Figures 6A–C), which may associate with ALA up-regulated *MdMYB10* and *MdMYB9* expression level in their OE transgenic lines (Supplementary Figures 2A,B). The results imply that several upstream TFs of *MdMYB10* and *MdMYB9* could respond to ALA-activated coloration to induce endogenous *MdMYB10* and *MdMYB9* expression in their OE calli. Thus, it seems that overexpression of *MdMYB10* and *MdMYB9* achieved by the 35S promoter could not have feedback to inhibit ALA-induced endogenous *MdMYB10/9* gene expression. In contrast, the promotive roles of ALA on expression of *MdCHS*, *MdANS*, and *MdUFGT* were obviously suppressed in *MdMYB10* or *MdMYB9* silenced calli (Figure 6F). Therefore, *MdMYB10* and *MdMYB9* are essential for ALA-up-regulated expression of EBGs and LBGs of anthocyanin biosynthesis during calli coloration. Interestingly, ALA still up-regulated these structural gene expression levels in calli of two TFs RNAi lines (Figure 6F). These results suggest that ALA-induced structural gene expression partially depends on *MdMYB10* and *MdMYB9*. In fact, MYB9 group is a rare type of anthocyanin-related MYB activator, which is lost in most dicot species (Zhou et al., 2016), whereas ALA is able to activate anthocyanin biosynthesis in various fruit species (Watanabe et al., 2006; Xiao et al., 2012; Guo et al., 2013; Feng et al., 2015; Li et al., 2016; Ye et al., 2017), suggesting that ALA-induced accumulation of anthocyanin is coordinately regulated by a set of R2R3-MYB genes. Moreover, among the DEGs of TFs, we also identified *bHLH*, *AP2-EREBP*, *WRKY*, *bZIP*, and *NAC* family members (Supplementary Table 9). These DEGs of TFs possibly function in ALA-activated coloration and will be clarified in further researches.

Additionally, our DEG data found that *MdbHLH3* (MDP0000225680) was dramatically induced in 24 h under ALA treatment (Figure 5C and Supplementary Table 6). In apple calli, we also found that *MdbHLH3* expression was up-regulated after ALA treatment (Zheng et al., 2018). An et al. (2015) found that MdbHLH3 could be recruited to the promoter regions of *MdMYB9* to activate expression. Thus, it is reasonable to speculate that the *MdMYB9* expression was initiated by MdbHLH3 in response to ALA. Importantly, MdbHLH3 was required for the regulatory activity of MdMYB10 and MdMYB9 (Xie et al., 2012; An et al., 2015). In *MdMYB10* and *MdMYB9* OE and RNAi transgenic calli, the anthocyanin content and selected structural gene expression level of ALA-treated calli were higher than that without ALA-treated calli (Figures 6A–F). We deem that *MdbHLH3* up-regulated by ALA can active *MdMYB9* expression or form more complexes of transcriptional regulation with MdMYB10 or MdMYB9 to bind to the promoters of structural genes and ultimately effectively promote anthocyanin accumulation in transgenic calli lines. The function of *MdbHLH3* in ALA-induced coloration needs to be further conducted.

Compared with the intensive studies of the regulatory and structural genes involved in anthocyanin biosynthesis, the molecular mechanism associated with anthocyanin transport from cytoplasm to vacuole remains uncertain, especially in ALA-mediated anthocyanin accumulation. Recently, researches showed that anthocyanin transport is regulated by MBW complexes. In peach, transient overexpression of *PpGST1* together with *PpMYB10.1* led to much deeper coloration as compared with *PpMYB10.1* alone (Zhao et al., 2020). In apple, the expression of *MdGSTF6* was activated by MdMYB1 (Jiang et al., 2019). AtTT2 was also able to induce the *AtTT12* promoter activity (Xu et al., 2014). Here, our results revealed that *MdMYB10* and *MdMYB9* showed high expression relation with *MdMATE8* in ALA-stimulated color developing calli (Figure 5D). Overexpression of *MdMYB10* and *MdMYB9* up-regulated the expression of *MdMATE8*, but the expression was decreased in *MdMYB10* or *MdMYB9* RNAi calli (Figure 7C). Moreover, through Y1H screening, we found that MdMYB10 and MdMYB9 can bind to the *MdMATE8* promoter (Figure 7E), indicating that MdMYB10 and MdMYB9 may activate *MdMATE8* transcription by binding to its promoter. These findings provide a new understanding that ALA strongly up-regulates the expression of MdMYB10 and MdMYB9 to regulate anthocyanin biosynthesis as well as transport, thereby promoting anthocyanin accumulation.

## Conclusion

In the current study, exogenous ALA-activated anthocyanin accumulation was analyzed on the transcriptome level in apple calli. The GO enrichment and KEGG analysis as well as anthocyanin metabolism pathway of DEGs demonstrated that ALA-induced expression changes of anthocyanin biosynthesis and transport structural genes may be the key reason for anthocyanin accumulation. The MYB family members were prominently involved in the transcriptional regulation of ALA-induced coloration. Two R2R3-MYB members, *MdMYB10* and *MdMYB9*, are likely involved in the regulation of ALA-mediated anthocyanin biosynthesis via calli transient expression. Additionally, *MATE* gene family members’ identification, phylogenetic trees, expression pattern, expression level analysis, *cis*-acting elements’ prediction, and Y1H screening revealed that MdMYB10 and MdMYB9 may activate *MdMATE8* transcription to regulate anthocyanin transport under ALA treatment. Overall, the study disclosed the key regulators and the putative mechanism behind the positive transcriptional regulation of ALA on anthocyanin accumulation in apple.

## Data Availability Statement

The datasets presented in this study can be found in online repositories. The names of the repository/repositories and accession number(s) can be found below: https://www.ncbi.nlm.nih.gov/, PRJNA525304.

## Author Contributions

JZ, YA, and LW conceived and designed the experiments. JZ, LL, HT, and YA performed the experiments. JZ, LL, HT, and YA analyzed the data. JZ, YA, and LW drafted and modified the manuscript. All authors read and approved the manuscript.

## Conflict of Interest

The authors declare that the research was conducted in the absence of any commercial or financial relationships that could be construed as a potential conflict of interest.

## Footnotes

1 http://www.Bioconductor.org

2 http://www.megasoftware.net

3 http://bioinformatics.psb.ugent.be/webtools/plantcare/html/
