# Supplementary material for: Transcriptomic Profiling of Apple Calli With a Focus on the Key Genes for ALA-Induced Anthocyanin Accumulation
Source: Front Plant Sci. 2021 Mar 26;12:640606. doi: 10.3389/fpls.2021.640606 (PMC8033201; doi:10.3389/fpls.2021.640606)
Supplement: Supplementary file 1 [file Data_Sheet_1.docx]

Supplementary Material

**Supplementary Table S1 MYB genes used to build phylogenetic tree**

|  | | |
| --- | --- | --- |
| Gene name | GenBank accession numbers | Protein sequence |
| NtAN2 | ACO52470 | MNICTNKSSSGVKKGAWTEEEDVLLKKCIEKYGEGKWHQVPLRAGLNRCRKSCRLRWLNYLRPHIKRGDFSFDEVDLILRLHKLLGNRWSLIAGRLPGRTANDVKNYWNSHLRKKLIAPHDQKESKQKAKKITIFRPRPRTFSKTNTCVKSNTNTVDKDIEGSSEIIRFNDNLKPTTEELTDDGIQWWADLLANNYNNNGIEEADNSSPTLLHEEMPLLS |
| PhAn2 | AAF66727 | MSTSNASTSGVRKGAWTEEEDLLLRECIDKYGEGKWHLVPVRAGLNRCRKSCRLRWLNYLRPHIKRGDFSLDEVDLILRLHKLLGNRWSLIAGRLPGRTANDVKNYWNTHLRKKLIAPHDQKQESKNKAVKITENNIIKPRPRTFSRPAMNNFPCWNGKSCNKNTIDKNEGDTEIIKFSDEKQKPEESIDDGLQWWANLLANNIEIEELVSCNSPTLLHEETAPSVNAESSLTQGGGSGLSDFSVDIDDIWDLVS |
| LeANT1 | AAQ55181 | MNSTSMSSLGVRKGSWTDEEDFLLRKCIDKYGEGKWHLVPIRAGLNRCRKSCRLRWLNYLRPHIKRGDFEQDEVDLILRLHKLLGNRWSLIAGRLPGRTANDVKNYWNTNLLRKLNTTKIVPREKINNKCGEISTKIEIIKPQRRKYFSSTMKNVTNNNVILDEEEHCKEIISEKQTPDASMDNVDPWWINLLENCNDDIEEDEEVVINYEKTLTSLLHEEISPPLNIGEGNSMQQGQISHENWGEFSLNLPPMQ |
| IbMYB1 | BAF45114 | MVISSVWSGSSSRVRKGSWSEEEDQLLRECIQKYGEGKWHLIPLRAGLNRCRKSCRLRWLNYLRPDIKRGEFSPDEIDLILRLHRLLGNRWSLIAGRIPGRTANDVKNLWNTHLQKKVSAMASSRQDNYWKGKAPEITENTVVRPRPRRFLKASSSPTTLLTGNATMVAYDGQLQEHMTTQPETTSDLLMENVQQKNLTTTLPSALETTPHDNVKWWEDVLSDKELNEEGQICWSEFPTDIDLLSELLS |
| InMYB2 | BAE94709 | MVNSSSAWPPPSSSRLMRKGAWTEEEDNLLRKCIQKYGEGKWHLVPLRAGLNRCRKSCRLRWLNYLRPDIKRGDFSVDEVDLIMRLHRLLGNRWSLIAGRIPGRTANDVKNYWNTHIQKKVFAMARMQDNWKGKAPEIRENTVVRPRPRRFLNTSLSPTSKTGKATAVTYDAQIQGHTLPQPPEAIITTSDLVMENVQLNNTIATLPSELETTTSDDRVRWWEDLLFDKEFNDDEGNACMHEGQVGWTNLPIDMD |
| VvMYBA1 | BAD18977 | MESLGVRKGAWIQEEDVLLRKCIEKYGEGKWHLVPLRAGLNRCRKSCRLRWLNYLKPDIKRGEFALDEVDLMIRLHNLLGNRWSLIAGRLPGRTANDVKNYWHSHHFKKEVQFQEEGRDKPQTHSKTKAIKPHPHKFSKALPRFELKTTAVDTFDTQVSTSRKPSSTSPQPNDDIIWWESLLAEHAQMDQETDFSASGEMLIASLRTEETATQKKGPMDGMIEQIQGGEGDFPFDVGFWDTPNTQVNHLI |
| VvMYBA2 | BAD18978 | MKSLGVRKGAWTQEEDVLLRKCIEKYGEGKWHLVPLRAGLNRCLKSCRLRWLNYLKPDIKRGEFALDEVDLMIRLHNLLGNRWSLIAGRLPGRTANDVKNYWHGHHLKKKVQFQEEGRKKPQTHSKTKAIKPHPHKFSKALPRFELKTTAVDTFDTQVSTSSKPSSTSPQPNDDIIWWESLLAEHAQMDQETDFSASGEMLIASLWTEETATQKKGTHSKTKAIKPHPHKFSKALPRFELKTTAVDTFDTQVSTS |
| CsRuby | AFB73913 | MADSLGVRKGAWTGEEDDLLRKCIEKYGEAKWHQVPLRAGLHRCRKSCRLRWLNYLNPNIKRGEFAADEVDLILRLHKLLGNRWSLIVGRLPGRTANDVKNFWNTHLRKKVDKCCKNNKEMKAKAEKVEKINIIKPQPRTFAKNSQWLKGKGMTSNNLQLGDYNLGKQSTPSDHHHHHQQQQENETESVWWESFLFGDELDQQGISSSLSRPEEESTTANIFAEKSPVVTKVTENRVIEAGQSCPTDDFAFDAEL |
| AmVENOSA | ABB83828 | MGNNPLGVRKGTWTKEEDILLKQCIEKYGEGKWHQVPIRAGLNRCRKSCRMRWLNYLSPNIKRGSFTRDEVDLIVRLHKLLGNRWSLIAGRLPGRTGNDVKNFWNTHFEKKSGERENTENINPKLINSSNIIKPQPRTFLKLRPKETKKQKNIRNVCTANDDKQQPLSTSGQLEEVNERIRWWSELLDFADYVD |
| AmROSEA1 | ABB83826 | MEKNCRGVRKGTWTKEEDTLLRQCIEEYGEGKWHQVPHRAGLNRCRKSCRLRWLNYLRPNIKRGRFSRDEVDLIVRLHKLLGNKWSLIAGRIPGRTANDVKNFWNTHVGKNLGEDGERCRKNVMNTKTIKLTNIVRPRARTFTGLHVTWPREVGKTDEFSNVRLTTDEIPDCEKQTQFYNDVASPQDEVEDCIQWWSKLLETTEDGELGNLFEEAQQIGN |
| AmROSEA2 | ABB83827 | MQKNPRGVRKGTWTKEEDILLMECIDKYGEGKWHQVPLKAGLNRCRKSCRLRWLNYLRPNIKRGCFSKDEVDLIVRLHKLLGNKWSLIAGRIPGRTANDVKNFWNTHVGKNLGVDGERRKKNVMNTKNSKETNIIRPRARTFNGLHVTWPREHGKNDAFSNVRITSTTENLDYEKQKPFHNNVASTPEEVDESIRWWSNLLETTEDELENLFEDVQQTGKMSEW |
| AtPAP2/MYB90 | AAG42002 | MEGSSKGLRKGAWTAEEDSLLRLCIDKYGEGKWHQVPLRAGLNRCRKSCRLRWLNYLKPSIKRGRLSNDEVDLLLRLHKLLGNRWSLIAGRLPGRTANDVKNYWNTHLSKKHESSCCKSKMKKKNIISPPTTPVQKIGVFKPRPRSFSVNNGCSHLNGLPEVDLIPSCLGLKKNNVCENSITCNKDDEKDDFVNNLMNGDNMWLENLLGENQEADAIVPEATTAEHGATLAFDVEQLWSLFDGETVELD |
| AtMYB114 | Q9FNV8 | MEGSSKGLRKGAWTAEEDSLLRQCIGKYGEGKWHQVPLRAGLNRCRKSCRLRWLNYLKPSIKRGKFSSDEVDLLLRLHKLLGNRWSLIAGRLPGRTANDVKNYWNTHLSKKHEPCCKTKIKRINIITPPNTPAQKVDIF |
| AtPAP1/MYB75 | AAG42001 | MEGSSKGLRKGAWTTEEDSLLRQCINKYGEGKWHQVPVRAGLNRCRKSCRLRWLNYLKPSIKRGKLSSDEVDLLLRLHRLLGNRWSLIAGRLPGRTANDVKNYWNTHLSKKHEPCCKIKMKKRDITPIPTTPALKNNVYKPRPRSFTVNNDCNHLNAPPKVDVNPPCLGLNINNVCDNSIIYNKDKKKDQLVNNLIDGDNMWLEKFLEESQEVDILVPEATTTEKGDTLAFDVDQLWSLFDGETVKFD |
| AtMYB113 | Q9FNV9 | MGESPKGLRKGTWTTEEDILLRQCIDKYGEGKWHRVPLRTGLNRCRKSCRLRWLNYLKPSIKRGKLCSDEVDLVLRLHKLLGNRWSLIAGRLPGRTANDVKNYWNTHLSKKHDERCCKTKMINKNITSHPTSSAQKIDVLKPRPRSFSDKNSCNDVNILPKVDVVPLHLGLNNNYVCESSITCNKDEQKDKLININLLDGDNMWWESLLEADVLGPEATETAKGVTLPLDFEQIWARFDEETLELN |
| GmMYB10 | ACM62751 | MERSSGIRKGTWTVEEDKLLRMCVEKYGEGKWHQIPKKAGLNRCRKSCRLRWLNYLKPNIKRGDFLADEVDLMLKLHKLLGNRWSLIAGRLPGRTANDVKNFWNTHLKKRTVSPPEDEENLKSPTPQKIVTRGNIFKPRPRKFSNCSCPFDASRKKSDIGINSLQSYQLSNNSKSVISLQNHPLVPPISTEENPAWWETMLFEENLEENKLDTKANGWCEQDDQFLTSFFNGEITQGTTVEGSTKNDESGHWPDL |
| MrMYB1 | ADG21957 | MEGSLGVRKGAWTVEEDTLLKLYIEKYGEGKWHQVPPRAGLNRCRKSCRLRWLNYLKPNIKRGEFKADEVDLMIRLHKLLGNRWSMIAGRLPGRTANDVKNYWNTHLRKNAISRIKDGGEKAQQTSKVNIIKPRPRTFAKNLTWFGGKPTIMAASFQPKDNVISDLPPAPLPSENSVKWGENLFDDKEAGDEIGTYDVGGLNEEPIATFRWAEAAPAETVGTPLDEFGPSFWAEFPSNLDVWDFLDP |
| GhMYB10 | CAD87010 | MGAEARSGLRKGAWTAEEDMLLKNCIERYGEGKWHLVPLKAGLNRCRKSCRLRWLNYLRPNIKRGDFGEDEIDLIIRLHKLLGNRWSLIAGRIPGRTANDVKNWWNTHLRSRHQQQQKVHQEDELSQDTTVAIIKPQPRTFSKTLNWFGNRQSVKDHVDINIIKSSSASDTNNISAPPELIASPKILDDAINECRQKLFDGDEKEVDIDGHVRWSFTPADEEPLNIVDQENGHDSLLDFPIDEVVWDLLN |
| LhMYB6 | BAJ05399 | MSPFRVSATSSSFSQMSPSPVLRLVRKGAWTQVEDDLLKRCIERHGVVRWSRVPQLAGLNRCRKSCRLRWLNYLDPRIRRGQFEEDEDDLIIRLHKLLGNRWSLIAGRLPGRTANDVKNYWNSHLSKKLIPQEKKVRACPCIAAPTRPQPRKCSIKTKTSVDDQQVNMSELIPQKKKVRACRIIAAPTRPQPRKCSIETKTSVDEQQVNMSESRPSADTANCAVWQDDLGNVKEMIEQLTEATIPSENTEGFAHE |
| EsMYBA1 | AGT39060 | MKPDFSEMFKSGVRKGAWTKEEDEVLKICVEKYGVGNWHRIPQRAGLNRCRKSCRMRWLNYLNPNINRGVSREDEIDLMLKMHKLLGNRWSLIAGRLPGRTANDVKNFWNTQLRHKSVLNNKDKERILPPKKVQVIKPHPRIFKPVPTRLTGEPAFCNLQEQQQEEGNQHPIAEDTIWWEELLFHDKEMNHGTSVSFGREEVVSTTNSTEEERKAALFSDVDFEFQDFSDLNFWNFE |
| ZmC1 | AAA33482 | MGRRACCAKEGVKRGAWTSKEDDALAAYVKAHGEGKWREVPQKAGLRRCGKSCRLRWLNYLRPNIRRGNISYDEEDLIIRLHRLLGNRWSLIAGRLPGRTDNEIKNYWNSTLGRRAGAGAGAGGSWVVVAPDTGSHATPAATSGACETGQNSAAHRADPDSAGTTTTSAAAVWAPKAVRCTGGLFFFHRDTTPAHAGETATPMAGGGGGGGGEAGSSDDCSSAASVSLRVGSHDEPCFSGDGDGDWMDDVRALAS |
| ZmPl | AAA19819 | MGRRACCAKEGVKRGAWTAKEDDTLAAYVKAHGEGKWREVPQKAGLRRCGKSCRLRWLNYLRPNIKRGNISYDEEDLIVRLHKLLGNRWSLIAGRLPGRTDNEIKNYWNSTLGRRAGGSRVVFAPDTGSHATPAAGSREMTGGQKGAAPRADLGSPASAAVVWAPKAARCTGGLFFHRRDTHTPHAGGTETPTPMMAGGGGGEARSSDDCSSAASVSPLVGSSQHDPCFSGDGDGDWMDDVRALASFLESDEERL |
| LjTT2a | BAG12893 | MGRSPCCSKQGLNRGAWTAQEDQILRDYVHLHGQGKWRNLPQSAGLKRCGKSCRLRWLNYLRPDIKRGNISRDEEELIIRLHKLLGNRWSLIAGRLPGRTDNEIKNYWNTNLCKRVQDGVDVGDSKTPSSQEKNNHHDQKAKPQSVTPSVFSSSQPKNNNVIRTKASKCSKVLLRDPLLPCPPMQTQSDDFIAKLLEEAEGEPLLSAVANDFTSGDEDGVLSFDPCGNEKELSTDLLLDLDIGEICLPEFINSDF |
| VvMYBPA2 | ACK56131 | MGRRPCCAKEGLNRGSWSAWEDKILCNYVEVHGEGKWRDLPQRAGLKRCGKSCRLRWLNYLRPDIKRGNISSEEEELIIRLHKLLGNRWSLIAGRLPGRTDNEIKNYWNTNLSKRLQASKGQNSPNKKVENPKNQTSGTGKSSAELHTVIRTRAVRCSKVIIPRVQADFDENPSPKMAVPTSEPSSSALEQGETANFFMGFDIGDLLTSDALNSFLDQDEEMGENNSNGVSDHFPPCSDFLAPEIENQEGVSGLL |
| FaMYB11 | AFL02461 | MGRSPCCAKEGLNRGAWTAMEDRTLTEYITTHGEGKWRNLPKRAGLKRCGKSCRLRWLNYLRPDIKRGNITRDEEELIIRLHKLLGNRWSLIAGRLPGRTDNEIKNYWNTNIRKKVQDHSSTNSEANITHHKPPNHQTQKKNTNVVRTKASRCTKVFMPHQQSQMDKKGTCNNPTADQQGAAPFLNHDYYDPINYNDDPALRMMGITDTHHQESDDLSPFLNLEIDNENSNSCGFMVDFKMDESFLSEFLNVDFS |
| AtTT2 | NP_198405 | MGKRATTSVRREELNRGAWTDHEDKILRDYITTHGEGKWSTLPNQAGLKRCGKSCRLRWKNYLRPGIKRGNISSDEEELIIRLHNLLGNRWSLIAGRLPGRTDNEIKNHWNSNLRKRLPKTQTKQPKRIKHSTNNENNVCVIRTKAIRCSKTLLFSDLSLQKKSSTSPLPLKEQEMDQGGSSLMGDLEFDFDRIHSEFHFPDLMDFDGLDCGNVTSLVSSNEILGELVPAQGNLDLNRPFTSCHHRGDDEDWLRD |
| FaMYB9 | AFL02460 | MGRSPCCSKEGLNRGAWTALEDKVLTSYIKAHGEGKWRNLPKRAGLKRCGKSCRLRWLNYLRPDIKRGNISGDEEELIIRLHNLLGNRWSLIAGRLPGRTDNEIKNYWNTTLSKKAKPESHSGSSKETSPGPTRFRPRKASAAATTQPQVIRTKATRLTRMPVPSLPLLIDDCSTSTTALELQVPQTQLVSSLPEDAVNTQVHFQGTDAMNFGCNGFQATAGDDEDAKGDYDIPLDDGMLNDWTGNGNCDLENYG |
| OsMYB3 | BAA23339 | MGRKPCCSKEGLNRGAWTAMEDDILVSYIAKHGEGKWGALPKRAGLKRCGKSCRLRWLNYLRPGIKRGNISGDEEELILRLHTLLGNRWSLIAGRLPGRTDNEIKNYWNSTLSKRVAMQRTAAATSMPAAATTSSNADAAGAAARRRRSPEPRTVVVSPIRTKALRCNNNSSSGIVVVQQAGACSHGGRPPESGAPGDAAADKVATPQAVQQQQQQELAGAEDDDDLPVPAVCIDLDLDDIELGGLDGFLISPWR |
| DkMYB4 | BAI49721 | MGRAPCCSKVGLHRGPWTGKEDGLLTKYIQVHGEGSWRSLPKKAGLLRCGKSCRLRWMNYLRPDIKRGNITPDEDDLIIKMHALLGNRWSLIAGRLPGRTDNEIKNYWNTHLSKRLRSQGTDPNTHKKLSDSHVQEPKKRSSNKKQKNKSKSNLDHTEKLKVHNPKPFRIKSLASFSFSRDSSSFDWTTTTATATPSGSSNHEGERGMLGNNGSNGHEVGFFIGEDGSDHYHHDHMMDDSDLECQSLEKLYEEYL |
| VvMYBPA1 | CAJ90831 | MGRAPCCSKVGLHRGSWTAREDTLLTKYIQAHGEGHWRSLPKKAGLLRCGKSCRLRWMNYLRPDIKRGNITPDEDDLIIRLHSLLGNRWSLIAGRLPGRTDNEIKNYWNTHLSKKLRSQGTDPNTHKKMTEPPEPKRRKNTRTRTNNGGGSKRVKISKDQENSNHKVHLPKPVRVTSLISMSRNNSFESNTVSGGSGSSSGGNGETLPWPSFRDIRDDKVIGVDGVDFFIGDDQGQDLVASSDPESQSHMPPTDN |
| AtMYB32 | EFH43356 | MGRSPCCEKDHTNKGAWTKEEDDKLISYIKSHGEGCWRSLPRSAGLQRCGKSCRLRWINYLRPDLKRGNFTLEEDDLIIKLHSLLGNKWSLIATRLPGRTDNEIKNYWNTHVKRKLLRRGIDPATHRPINKTPQDSSDSSKTEDSLVKILSFGPQLEKIANFGDERNEKEVMCQKERVEYSVVEERCLDLNLELRISPPWQDQLHDEKNLRFGRVKRMCTACRFGFGNGKECSCDNTKSQTEDSSSSSYSSTDFS |
| AtMYB4 | NP_195574 | MGRSPCCEKAHTNKGAWTKEEDERLVAYIKAHGEGCWRSLPKAAGLLRCGKSCRLRWINYLRPDLKRGNFTEEEDELIIKLHSLLGNKWSLIAGRLPGRTDNEIKNYWNTHIRRKLINRGIDPTSHRPIQESSASQDSKPTQLEPVTSNTINISFTSAPKVETFHESISFPGKSEKISMLTFKEEKDECPVQEKFPDLNLELRISLPDDVDRLQGHGKSTTPRCFKCSLGMINGMECRCGRMRCDVVGGSSKGSD |
| AtMYB7 | NP_179263 | MGRSPCCEKEHMNKGAWTKEEDERLVSYIKSHGEGCWRSLPRAAGLLRCGKSCRLRWINYLRPDLKRGNFTHDEDELIIKLHSLLGNKWSLIAARLPGRTDNEIKNYWNTHIKRKLLSKGIDPATHRGINEAKISDLKKTKDQIVKDVSFVTKFEETDKSGDQKQNKYIRNGLVCKEERVVVEEKIGPDLNLELRISPPWQNQREISTCTASRFYMENDMECSSETVKCQTENSSSISYSSIDISSSNVGYDFLG |
| AtMYB12 | ABB03913 | MGRAPCCEKVGIKRGRWTAEEDQILSNYIQSNGEGSWRSLPKNAGLKRCGKSCRLRWINYLRSDLKRGNITPEEEELVVKLHSTLGNRWSLIAGHLPGRTDNEIKNYWNSHLSRKLHNFIRKPSISQDVSAVIMTNASSAPPPPQAKRRLGRTSRSAMKPKIHRTKTRKTKKTSAPPEPNADVAGADKEALMVESSGAEAELGRPCDYYGDDCNKNLMSINGDNGVLTFDDDIIDLLLDESDPGHLYTNTTCGVM |
| SlMYB12 | ACB46530 | MGRTPCCEKVGIKRGRWTAEEDQILTNYIISNGEGSWRSLPKNAGLLRCGKSCRLRWINYLRSDLKRGNITSQEEDIIIKLHATLGNRWSLIAEHLSGRTDNEIKNYWNSHLSRKVDSLRIPSDEKLPKAVVDLAKKGIPKPIKKSSISRPKNKKSNLLEKEALCCTNMPACDSAMELMQEDLAKIEVPNSWAGPIEAKGSLSSDSDIEWPRLEEIMPDVVIDDEDKNTNFILNCFREEVTSNNVGNSYSCIEEG |
| VvMYBF1 | ACV81697 | MGRAPCCEKVGLKKGRWTAEEDEVLVKYIQANGEGSWRSLPKNAGLLRCGKSCRLRWINYLRADLKRGNFSEEEEEIIIKLHASLGNRWSMIAGQLPGRTDNEIKNYWNSHLSRKVHSFRRLTNEGPSMVIDLAKVTTAHKRKVGRTSRWAMKKNRSDKSIREDVNKSSLEKPKGDDDGNGVIAEKETRSETMTGDLYAQVNEEENPELMASHLLGCGGRMFGEGSETFGPFRVPEVEGLCFSENMESGVLVDGG |
| VvMYB5a | AAS68190 | MRNPASASTSKTPCCTKVGLKRGPWTPEEDELLANYVKREGEGRWRTLPKRAGLLRCGKSCRLRWMNYLRPSVKRGQIAPDEEDLILRLHRLLGNRWSLIAGRIPGRTDNEIKNYWNTHLSKKLISQGIDPRTHKPLNPKPNPSPDVNAPVSKSIPNANPNPSSSRVGEIGSNHEVKEIESNENHKEPPNLDQYHSPLAADSNENWQSADGLVTGLQSTHGTSNDDEDDIGFCNDDTFPSFLNSLINEDVFGNHN |
| VvMYB5b | AAX51291 | MRNASSASAPPSSSSKTPCCIKVGLKRGPWTPEEDEVLANYIKKEGEGRWRTLPKRAGLLRCGKSCRLRWMNYLRPSVKRGQIAPDEEDLILRLHRLLGNRWALIAGRIPGRTDNEIKNYWNTHLSKKLISQGIDPRTHKPLNPNSSSVDVKASSSKAKAVMNPNPNPNPSPSEKAAANKEAGNFKSDNQYQIGAAGNDGSANIQNSDGSGTGLRSSNNEEDDDLNCGTDDVFSSFLNSLINEDVFPGQHHLQQQ |
| DcMYB6 | ARD08871 | MHPKALKNSTNPLKLRKGAWGSDEDALLRKCIEKYGEGKWHLVPRRAGLNRCRKSCRLRWLNYLRPTIKRGDFAADEVDLMMRLHKLLGNRWSLIAGRLPGRTANDVKNFWNTNVQKKLTTSSNHGQTEAVKVQEVVNKNQTSNAGTSAAATHVVVKPLPRTLSKGTSVPCYNPNAIGHKHSPWPGGMVYNKISSSNNNNNSCMVMNKTLSPAAPLPDQDGTEWWKNLFAEIGIQGQEEGSLEGHLVASSSGSEN |
| MDP0000031172 |  | MGRSPCCSKEGLNRGAWTALEDKILTAYIKAHGEGKWRSLPKRAGLKRCGKSCRLRWLNYLRPDIKRGNISGDEEELIVRLHNLLGNRWSLIAGRLPGRTDNEIKNYWNTTLWKKSKADSPSGSSKETSQHPSKSVVKKKDVESKTTSTAAAKPLVIRTKATRLSKILVPQNIPS |
| MDP0000127691 |  | MEGYNENLSVRKGAWTREEDNLLRQCVEIHGEGKWNQVSYKAGLNRCRKSCRQRWLNYLKPNIKRGDFKEDEVDLIIRLHRLLGNRY |
| MDP0000133416 |  | MAGGQHRGWGMINDEGWRKGPWTAEEDGFLIEHVRFHGEGRWNSVARLAGLKRNGKSCRLRWVNYLRPEHKRGQITPHEESLILDLHARWGNRTHFKKEAKMPSDASERAKNHILRRQKFHNQQQQQKNLQVDEEEVKRIMSLLDENETKMPLYWPHAANLR |
| MDP0000159011 |  | MRKPCCEKEGTNKGAWSKQEDQKLIDYIKTHGEGCWRSLPKAAGLHRCGKSCRLRWINYLRPDIKRGNFEQDEEELIIKLHALLGNRWSLIAGRLPGRTDNEVKNYWNSHIRKKLIKMGIDPNNHRLNQIIPRPNPQNDSVSPAATSSGSMSNINACTKTPLKSSDDQIDHRASEAASVLEDETSGPSSRDLNLDLTIAFPEPSLQVEEGMPKLIKGSNTTAREIETNLQHLPTLVLFR |
| MDP0000167107 |  | MSTNTKTLSSNYSGEDDSELRRGQWILEEDSLLIQYIERHGEGQWNLLAKRSGLRRTGKSCRLRWLNYLKPDVKRGNLSPEEQLLILDLHSKMGNRWSKIARYLPGRTDNEIKNYWRTRVHKQARHLNIDTKSREFQNMIRCYWMPRLKQKIGRETSISSAVLNQNPTISQPRENNTFQHFTATISPPPQILVQEEINMSGTMYNLDVEKQNTEADYCRSSFIFPSEPMDMSKTAQFPECPPFYCGDNNGYDMES |
| MDP0000175918 |  | MGRAPCCEKAHTNKGAWTKEEDQRLIDHIRQHGEGCWRSLPKAAGLLRCGKSCRLRWINYLRPDLKRGNFTQEEDELIIKLHSLLGNKWSLIAGRLPGRTDNEIKNYWNTHIKRKLISRGLDPQTHRPLNQTTTAAAAATPASRLDLRNRSSPSSAVFDHKTIKNNKFELLKHPKMEHEYYNYNIESEANCSTTTGSGTTTDEDQKQQNKYKCSDLNLDLSIGLEPFQSEPTRASSGNSAESRLQRIIAPSNSNN |
| MDP0000197283 |  | MGRAPCCDKNGLKKGPWTPEEDQKLMDYIQKHGYGNWRTLPKNAGLQRCGKSCRLRXTNYLRPDIKRGXFSFEEEETIIQLHSILGNKWSAIAARLPGRTDNEIKNYWNTHIRKRLLRMGIDPVTHSPRLDLLDFSSILYNSSHHHHQMNNFSRLLGQPIGLNPELLRLATSLIQSRXENNSNQNFVLQNAQENDYHQICNPQIQPQQPVQDNVPYPNEVSQLMQQQPNVEYPSSLSDFRSQNSQLNEWQSNVGT |
| MDP0000210851 |  | MGRSPCCSKEGLNRGAWTALEDKILSSYIKAHGEGKWRSLPKRAGLKRCGKSCRLRWLNYLRPDIKRGNISGDEEELIVRLHNLLGNRWSLIAGRLPGRTDNEIKNYWNTTLGKKSKVDSFSGSSKETSLNPCKSIAKKKDVESKTSTAAAQPLVIRTKATRLTKILVPQNIPSDENYTAAAANPLELQTQSAEKGGSTEEFPRTNAGDCSNILKNFGCDDDDIDAKGDQYCNEFQLLNSIPLDEAXINDGCWTG |
| MDP0000241185 |  | MSSSSVWNKEEDKEFENAIAMHWIDENSKEMWEKIAELVPSKSMGELKQHYQMLVDDVGAIEAGRVSPPNYAVDEAANTLSSSKDSGHRASSSGASASDKRLNCGHGGGFSGLGHDSAGHGGKGGSRADQERKKGIPWTEEEHRLFLLGLDKFGKGDWRSISRNFVISRTPTQVASHAQKYFIRLNSMNRDRRRSSIHDITSVNNGDVSSHQQPPITGQQTNTYPPSAGTAIRVGGPQTAKHRPQSHMAGLGMYG |
| MDP0000259614 |  | MEGYNENLSVRKGAWTREEDNLLRQCRKSCRQRWLNYLKPNIKRGDFKEDEVDLIIRLHRLLGNRWSLIARRLPGRTANAVKNYWNTRLRIDSRMKTVKNKSQEMRKTNVIRPQPQKFNRSSYYLSSKEPILDHIQSAEDLSTPPQTSSSTKNGNDWWETLLEGEDTFEXAAYPSIELEEELFTSFWFDDRLSPRSCANFPEGQSRSEFSFSTDLWNHSKEE |
| MDP0000261265 |  | MVPVSSRSSKKDVNRGSWTAEEDQKLAQVIEIHGPRRWKSIATKAGLKRCGKSCRLRWMNYLRPNIKRGNISDQEEDLILRLHKLLGNRWSLIAGRLPGRTDNEIKNYWNSHLSKKMKQNRAVSKTVQGSTEQKNIKANDVNALTIEREDAFKLEENFNFGFNGGQFFNCSSSEQGPLNLEWMNKFLEMDESWFTLHDI |
| MDP0000266156 |  | MGRTPCCDKENVKRGPWSPEEDAALKSYLQSHGSDGTASNWIHLPKKAGLRRCGKSCRLRWLNYLRPDIKHGGFTEEEDSIICNLYNQMGSRQYFLTYLHFLNSPNFLRWSVIASYMPGRTDNDVKNYWNTKLKKKLLGGKIKNISSKEPTIANNANFFGIPEAEKPQDSAFSTSEPQVPSTLQMLYDVXSGLSADNQTMSLNPDQLYNPKLSGFSDLGARSRRNYSTTVSLSQEGSSISDSSSMAGNLYLDQDS |
| MDP0000268980 |  | MQEETREIPKPKTSKDDSSKPIPESEDTFLXQRHIXNPIEXSLAEPGLPAKRCHRRXKFFTELNGFPSLAKNHRTDLNKDVDVXSLIAISKGFLVNSLTXXEIEANVVPTIGXVXQANYIVGKNHILSRWRSNVSFWLTRELALESIRSKHKGLVXSAYEFXVEHGYINFGLAPTGNHSGISGTSCHHNSMGLNLNLSSNFGRQPPQSQAAPEYRETEVHRLQRXVFFLAVEQEVLQLQILMHNPELNDLSPASS |
| MDP0000298689 |  | MGAMSSEDRVTGSWNPQEDATLIKLVAQHGPSNWSLISTGIPGRSGKSCRLRWCNQLCPTVQHKPFSPQEDSIIIQAHALHGNKWATIARLLPGRTDNAIKNHWNSTLRRWRRQLAKLSSESSCSDSAAAHGEEPDLKRQCLRASPEHESLKADAGGEGVIVKTSLTLSPPGENAENDVAVKSEEEEHDREAVDNNDDGEKCAVEMDETCLLTIMQRMIAQEVRNYIYGLRPDGGPTFGLHSAHQNENDAQQNME |
| MDP0000317257 |  | MEGCNVNLSVXRKGAWTREEDDLLRQCIEIHGEGKWRQLPNKAGLNTCRKSCRLRWLNYLKPNIKRGDFTEDEVDLTIRLHKLLGNRY |
| MDP0000407613 |  | MVRTPCRDENGMKKGTWTPDEDRKLIAYVTRYGCWNWRQLPKFAGLSRCGKSCRLRWMNYLRPNIKRGNYSTEEEETIVKLHEKLGNRWSAIAAQLPGRTDNEIKNHWHTNLKKRTNNKQCNSSFSSSATNTEETPSYSSLEAAVDQPIKKAIFPNAESTVTPQVTQKTDDRVDNSSQLSPSPQPSSSEVSSMSADNNWVNYVEDINVTSMEAYADSQFIDDFWTEPFLADNSYIPSGFYTPLMDSEFVYPLFGG |
| MDP0000477900 |  | MEALXICSSSASSSDTSSSESSLTRNPNKPERIKGPWSAEEDRVLTRLVERYGPRNWSLISRYIKGRSGKSCRLRWCNQLSPSVQHRSFSQAEDETILAAQARFGNRWATIARLLPGRTDNAVKNHWNSTLKRRVRGDQLTEGGSFLGGGGNVGSNEGMSTNSVSGSLVNGSMEFDPLTELTLAPPGIGSGSGGAMVAEQRRNNESVPAGFWDAMRDVIAREVRDYVATTFSEPSGLL |
| MDP0000573302 |  | MEGYNVNLSVMRKGAWTREEDDLLRRCIETLGEGKWHQVPYKAVTSAYVKRAMPRDLVPHIIVHVERCXXNLGLNRCRKSCRLRWLNYLKPNIKRGDFTEDEVDLIVRLHKLLGNRWSLIAGRLPGRTSNDVKNYWNTQLRIDSGVKTMKNKFQETRKTIAIRPQPQKFIQGSYYLNSKEPILDHIQAAEDLSTLPQTSSSTKNGNDWWXTLLEDEDAFERIACPSLELEEEHFS |
| MDP0000655330 |  | MRNPSSSSKAAAAASAKMQTTITASSSSSKAAGVAGGTKTPCCAKVGLKRGPWTPEEDELLANYIKKEGEGRWRTLPKRAGLLRCGKSCRLRWMNYLRXSVKRGQIAPDEEDLILRLHRLLGNRWSLIAGRIPGRTDNEIKNYWNTHLSKKLISQGIDPRTHKPLNXDHHSAADDXDVDNTNKSTAVASSSKANDRFSNPNPSPPSDRLVHKEGDPNNSRNDGNIAIADHDLGTIVXGFANMITSINNPDASSSA |
| MDP0000693678 |  | MAMKGAXSGEDRVKGSWSPQEDATLIKLVAQHGPRNWSLISTGIPGRSGKSCRLRWCNQLSPTVQHKPFSXQXDSVIIQAHALHGNKWATIARLLPGRTDNAIKNHWNSTLRRGRQRAEXSSTSSGSDLAAAVGDEPDMKRQCLRASPEHESLKANAGGEGVIVETSLTLSPPGEKAENEVVLKSEEEEDGHEAVDNNDDVENCRVETEETSLLKIMHRMIAREVRNYIDSLRENGGPTFGLQSAAHQNDP |
| MDP0000755899 |  | MAFRHLMSDCDGFTRYQDLNFLPPPPVSNQLSLSNIFGGSSTSAMGVVPFSPSSSQQTNKPWSFQALENGGPKNISDLGVLSGVDQKRPTPLNLNLVGEEDDDEGRSPAGGRTSAKSCIRGHWRPAEDSKLKELVGQYGPQNWNVIAEHLDGRSGKSCRLRWFNQLDPRINRGAFSEEEEERLLAAHRLYGNKWAMIARLFPGRTDNAVKNHWHVIMARRHREQSNVFKRRKPSSPPPPSPLPHVVANFPKNPST |
| MDP0000764760 |  | MPVQSKFCLISYSQELVDGQPLYASSNCLPVKALNREPAGHSFHAVALKLRGCVEENKEVEDEKVVNNKEQTSIPSFDSYSSKGKKKSGGEGKEQDHYALLGLSHLRYLATEEQIRKSYRETALKYHPDKQAALLLNEVTEAAKQTKKDEIESHFKSIQEAYEVLIDPVKRRIYDSTDEFDDEIPTECAPQDFFRVFSPAFMRNGRWSVSQPIPFLGDESTPLKEVDDFYDFWYTFKSWREFPHADEFDLEQAES |
| MDP0000787808 |  | MGRQPCCDKLGVKKGPWTAEEDKKLVNFLLTHGQCCWRAVPKLAGLRRCGKSCRLRWINYLRPDLKRGLLNDAEEQLVIDLHARLGNRWSKIAARLPGRTDNEIKNHWNTHIKKKLVKMGIDPITHEPLHKQVTTXQEMPCEASNQPANSDMSIQQMNTNIPEHGISTNSDGNSTSENSPSNDSEPAEPNPNYSEEEDPLVSFILSDTFLEDLTWDFSTSSEYSSADNPTEENSLAWFMDCNDFGVEDFEL |
| MDP0000819856 |  | MDGEKLRKGPWLEEEDERLTTVVNLKGNRRWDAXAKESGLRRSGRSCRMRWLNYLRPNIKHGQITIIEEKTILQLHQRWGNKWSKIARALPGRTDNEIKNYWRTHLKKKTQIPDGNFQCTLNKNGRGLFCQEGDMNNEKYDFDQDHDSVKNSWETKVTSSDDLGLSDFAVTNSPYETQLSDWISELSSEQNGTAYNQDCNSVESDLCHLTWTPDDSDTWDCPSFLWDMN |
| MDP0000887107 |  | MGRSPCCAKEGLNRGAWTAHEDKVLTQYIKLHGEGRWRNLPKKAGLKRCGKSCRLRWLNYLRPDIKRGNISPDEEELIIRLHKLLGNRWSLIAGRLPGRTDNEIKNYWNTNLGKKVPDRQQQRSASNLKHHKNGEPNSKKAKSMDMASPSSLVYRTKAVKCTQVFINPQPHKVLLGHDHQHCTEETNTVLMFDGKPAAMDDDHINRTLSFSSFSNINADQENSTSDFLVDFDMNEISIASLLNSDFPEINRDYLN |
| MDP0000915330 |  | MGRAPCCSKVGLHRGPWTPREDTLLTKYIEAHGEGHWRSLPKKAGLLRCGKSCRLRWMNYLRPDIKRGNITPDEDDLIIRLHSLLGNRWSLIAGRLPGRTDNEIKNYWNTHLSKRLRNEGTDPNTHKKLSEPIARENKRRKNQRSKSNNNKKEMVMTKDKNNKTAQHVEPQKPKVHLPKPTRFTSFLSLPRNDSFTSSTTVTTGSSSQDLNGGGGRGGGGGGFGVNTWCNNGGLVFCVGDEDQDHDPINSSADGG |
| MDP0000932804 |  | MGRHSCCYKQKLRKGLWSPEEDEKLLNYITKHGHGCWSSVPKLAGLQRCGKSCRLRWINYLRPDLKRGPFSQQEENLIVELHAVLGNRWSQIAAQLPGRTDNEIKNLWNSCIKKKLRQRGIDPNTHKLLSEVLNQNIDTETNNNNNINLSPTYKSNEKASEGSNELSLVEAVSXKHPPSASENRFNPVEVSSTSKLISSKSLTHEGSXSSCRPCDFVGYFSFPHHNNQNNYGSSSDMGLQAVNQNTTFSFLNQNP |

**Supplementary Table S2 Sequences of 21 MdMATE members of clade I and 24 different known MATEs**

| Gene ID | Protein sequence |
| --- | --- |
| VvAM1  [*Vitis vinifera*] | METPLLNSGAEEGYSGPDGDYQPLRSWREVRSMVWKETVKLWRVAGPLAFQILCQFGTNSMTSVFVGHIGNLQLSAVSISLSVIGTFSFGFMLGMGSALETLCGQAYGAGQVHLLGVYLQRSWIILLVTCVILSPVYVFATPILKVLGQEDAIADLAGQFTIETIPQLFSLAIIFPTQKFLQAQSKVNVQATIAFVALILHIGMLSVFIFVFGWGTTGAAIAYDISNWVIAVSQVVYAIGWCKEGWTGLTWSAFR |
| VvAM3  [*Vitis vinifera*] | METPLLKSGAERGYGGEGGDYPPLTTWREVRSMLWRETVKVWRVAGPLAFQILCQFGTNLVTTVFVGHIGNLELSAVSISVSVIGTFSFGFMLGMGSALETLCGQAYGAGQVQLLGVYLQRSWIILLVSCIILLPIYIFATPILKALGQEDEIADLAGQFTLETIPQLFSLAIIFPTQKFLQAQSKVNVQATICFVALILDIGMLAVFIFVFGWGTTGAAIAYDISSWVTAVAQVVYAISWCKEGWTGLTWSAFR |
| MtMATE2  [*Medicago truncatula*] | MDSHTPLLNTTAATSSSSELLELDGGDYLEVKGFKQARKVFAIETLRIWKIALPIVFNILCQYGVNSITNIFVGHLGDIQLSAISLINSVIGTFAFGFMLGMGSATETLCGQAFGAGQVHMLGVYMQRSWIILFVTSIILLPIYIFAAPILKLLGQQEDMADLAGSFALLVIPQFLSLSFNFPTQKFLQSQSKVNVIAWIGLVALIVHIGLLWLLIYVLDLGLTGAAIAFDVTSWGITLAQLVYVVIWCKDCWNG |
| SlMTP77  [*Solanum lycopersicum*] | METPLLNGYSGSGERNDLIGADGDYRPAKSTKDWWAIFCVETLKLWRIGGPIAFNIICQYGVNSLTNIFVGHLGNVELSAISIAQTVISTFSFGFMMGMGSALETLCGQAYGAGQVHMLGVYMQRSIIILLATCVFLLPIYLFTTPLLVLLGQETAIADLSGRYTMLLIPQLFSLAINFPTSKFLQAQSKVDVLAGIGFAAVLVHALFLWLFIYTLEWGTNGAAIAFDLTNWLTAMAQLAYVVGWCKDGWKGLSW |
| GhTT12  [*Gossypium hirsutum*] | MGSAAPEYQPLLLGLDSHSRIPDLSSVAIEEFLQHRPIALRWWPRLVAWESRLLWLLSGSSIVLSIFNYMLSFVTLMFTGHLGALELAGASIASVGIQGLAYGIMLGMASAVQTVCGQAYGAKQYSAMGIICQRAIILHLGAAVLLTFLYWFSGDVLQAIGQTESIAQQGQVFSRGLIPQIYAFAISCPMQRFLQAQNIVNPLAFMSIGIFLVHVLLTWLVVNVLGCGLLGAALTLSLSWWFLVVINGLYIVLSP |
| AtDTX35 (AtFFT)  [*Arabidopsis thaliana*] | MDPTAPLLTHGGEVEEDYAPARSWTDVKRVLSTESAKLWMIAAPVGFNIICQYGVSSVTNIFVGHIGEVELSAVSISLSVIGTFSFGFLLGMGSALETLCGQAYGAGQVNMLGVYMQRSWIILFVSYFFLLPIYIFATPVLRLLGQAEEIAVPAGQFTLLTIPQLFSLAFNFPTSKFLQAQSKVVAIAWIGFVALSLHVIMLWLFIIEFGWGTNGAALAFNITNWGTAIAQIVYVIGWCNEGWTGLSWLAFKEIW |
| MtMATE1  [*Medicago truncatula*] | MENQPFLVGLDSHSHTHIADLSSDAIEEFLEHRPIGLRWWLKLVAWESRLLWILSGASIVVYLCNFMLSFVTMMFCGHLGSLELAGASIASVGIQGLAYGIMLGMASAVQTVCGQAYGAKKHAAMCITLQRAIILHFGAAVILTFLYWFSGDFLKVIGQTESIAVQGQVFARGLIPQLYAFAFSCPMQRFLQAQNIVNPLAYMAVGVLLLHALLSWLVVVVLGYGLLGAALTLSFSWWILVFLNALYIIFSPKCK |
| AtTT12  [*Arabidopsis thaliana*] | MSSTETYEPLLTRLHSDSQITERSSPEIEEFLRRRGSTVTPRWWLKLAVWESKLLWTLSGASIVVSVLNYMLSFVTVMFTGHLGSLQLAGASIATVGIQGLAYGIMLGMASAVQTVCGQAYGARQYSSMGIICQRAMVLHLAAAVFLTFLYWYSGPILKTMGQSVAIAHEGQIFARGMIPQIYAFALACPMQRFLQAQNIVNPLAYMSLGVFLLHTLLTWLVTNVLDFGLLGAALILSFSWWLLVAVNGMYILMS |
| VvMATE1  [*Vitis vinifera*] | MASAAEDGEPLLLGHSSAGIHELSSSAVEELLLHKPVPGRWWPRLFGWESRLLWVLSGSAIVSSVFNYMLSFITLMFAGQLGALELAGASIASVGIQGLAYGLMLGMASAVQTVCGQAYGAKKYAAMGIICQRAIVLHLGAAILLTFLYWYSGAFLKAIGQSESIAVQGQIFARGLILQVYAFALSCPMQRFLQAQNIVNPLAYIAVGVTLLHILLTWLVVNVLDSGLLGIALTLSLSWWLLVFSIALYILLSPS |
| VvMATE2  [*Vitis vinifera*] | MGSEEYQPLLLGLNSHARIPDLSSFAVEEFLAHKPVAVRWWPRLFGWESRLLWLLSGSSIVASIFNYMLSFVTLMFTGHLGALELAGASIASVGIQGLAYGIMLGMASAVQTVCGQAYGAKKYKAMGIICQRAIILHLGAAVLLTFLYWFSGPFLRAIGQSDSISAQGQIFARGLILQLYAFAISCPMQRFLQAQNIVNPLAYMAVGVFFLHVLLTWLVVYVLDYGLLGAALTLSFSWWILVVVIALYILLSPSC |
| VcMATE2  [*Vaccinium corymbosum*] | MAAAEEYHPLLPGILQEESLASAEVEEILMQKPVAASRYIKLFGWESKLLWILSWASIVVSICNYMLSFVTLTFSGHLGALDLAGASIAMVGAQGLAYGIMLGMASAVQTVCGQAYGAKQYGAMGIICQRAIILHLGMAILLTFLYVYFGEVLKIIGQAEDIAEKGQIFARGMIPQLYAFSISCPLQRFLQAQNIANPLAYMSLSVFLVHIFLSWLVVYHFDFGLLGAALTLSLSWWILVVLQGLYILLSPSCKN |
| FaTT12-1  [*Fragaria x ananassa*] | MGSSEQYQPLLLGLDSHSRIPDLSSAVIEEFLEQSPVAVRWWPRLVAWESRLLWILSGSSILVSVFNYMLSFVTLMFCGHLSALELAGASIASVGIQGLAYGIMLGMASAVQTVCGQAYGAKHLSAMGIICQRAIVLHLGAAVLLTFLYWFSGPILVAMGQSESIAEQGQIFARGLIPQLYAFAINCPQQRFLQAQNIVNPLAYMSIGVFLVHTLLTWVVVYVVDYGLIGAALTLSFSWYLLVITNGIYILVSPN |
| DkMATE1  [*Diospyros kaki*] | MGSEEYQTLLLRLDSHLSLSSSGVEGLLSQSQPTSLNWYLRLAGWESRLLWQLSGASIAVSIFNYMLSFVTLTFTGHLGALELAGASIASVGIQGLAYGIMLGMASAVQTVCGQAYGAKRYGALGVICQRAVVLHVGAAVLLTFLYWFAGPILKAIGQSDSIADEGQVFARGLIPQLYAFSISCPLQRFLQAQNIVNPLAYMSVGVFLLHILITWLAVYVLNYGLIGAALTLSLSWWLLVILQSLYILLSPSCAQ |
| Nt-JAT1  [*Nicotiana tabacum*] | MVEELPQSLKEKKWQINWDAVSQELKKTSRFMAPMVAVTVFQYLLQVVSVMMVGHLGELALSSVAIATSLTNVTGFSLLTGLVGGMETLCGQAYGAQQYHKLSTYTYTAIISLFLVCIPICVLWCFMDKLLILTGQDHSISVEARKYSLWVIPAIFGGAISKPLSRYSQAQSLILPMLLSSFAVLCFHLPISWALIFKLELGNIGAAIAFSISSWLYVLFLASYVKLSSSCEKTRAPFSMEAFLCIRQFFRLAVP |
| NtMATE1  [*Nicotiana tabacum*] | MGKSMKSEVEQPLLIAAHGGSSELEEVLSDTQLPYFRRLRYASWIEFQLLYRLAAPSVAVYMINNAMSMSTRIFSGQLGNLQLAAASLGNQGIQLFAYGLMLGMGSAVETLCGQAYGAHRYEMLGVYLQRATVVLSVTGIPLTVVYLFSKNILLALGESKLVASAAAVFVYGLIPQIFAYAVNFPIQKFLQAQSIVAPSAFISLGTLFVHILLSWVVVYKIGLGLLGASLVLSFSWWIIVVAQFIYIIKSERCKA |
| AtEDS5  [*Arabidopsis thaliana*] | MLIKSQRLTLFSPLLSKTRRIPVNSHQTLVAESVITRRTLGAITATPSFHKNPVVIRRRIKLERVTRNCVRIDREIDEEEEEEEKERGDLVKQSIWEQMKEIVKFTGPAMGMWICGPLMSLIDTVVIGQGSSIELAALGPGTVLCDHMSYVFMFLSVATSNMVATSLAKQDKKEAQHQISVLLFIGLVCGLMMLLLTRLFGPWAVTAFTRGKNIEIVPAANKYIQIRGLAWPFILVGLVAQSASLGMKNSWGPLK |
| VcMATE3  [*Vaccinium corymbosum*] | MGTADTTPLLLNNNNLQEGGGGDLQGKAVAVARAFGTESKRLWKIAGPAILTAICQYSLGALTQTFAGFVGDLELAAVSVENSVVAGLAFGVMLGMGSALETLCGQAYGAGQVRMLGVYMQRSWVILLVTACLLVPVYVFSPPILELAGETTEISEAAGKFAIKMLPQLFAYALNFPIQKFLQAQRKVLVMTWVSAIVLVIHVVFSWLLMLKLGWGLTGAAIVLNTSWWLIVIGQLLYIFVTTSDGAWSGFSWLA |
| VcMATE8  [*Vaccinium corymbosum*] | MDNNIEHRLLGSEEEDPTDLKLRVWVESKKIWRVAAPGILARVSQFGLMVVTQSFIGHISEFDLAAYALVQTLTVRFANGILLGMSSATETLCGQAFGAGQHHMMGIYLQRSWIVDFVTATILLPLFIWTTQIFELLGEDTAISILAEKISIWFIPFLYNFVFTLTIQMYLQAQLKNMIVAWLATVSFIFHLVVSWLFVSELDLGIPGAMGALSISSWLVAVGEFVYIFGGWCPLSWKGFSKAAFSDLWPLIKLS |
| VcMATE9  [*Vaccinium corymbosum*] | MVTNIETQPDDELHQPILHSTPLPPPPPPPPPPQPPSTATYQPSTELEGVLSDTQLPLFNRLRLASWIELRLLFRLAGPAVMMYLINNAMSVSTRIYCGHLGNLELAAASLGNQGIQLFVYGLMLGMGSAVETLCGQAYGAHKYEMLGVYLQRSTVVLTLTGIPLTVIYILSKKILLLLGESSAVSSEAAVFVYGLIPQIFAYAINFPIQKFLQSQSIVAPSAYISASTLVLHLVLSWVVVYKLGLGLIGASLVL |
| RsMATE2  [*Raphanus sativus*] | MDSSQNDGAYQPLLQPQLSQATEWNNGELERVLSDVETPVFARLRKATMIESKLLFKLAAPAVIVYMINYLMSMSTQIFSGHLGNLELAAASLGNTGIQVFAYGLMLGMGSAVETLCGQAFGGKKYDMLGVYLQRSAVLLTLTGVLLTFIYVFSEPILLFLGESPEIASAASLFVYGLIPQIFAYAMNFPIQKFLQAQSIVAPSAYIATATLFVHLLLSWLAVYKLGMGLLGASLVLSLSWWIIVAAQFVYIVTS |
| RsMATE3  [*Raphanus sativus*] | MEKDNSFMDPFLSSTEDLDPTTQKALMDYLGVGSPASSLVSFCSTAVDIPPISNVGDFVREFRIESKKLWKLAGPAIFTSMAQFSLGAITQVFAGHISTIALAAVSIENSVIAGFSFGIMLGMGSALETLCGQAFGAGQASLLGVYLQRSWVILSATALMLSLLYIFAAPILTFIGQTATISAMAGLFSIFMIPQIFAYAINYPTAKFLQSQSKIMVMAGISGVALVIHTLLTWLVMSKFHWGLPGLAFVLNTSW |
| RsMATE5  [*Raphanus sativus*] | MDHTSPLLPHGGEVEEDYAPARTWSDVRRVLCTESAKMWLIAAPVGFNVICQYGVSSVTNIFVGHIGEIELSAVSISLSVIGTFSFGFLLGMGSALETLCGQAFGAGQFHMLGVYMQRSWIILFVSCLFLLPIYIFATPVLRLLGQAEEIAVPAGQFTLLTIPQLFSMAFNFPTSKFLQAQSKVTVIASIGFIALLLHVGMLWLFIIVFGLGTNGAALAFNITNWGIAISQIVYVIGWCNDGWTGLSWLAFKEIW |
| RsMATE7  [*Raphanus sativus*] | MSEDIGYNKETACDFPRNPLCIFLSDFKSVLIFDELGLEIARIALPAALALTADPIASLVDTAFIGQIGPVELAAVGVSIALFNQVSRIAIFPLVSITTSFVAEEDACSSQETTVQDHKECIETGINNAKEETQELIPDNNTDSISNESKISSSIFSVSESPVKKRNIPSVSSALIIGAILGLLQAAFLISTARPLLSFMGIKHDSPMLGPAQRYLSLRSLGAPAVLLSLATQGVFRGFKDTTTPLYATVVGDAT |
| RsMATE8  [*Raphanus sativus*] | MEPTTPLLDHGGGDEVKEDYSPARTLSDVKRVLSMESAKLWKIAAPIGFNIICQYGVTSFTNIFVGHIGEIGLSAVSISLSVIGTFSFGFLLGMGSALETLCGQAFGAGQVHMLGVYMQRSWIILFVSCIFLLPIYIFATPVLRFLGQAEEIAVAAGEFTLLTIPQLFSMAFTFPTSKFLQAQSKVIAIAWIGFVALIMHVAMLWLFIVVFGWGTNGAALAFSITNWGTAISQIVYVIGWCNEGWTGLTWLAFKE |
| MdMATE4 | MEKPLLHTAGDGDVELSYKPLLYEGTNEDYAPVRSFDALRRMFWIETVKLWQIAGSTVITMVCMYGNTAVVVLFAGHLGTIELSAISISLTVISIFSFGVMLGMGSALETLCGQAFGAGQVHMLGIYMQRSCIILFVTNIFLLPTYIFATPILKWLGQEDDIANLAGKFSLQIIPHLFSLAVIFPTQKFLQSQRKVKVLAWIAVLALIVEIGMLGLFIYVFDWGTTGAAVAFNVTRWGMAITQVVYIMGWCREGW |
| MdMATE8 | MEEPLLDTAAGGTTEYSNKQLMYEGESEDYAPVRSFGELRQMFWIETVKLWQIGGSAVITIMCMYGTNSVIVLFAGHLGTIELSAISISLSVISTFTYGFMLGMGSALETLCGQAFGAGQVHMLGIYMQRSCIILFVTSFLLLPIYIFATPVLKWLGQEDDIANEAGKFTLQIIPQLFSLAINFPAQKFLKAQRKVKVLAWIAVLALLIHIGMLALFIYVFDWGTSGAAVSFNITRWGISIAQVVYIMGWCYEGW |
| MdMATE7 | MEEPLLHTAAAGADELSNRPPLYEGGNEDYAPVRSFDAFQRMFWIETVKLWQIGVSSVITIICMYGTNAVILLFAGHLGTIQLSAISISLAVISTFTDGLMLGMGSALETLCGQAFGAGQVHMLGIYLQRSWIILFVTTLFLLPVYIFAVPILKWLGQEDDIANLAGKFTLQIIPQIFSLAIYFPAQKFLQAQRKVKVLAWIAFLGLVIHIGMLGLFMYEFDWGTLGAAVSFNITRWGMAIAQVVYIMGWCKEGW |
| MdMATE23 | MEEQEPPLHTAAAGATELSNKPPLYVGGNEDYAQVKSFDALRSMFWIETVKLWQMAGSAVITIICMYGTNAVILLFAGHLGTIQLSAISISLAVISTFTDGLMLGMASALETLCGQAFGAGQVHMLGIYLQRSWIILFVTTLFILPIYIFAAPILKLLGQEDDIANLAGKFTLQIIPQLFSLAINFPAQKFLQAQRKVKMLAWIAMLALVIHIGMIALFMYVFNWGTLGAAVSFNITRWAIAIAQVVYIMGWCKE |
| MdMATE6 | METPLLHGVSDGEFHDYEPVRSFKDARSVAWTETKKLWKIAGPIAFTIICNYGTNTASTMFVGHLGNLELSAVSISLSVISTFSFGFMLGMGSALETLCGQAFGAGRVHMLGVYMQRSWIILFTSCVILTPLYVFSGPILKLLGQEDDVANLAGSFTRYCIPQLFSLAFNFPAQKFLQSQSKVLVLMWIGFIALVVHIGWLFLFVYVFDWGIYGIGIAFSLTGWETVIAQNIYIMNWCKEGWTGFSWLAFKDIWA |
| MdMATE21 | MVTPVPLLHAVNGGEFHDYEPVRNFRDARSVAWNETKKLWKIAGPIAFTIICNFGNNTATTMFVGHLGNLQLSAVSISLSVICTFSFGFMLGMGSALETLCGQAFGAGHVNMLGVYMQRSWIILFTSCVILTPIYIFSAPILKLLGQDDDVANLAGTFTIYCIPQLFSLAFNFPAQKFLQAQSKVLVLAWIGFISLLVQIGMLSLFIYVFDWGIYGAGIAFGITGWVIVIAQNIYIMKWCKEGWTGFSWLAFKDI |
| MdMATE22 | METPLLTIKAEAAAAADYAPVRSFGEAMKVSWKEAVKLWRVATLVACTSLFQYLVQSITTVFVGHLGDVELSAVSLSLGVICNIPFGFLLGMATALGTLCGQAYGAGQVGQLGIYMQRSWIVLFIACIILSPVFIFAAPILKFLGQEHGIADPAGVYSLKIIPQMFSYAINLPTQRFLQAQSKVLVITLIAFAALIIQTGLLHLFINVFGLGTTGAAVAYDITNWGVAIGQVGYVMVCCKEEWTGFSWLAFREIW |
| MdMATE20 | MVQSPTLMIDGEDDAVSTFEPADLHHAPPAFIGSSDGDYPVIRSFQDAKSICFVESTKLWSIAGPIAFNILCNYGVNSFTNIFVGHIGNVELSAVAISLSVISNFSFGFLLGMASALETLCGQAFGAGQVDMLGVYMQRSWIMLFAACIAILPLYIYSTPVLKLLGQEDDIADLAGKFSIQTIPQMVSLAINFPTQKFLQAQSKVGVLAWIGFITLIAHVGILFLFIKVFGWGTSGAAAAYNISAWGMALAQVVY |
| MdMATE24 | MEIGNDQNTPLLVSNHSGHEDGGEEKVGFVKQYGIESKKLWKIAGPAIFTSLCQYSLGALTQTFAGFVGDLELAAVSIENSVIAGLAFGVMLGMGSALETLCGQAYGAGQIRMLGVYMQRSWIILLVTACAMVPIYVWSPPILKLFGETTEISEAAGRFALLMLPQLFAYALNFPIQKFLQAQRKVSVIAWISGAVLVEHAVLSWLLMIKLGWGLTGAAITLNASWWLIIIGQLLYIFITKSDGAWSGFSWLAFS |
| MdMATE49 | MPTTSRMAIVQEDDPVVVHAGDIPPIVSFRGFSRQFYKETKKLWYLAGPAIFTSLSQYSIGAITQIFAGHVGTLELATVSVENSVIAGFSFGFMYGMGSALETLCGQAFGAGQLDMLGIYMQRSWVILNATAVLLSFLYIFAEQLLKMIGQPDDISKAAGQFAIWMIPQLFAYAMNFPLAKFLQSQSKIMVMAAISAVALVLHTLFSWLLTLKLGWGLVGAAVVLNTSWWFIVLAQLVYIFSGTCGRAWAGFSWK |
| MdMATE3 | MASNSALVEAEVPLLDDLASTVRLKREHNDDENQATTLVRRSWIESKKLWHIVGPAIFSRVASYSMLVITQAFAGHLGDLELAAISIANNVIVGFDFGLLLGMASALETLCGQAYGAKKYYMLGVYMQRSWIVLFLCCILLLPIYLFASPFLKLLGQPAEVAELSGVVSMVMIPLHFSFAFQFPLQRFLQSQLKTAVIAWVSLVSLAVHVFVSWLFVYRLQFGVIGTAITINFSWWVLVFGLLGYTLFGGCPLTW |
| MdMATE25 | MKGSVEEKVMDRQKYSLLAIESSSTSEMKKIATDFLSRVWIESKKLWHIVGPDIINRLAGYSMTVITQAFAGHLGDVELASISMANNIIVGFGYGLLLGMASALATLCGQTFGAKRYHMLGIYMQRSWIVLFSCCILLLPVYLYASPILKLIGQSDEVAEQSGALALWLIPLHFSYAFQFPLQRFLQCQLKNFVTLWVSLAVLVLHSVTTWILVSVLDFGVVGAAIALDISCWACGLGLFWYVVSGGCPLSWVGF |
| MdMATE43 | MLFTDSFNNSYFTKIPNCYVTPRANSLSLSLSLSTNIGSQQTRVQFLISLMGNRNSDQDGDLTQSLLPEALPQNSAANDRDQKTDLSLRVWLETKKLWKIVGPAIFSRVATFSMNVITQAFAGHLGEVELASVSIGITLVIGFNFGLLLGMASALETLCGQAFGAKRYHMLGIYLQRSWTVLFLCCIALLPVYIFVTPILKLLGQSDDVAALSGAVALWLIPLHFSFAFYFPLTRFLQSQLQNIVIAWVSLVALL |
| MdMATE32 | MGEEIKGSLLVKETGVGEEEARIIDGDEELSLKRRVWIETKKMWVVAGPAIFTRVASFGTNVISQAFIGHIGSAQLAAFSLVFTVLVRFANGILLGMASALETLCGQSYGAKQYNMLGIHLQRSWIVLFVSTCLLIPLFVFTTPIFEALGQAENISEIAGHISLWVIPVLFAFVVSFTCQMYLQAQSKNMIIAYVSAISIGIHIFLCWLLSVKLKFGVPGVMVSTLIAYWLPNLGQLLFVLCGGCPETWTGFSML |
| MdMATE40 | MGEDIKRSLLVKETSAGEEETRVIDGEEELSLKRRVWIEIKKMWLVAGPAIFTRVASFGTNVVSQAFIGHIGSLELAAFSLVFTVLVRFGNGILLGMASALETLCGQSYGAKQYNMLGIHLQRSWIVLCVGTLFLIPLFVFTTPIFEALGQADNISEVAGYISLWVIPVLFAFVVSFTCQMYLQAQSKNMIVAYVSAVSIGIHIFLCWLLSVKFKFGVPGVLVSTIISYWLPNVGQLLFILCGGCPETWTGFSML |
| MdMATE1 | MGSQEEYQPLLIRLDSYSQIPNLSSSAIEEFLEHKPVAVRWWPKLVAWESRLLWILSGSSIAVSIFNYMLSFVTLMFCGHLGALELAGASIASVGIQGLAYGIMLGMASAVQTVCGQAYGAKQLPAMGIICQRAIILHLGAAVLLTFVYWWSGPILIAIGQTEDIAEQGQVFARGIIPQLYAFAINCPQQRFLQAQNIVNPLAYMSFGVFLVHILLTWVVVYVVDYGLMGAALTLSLSWWLLVITYGIYILVSPM |
| MdMATE52 | MGSVEEIQQPLLLGFDTHSRLADLSSPAVEEFLGHRPVALWWWPRLVVWESRLLWILSGSSIIVSVFNYMLSFVTLMFCGHLSALELAGASIASVGIQGLAYGIMLGMASAVQTVCGQAYGAKQLPAMGIICQRAIILHLGAAILLSFVYWWSGPILVAIGQSEEIAEQGQIFARGIIPQLFAFAINCPQQRFLQAQNIVNPLAYMSVGVFILHILLNWVVVFVVDYGLIGAALTLSFSWWLLVIIYGLYILLSP |
| MdMATE2 | MGTAEEYQPLLNGLDSHSRIPDLSSTAVEEFLEHKPVAVRWWLRLVAWESRLLWTLSGSSIIVSIFNYMLSFTTLMFCGHLSALELAGASIASVGIQGLAYGIMLGMASAVQTVCGQAYGARQYPAMGIICQRAIVLHLGAAVLLTFLYWWSGPILIAIGQTEEIAEQGQVFARGIVPQLYAFAINCPQQRFLQAQNIVNPLAFMSFGVFLVHILLSWVVVYVADYGLTGAALTLSFSWWLLVIVYGIYIVVSPK |
| MdMATE17 | MEYSSDSDLSEPMLVPKTSSLQQIVSSELEDTLNNTDLSNFQRLRTATWLETKTLYRLAAPAVVVYLLNNVISMSTQILCGHLGNLELAASSLGNTGIQVFAYGLMLGMGSAVETLCGQAYGAHKYEMLGIYMQRSTILLVATGIPLTIVYIFSKPLLLALGESASISAAAAVFVYGLIPQIFAYACNFPIQKFLQAQSIVFPSAYISAGALVVHIVLSWVVVYKLDWGLLGAALMLSFSWWIIVVAQFVYIVWT |
| MdMATE51 | MANNELQHPLLESYHPLPPTSTQSSKHSHDEDASRELEQVLSDTDKPFSQRLKPALWIESKLLVILAAPAIIVYVINYVMSMSTQIFSGHLGNLELAASSLGNNGIQMFAYGLLLGMGSAVETLCGQAYGAQKYEMLGIYLQRSTVLLFFPGVLLTIIYIFSEPILLLLGESPSIASSAAVYVYGLIPQIFAYVVNFPIQKFMQAQSIVAPSAYISTGTLVIHIVLSWVAVYTLGLGLLGASLVLSLSWWITVVA |
| MdMATE16 | MPKSTYVHGQRVKYIRSLTWTSYIINAASYFQNSKHQFLSMHRTVAHFCHTPHHLFGPKMGSQHPLHQPILHSEPEPVAQASSDGDGVDFLLEKVLSDTQLPSFKRFRSATWIELKLLFRLAAPAVLVYVINNSMSLSARVFAGHLGNLELAAASLGNNGIQLLAYGLMLGMGSAVETLCGQAYGAQKYDMLSIYLQRATIVLSLTGLPLLAIFLLTKPMLILLGEPPAVASAAAVFVYGLIPQIFAYAVNFPIQ |

**Supplementary Table S3 Primer sequences used in the study**

|  | Primer name | Genebank | Froward primer | Reverse primer |
| --- | --- | --- | --- | --- |
| CDS amplification | MdMYB10(OE) | MDP0000259614 | CGGGATCCATGGAGGGATATAACGAAAACCTGAG | CGGGATCCATTCTTCTTTTGAATGATTCCAAAGGTCCG |
|  | MdMYB10(i) | MDP0000259614 | GGGGACAAGTTTGTACAAAAAAGCAGGCAGGTGGTCATTGATTGCT | GGGGACCACTTTGTACAAGAAAGCTGGGTATTCCAAAGGTCCGTGCTA |
|  | MdMYB9(OE) | MDP0000210851 | GCTCTAGAATGGGAAGGAGTCCGTGTTG | CGAGCTCTTAGACTACAACATTTTCTTGGGAAGGC |
|  | MdMYB9(i) | MDP0000210851 | GGGGACAAGTTTGTACAAAAAAGCAGGCTGGGAAGGAGTCCGTGTTG | GGGGACCACTTTGTACAAGAAAGCTGGGTCAGCGGCAGTTGATGTTT |
|  | MdMATE8(i) | MDP0000210851 | GGGGACAAGTTTGTACAAAAAAGCAGGACACTCCAAATCATCCCTCAA | GGGGACCACTTTGTACAAGAAAGCTGGGTTCCAACCCTCATAACACCAAC |
| qRT-PCR | *MdUBQ* | DQ438989 | CTCCGTGGTGGTTTTTAAGT | GGAGGCAGAAACAGTACCAT |
|  | *MdMYB10* | MDP0000259614 | TGCCTGGACTCGAGAGGAAGACA | CCTGTTTCCCAAAAGCCTGTGAA |
|  | *MdMYB9* | MDP0000210851 | CCGCAAACCCATTAGAGC | GTCCAGCAGCCGTCATTT |
|  | *MdbHLH* | MDP0000225680 | CCTACAAGCCACGATTACTACAA | TGAACGCAATGAGGAATACAA |
|  | *MdbZIP* | MDP0000265875 | TCCAATAGGGAATCGGCACG | AATCCTTCACCTCCACCAA |
|  | *MdCHS* | MDP0000686666 | GGAGACAACTGGAGAAGGACTGGAA | CGACATTGATACTGGTGTCTTC |
|  | *MdCHS* | MDP0000575740 | CGTTCCTGGGCTTATTTCC | CAGCAGACTTCCTCCTCACTT |
|  | *MdF3'H* | MDP0000286933 | TTCAACATCGGCGACTTCA | TTCAACAATGGCGGTCAAG |
|  | *MdDFR* | MDP0000494976 | ACATTATGGCATCATCAAGCA | TCAAACCCTATCTCCCTCAACT |
|  | *MdLDOX* | MDP0000360447 | AAGCGTGACTTGTCTATTTG | ACTTCTTTCTCCAGCCTCC |
|  | *MdLODX* | MDP0000788934 | CCAAGTGAAGCGGGTTGTGCT | CAAAGCAGGCGGACAGGAGTAGC |
|  | *MdUFGT* | MDP0000543445 | CCACCGCCCTTCCAAACACTCT | CACCCTTATGTTACGCGGCATGT |
|  | *MdGST* | MDP0000252292 | AGTTGTAGAAGATGGTGACT | CAGGTCGTTGAAGTTGTG |
|  | *MdMATE* | MDP0000175055 | CTTCTTACTGTTGCCGATT | TCTGAGCCTGGAGGAACTTTT |
| Y1H | MdMYB10-pGADT7 | MDP0000259614 | CGGGATCCATGGAGGGATATAACGAAAACCTGAG | CCGCTCGAGATGATTCCAAAGGTCCGTGCT |
|  | MdMYB9-pGADT7 | MDP0000210851 | CGAGCTCGCTCTAGAATGGGAAGGAGTCCGTG | CCGCTCGAGGCTCTAGAGACTACAACATTTTCTTGGGAAG |
|  | MdMATE8-pHIS2 | MDP0000175055 | GAGCTCATTAGCCGGTTGACTTTCAAAA | GAATTCTTAGTTAGGCGTAAGATTTAAGTTTTG |

**Supplementary Table S4.** **Summary of the sequencing results and correlation coefficient values.**

| **TABLE4 \| Summary of the sequencing results.** | | | | | | | | | | | |
| --- | --- | --- | --- | --- | --- | --- | --- | --- | --- | --- | --- |
| **Sample name** | | **Raw reads** | **Clean reads** | **Clean reads rate (%)** | **Mapping to genome** | | **Mapped position (%)** | | | **Q30 (%)** |  |
|  |  |  |  |  |  | **Rate (%)** | **Exon** | **Intron** | **Intergenic** |  |  |
| Control-24 | C1 | 33763726 | 32022902 | 94.84 | 29041737 | 90.69 | 83.8 | 1.8 | 14.3 | 90.82 |  |
|  | C2 | 27497162 | 26395420 | 95.99 | 24058541 | 91.15 | 84.2 | 2 | 13.8 | 91.65 |  |
|  | C3 | 27168760 | 26082490 | 96 | 23644410 | 90.65 | 84.4 | 1.8 | 13.8 | 91.17 |  |
| ALA-24 | T4 | 27298414 | 26275352 | 96.25 | 23692171 | 90.17 | 83.6 | 1.9 | 14.5 | 91.22 |  |
|  | T5 | 26376820 | 25078268 | 95.08 | 22636521 | 90.26 | 83 | 2.2 | 14.8 | 91.5 |  |
|  | T6 | 29591274 | 28299616 | 95.64 | 25447680 | 89.92 | 83.5 | 2.1 | 14.4 | 91.06 |  |
| Control-48 | C7 | 28636090 | 27085828 | 94.59 | 24533552 | 90.58 | 84.1 | 2 | 13.9 | 91.52 |  |
|  | C8 | 30744152 | 29388298 | 95.59 | 26789159 | 91.16 | 84.8 | 1.9 | 13.3 | 91.6 |  |
|  | C9 | 28614420 | 27468658 | 96 | 24894274 | 90.63 | 84.9 | 1.8 | 13.3 | 91.52 |  |
| ALA-48 | T10 | 31803012 | 30571964 | 96.13 | 27715364 | 90.66 | 83.7 | 2 | 14.3 | 91.41 |  |
|  | T11 | 27520458 | 26492144 | 96.26 | 23978432 | 90.51 | 83.7 | 2 | 14.3 | 91.69 |  |
|  | T12 | 25503524 | 24457092 | 95.9 | 22301014 | 91.18 | 83.9 | 2.1 | 14 | 91.16 |  |
| Control-72 | C13 | 29788534 | 28481664 | 95.61 | 25810073 | 90.62 | 84.8 | 1.9 | 13.3 | 91.17 |  |
|  | C14 | 31353898 | 30074766 | 95.92 | 27346568 | 90.93 | 84.9 | 1.9 | 13.2 | 91.53 |  |
|  | C15 | 28556660 | 26791794 | 93.82 | 24254091 | 90.53 | 84.1 | 2.1 | 13.8 | 89.2 |  |
| ALA-72 | T16 | 27268836 | 26004326 | 95.36 | 23611323 | 90.8 | 84 | 1.9 | 14.1 | 90.94 |  |
|  | T17 | 24099168 | 22679590 | 94.11 | 20466525 | 90.24 | 84.2 | 1.9 | 13.9 | 90.5 |  |
|  | T18 | 28898534 | 27497178 | 95.15 | 24866350 | 90.43 | 84 | 2 | 14.1 | 90.7 |  |
| Total | -- | 514483442 | 491147350 | -- | 445087785 | -- | -- | -- | -- | -- |  |
| Mean | -- | 28582413 | 27285964 | 95.46 | 24727099 | 90.62 | -- | -- | -- | -- |  |

**Supplementary Table S4. Summary of the sequencing results and correlation coefficient values.**

| **TABLE 4 \| Correlation coefficient vlaues** | | | | | | | | | | | | | | | | | | |
| --- | --- | --- | --- | --- | --- | --- | --- | --- | --- | --- | --- | --- | --- | --- | --- | --- | --- | --- |
| **Sample** | **C1** | **C2** | **C3** | **T4** | **T5** | **T6** | **C7** | **C8** | **C9** | **T10** | **T11** | **T12** | **C13** | **C14** | **C15** | **T16** | **T17** | **T18** |
| **C1** | 1 | 0.968 | 0.966 | 0.947 | 0.946 | 0.942 | 0.96 | 0.954 | 0.96 | 0.951 | 0.951 | 0.942 | 0.951 | 0.938 | 0.946 | 0.957 | 0.953 | 0.947 |
| **C2** | 0.968 | 1 | 0.97 | 0.943 | 0.943 | 0.94 | 0.961 | 0.954 | 0.959 | 0.947 | 0.944 | 0.94 | 0.951 | 0.94 | 0.948 | 0.951 | 0.948 | 0.942 |
| **C3** | 0.966 | 0.97 | 1 | 0.944 | 0.944 | 0.941 | 0.957 | 0.95 | 0.954 | 0.947 | 0.943 | 0.94 | 0.946 | 0.936 | 0.945 | 0.949 | 0.945 | 0.942 |
| **T4** | 0.947 | 0.943 | 0.944 | 1 | 0.968 | 0.967 | 0.941 | 0.932 | 0.942 | 0.968 | 0.964 | 0.966 | 0.942 | 0.945 | 0.948 | 0.955 | 0.951 | 0.965 |
| **T5** | 0.946 | 0.943 | 0.944 | 0.968 | 1 | 0.967 | 0.937 | 0.928 | 0.939 | 0.966 | 0.962 | 0.964 | 0.935 | 0.938 | 0.942 | 0.953 | 0.951 | 0.963 |
| **T6** | 0.942 | 0.94 | 0.941 | 0.967 | 0.967 | 1 | 0.935 | 0.928 | 0.936 | 0.963 | 0.961 | 0.962 | 0.932 | 0.936 | 0.939 | 0.953 | 0.95 | 0.963 |
| **C7** | 0.96 | 0.961 | 0.957 | 0.941 | 0.937 | 0.935 | 1 | 0.968 | 0.969 | 0.949 | 0.947 | 0.942 | 0.964 | 0.948 | 0.958 | 0.958 | 0.956 | 0.944 |
| **C8** | 0.954 | 0.954 | 0.95 | 0.932 | 0.928 | 0.928 | 0.968 | 1 | 0.968 | 0.94 | 0.942 | 0.934 | 0.96 | 0.938 | 0.95 | 0.958 | 0.957 | 0.939 |
| **C9** | 0.96 | 0.959 | 0.954 | 0.942 | 0.939 | 0.936 | 0.969 | 0.968 | 1 | 0.949 | 0.949 | 0.944 | 0.963 | 0.949 | 0.957 | 0.958 | 0.956 | 0.946 |
| **T10** | 0.951 | 0.947 | 0.947 | 0.968 | 0.966 | 0.963 | 0.949 | 0.94 | 0.949 | 1 | 0.968 | 0.968 | 0.95 | 0.951 | 0.955 | 0.96 | 0.956 | 0.967 |
| **T11** | 0.951 | 0.944 | 0.943 | 0.964 | 0.962 | 0.961 | 0.947 | 0.942 | 0.949 | 0.968 | 1 | 0.964 | 0.949 | 0.942 | 0.948 | 0.964 | 0.961 | 0.968 |
| **T12** | 0.942 | 0.94 | 0.94 | 0.966 | 0.964 | 0.962 | 0.942 | 0.934 | 0.944 | 0.968 | 0.964 | 1 | 0.946 | 0.948 | 0.952 | 0.956 | 0.953 | 0.966 |
| **C13** | 0.951 | 0.951 | 0.946 | 0.942 | 0.935 | 0.932 | 0.964 | 0.96 | 0.963 | 0.95 | 0.949 | 0.946 | 1 | 0.963 | 0.968 | 0.956 | 0.953 | 0.948 |
| **C14** | 0.938 | 0.94 | 0.936 | 0.945 | 0.938 | 0.936 | 0.948 | 0.938 | 0.949 | 0.951 | 0.942 | 0.948 | 0.963 | 1 | 0.969 | 0.945 | 0.941 | 0.949 |
| **C15** | 0.946 | 0.948 | 0.945 | 0.948 | 0.942 | 0.939 | 0.958 | 0.95 | 0.957 | 0.955 | 0.948 | 0.952 | 0.968 | 0.969 | 1 | 0.953 | 0.95 | 0.952 |
| **T16** | 0.957 | 0.951 | 0.949 | 0.955 | 0.953 | 0.953 | 0.958 | 0.958 | 0.958 | 0.96 | 0.964 | 0.956 | 0.956 | 0.945 | 0.953 | 1 | 0.967 | 0.962 |
| **T17** | 0.953 | 0.948 | 0.945 | 0.951 | 0.951 | 0.95 | 0.956 | 0.957 | 0.956 | 0.956 | 0.961 | 0.953 | 0.953 | 0.941 | 0.95 | 0.967 | 1 | 0.961 |
| **T18** | 0.947 | 0.942 | 0.942 | 0.965 | 0.963 | 0.963 | 0.944 | 0.939 | 0.946 | 0.967 | 0.968 | 0.966 | 0.948 | 0.949 | 0.952 | 0.962 | 0.961 | 1 |

**Supplementary Table S5.** **Gene expression quantification**

| FPKM Interval | Control-24_1 | Control-24_2 | Control-24_3 | ALA-24_1 | ALA-24_2 | ALA-24_3 | Control-48_1 | Control-48_2 | Control-48_3 | ALA-48_1 | ALA-48_2 | ALA-48_3 | Control-72_1 | Control-72_2 | Control-72_3 | ALA-72_1 | ALA-72_2 | ALA-72_3 |
| --- | --- | --- | --- | --- | --- | --- | --- | --- | --- | --- | --- | --- | --- | --- | --- | --- | --- | --- |
| 0~1 | 34805  (54.80%) | 34490  (54.30%) | 34720  (54.66%) | 34078  (53.65%) | 34097  (53.68%) | 34159  (53.78%) | 34515  (54.34%) | 34947  (55.02%) | 34377  (54.12%) | 34060  (53.63%) | 34169  (53.80%) | 33915  (53.40%) | 34107  (53.70%) | 33870  (53.33%) | 33694  (53.05%) | 34442  (54.23%) | 34548  (54.39%) | 33947  (53.45%) |
| 1~3 | 6407  (10.09%) | 6374  (10.04%) | 6426  (10.12%) | 6262  (9.86%) | 6319  (9.95%) | 6314  (9.94%) | 6464  (10.18%) | 6446  (10.15%) | 6375  (10.04%) | 6230  (9.81%) | 6349  (10.00%) | 6197  (9.76%) | 6304  (9.93%) | 6189  (9.74%) | 6344  (9.99%) | 6397  (10.07%) | 6355  (10.01%) | 6349  (10.00%) |
| 3~15 | 11609  (18.28%) | 11852  (18.66%) | 11733  (18.47%) | 11782  (18.55%) | 11858  (18.67%) | 11796  (18.57%) | 11797  (18.57%) | 11716  (18.45%) | 11880  (18.70%) | 11865  (18.68%) | 11841  (18.64%) | 11946  (18.81%) | 11982  (18.86%) | 11863  (18.68%) | 12030  (18.94%) | 11863  (18.68%) | 11825  (18.62%) | 11811  (18.60%) |
| 15~60 | 7570  (11.92%) | 7532  (11.86%) | 7364  (11.59%) | 8312  (13.09%) | 8150  (12.83%) | 8127  (12.80%) | 7593  (11.95%) | 7295  (11.49%) | 7682  (12.09%) | 8250  (12.99%) | 8122  (12.79%) | 8367  (13.17%) | 8017  (12.62%) | 8390  (13.21%) | 8258  (13.00%) | 7760  (12.22%) | 7758  (12.21%) | 8343  (13.14%) |
| >60 | 3124  (4.92%) | 3267  (5.14%) | 3272  (5.15%) | 3081  (4.85%) | 3091  (4.87%) | 3119  (4.91%) | 3146  (4.95%) | 3111  (4.90%) | 3201  (5.04%) | 3110  (4.90%) | 3034  (4.78%) | 3090  (4.86%) | 3105  (4.89%) | 3203  (5.04%) | 3189  (5.02%) | 3053  (4.81%) | 3029  (4.77%) | 3065  (4.83%) |

**Supplementary Table S6.** **DEGs between ALA-treated and control calli.**

| Gene_id | 24h | 48h | 72h | Gene_id | 24h | 48h | 72h | Gene_id | 24h | 48h | 72h | Gene_id | 24h | 48h | 72h |
| --- | --- | --- | --- | --- | --- | --- | --- | --- | --- | --- | --- | --- | --- | --- | --- |
| MDP0000014856 | 1.0332 |  |  | MDP0000196672 | -2.1984 |  |  | MDP0000273384 | 1.0429 |  |  | MDP0000535947 |  | 1.7024 |  |
| MDP0000022760 | -1.6192 |  |  | MDP0000196740 | 1.1987 |  |  | MDP0000273828 | 1.2145 |  |  | MDP0000538331 | -2.0873 |  |  |
| MDP0000031172 | 1.9155 |  |  | MDP0000196862 |  | 1.0859 |  | MDP0000274409 |  |  | 1.089 | MDP0000541805 |  |  | -1.0151 |
| MDP0000032171 |  | -1.5745 |  | MDP0000196876 |  | 1.4136 |  | MDP0000274423 |  |  | 1.0277 | MDP0000542815 |  | -2.3481 |  |
| MDP0000044356 |  | 1.5905 |  | MDP0000196922 | -1.4873 |  |  | MDP0000274441 | 1.9352 |  |  | MDP0000542944 | 3.2861 | 1.8143 |  |
| MDP0000059789 | 1.0107 |  |  | MDP0000197025 |  |  | -1.0249 | MDP0000274766 | -1.8905 |  |  | MDP0000543445 | 2.8258 | 1.9941 | 1.4211 |
| MDP0000064909 |  | 1.2131 |  | MDP0000197224 |  | -1.2237 |  | MDP0000274908 |  | -2.4928 |  | MDP0000543718 |  | -1.4561 |  |
| MDP0000065186 |  | -1.1354 |  | MDP0000197283 | 2.0613 | 1.34 |  | MDP0000275026 | -1.4827 |  |  | MDP0000544274 | -1.502 |  |  |
| MDP0000073873 | -1.0923 |  |  | MDP0000197297 | -1.2886 |  |  | MDP0000275151 | 1.1569 |  |  | MDP0000544533 |  |  | 1.3327 |
| MDP0000091523 |  | 2.0758 |  | MDP0000197360 | 1.2838 |  |  | MDP0000275985 |  | -1.5429 |  | MDP0000545463 | -2.3867 |  |  |
| MDP0000094767 |  | 1.1035 |  | MDP0000197977 |  | 1.4563 |  | MDP0000276215 |  | -2.0055 |  | MDP0000545558 |  | -1.4983 |  |
| MDP0000096349 |  | -1.2632 |  | MDP0000198054 |  | -5.5322 |  | MDP0000276431 | -1.8997 |  |  | MDP0000546099 |  |  | -2.1407 |
| MDP0000103621 | -1.2585 |  |  | MDP0000198217 | -1.5064 |  |  | MDP0000276889 | 1.6929 |  |  | MDP0000547254 |  |  | -1.4215 |
| MDP0000119262 |  | -1.2071 |  | MDP0000198843 |  |  | 1.4934 | MDP0000277088 |  |  | -1.2258 | MDP0000547283 |  | 1.7022 |  |
| MDP0000119275 |  | 1.0831 |  | MDP0000199052 | 1.0302 |  |  | MDP0000277457 | -1.2242 |  |  | MDP0000547655 |  |  | -2.1372 |
| MDP0000119425 | 1.0309 |  |  | MDP0000199202 | -1.2191 |  |  | MDP0000277477 |  | 2.59 |  | MDP0000551192 | -1.963 |  |  |
| MDP0000119931 | 1.8971 |  |  | MDP0000199698 | 1.1042 |  |  | MDP0000277525 |  | 1.3147 |  | MDP0000552015 |  |  | 1.244 |
| MDP0000119961 | -1.033 |  |  | MDP0000199827 | 1.4709 |  |  | MDP0000277906 | 1.049 |  |  | MDP0000554480 | -4.0854 |  |  |
| MDP0000120120 | 1.1115 |  |  | MDP0000199837 |  |  | -1.314 | MDP0000277990 | -2.7494 |  |  | MDP0000554950 | -1.3377 |  |  |
| MDP0000120125 | -1.3762 |  |  | MDP0000200231 |  | 1.0363 |  | MDP0000278249 | -1.1605 |  |  | MDP0000555486 | 1.6084 |  |  |
| MDP0000120180 |  | -1.729 |  | MDP0000200564 | -2.1941 | -2.061 |  | MDP0000278259 | -1.0135 |  |  | MDP0000556996 | 1.0073 |  |  |
| MDP0000120214 |  |  | 1.1816 | MDP0000200635 |  | -1.5497 |  | MDP0000278762 | -2.2637 |  |  | MDP0000557169 |  | -1.179 |  |
| MDP0000120294 |  | -1.1793 |  | MDP0000200718 | -1.4069 |  |  | MDP0000279515 | 1.0835 |  |  | MDP0000557431 | 1.2442 |  |  |
| MDP0000120347 | 2.9472 | 2.9372 |  | MDP0000200780 | 2.3069 |  |  | MDP0000279576 | -2.0722 |  |  | MDP0000557979 |  | -1.1493 |  |
| MDP0000120398 |  |  | -1.0405 | MDP0000201071 |  |  | 1.2071 | MDP0000279658 |  |  | -1.0104 | MDP0000559039 | -2.2236 |  |  |
| MDP0000120460 |  | 1.7927 |  | MDP0000201072 | 1.2289 |  |  | MDP0000280208 |  | 1.4754 |  | MDP0000560112 | 1.2693 | 1.4319 |  |
| MDP0000120467 | -2.4828 |  |  | MDP0000201389 | 1.74 |  |  | MDP0000280265 |  | -3.1125 |  | MDP0000560645 | -1.6725 |  |  |
| MDP0000120881 |  |  | 1.1691 | MDP0000201559 |  |  | 1.0502 | MDP0000280307 |  | 1.2715 |  | MDP0000561026 |  | 1.4764 |  |
| MDP0000120921 |  | -1.9647 | -1.1974 | MDP0000201574 |  |  | -1.1658 | MDP0000280527 |  |  | 1.3974 | MDP0000566690 | -1.1026 |  |  |
| MDP0000120995 | -1.7861 |  |  | MDP0000201866 |  | 1.7254 |  | MDP0000280609 | -1.4509 |  |  | MDP0000567084 |  | -1.1392 |  |
| MDP0000121048 |  |  | -1.5732 | MDP0000201967 |  |  | 1.2936 | MDP0000280662 |  |  | 1.3565 | MDP0000568045 |  |  | -1.1556 |
| MDP0000121203 | 1.2898 |  |  | MDP0000202131 |  | 1.4586 |  | MDP0000281079 | -1.6477 |  |  | MDP0000568341 | 2.0146 |  |  |
| MDP0000121207 | 1.015 |  |  | MDP0000202144 |  |  | 1.2333 | MDP0000281334 |  | -1.1598 |  | MDP0000569128 | -1.9658 |  |  |
| MDP0000121385 | -1.0776 |  |  | MDP0000202577 |  | 2.2015 |  | MDP0000281449 | -1.9227 |  |  | MDP0000570178 | -2.1205 |  |  |
| MDP0000121657 |  |  | -1.1254 | MDP0000202749 |  | -1.16 |  | MDP0000281883 |  | 1.451 |  | MDP0000570395 |  | -2.2889 |  |
| MDP0000121669 | -2.6724 | -1.4411 |  | MDP0000202898 | -1.1304 |  |  | MDP0000282090 |  | 2.5771 |  | MDP0000571465 | 1.985 | 1.0898 |  |
| MDP0000121897 | -1.2202 |  |  | MDP0000203352 | 1.7302 |  |  | MDP0000282332 | -2.255 |  | -1.3909 | MDP0000573302 | 2.3054 | 2.1803 | 2.1232 |
| MDP0000122199 |  |  | 1.0506 | MDP0000203773 | 1.048 |  |  | MDP0000282535 | 1.7936 |  |  | MDP0000573746 | 2.6764 |  |  |
| MDP0000122458 | 1.0569 |  |  | MDP0000203818 | -1.2137 |  |  | MDP0000282680 |  |  | -1.3115 | MDP0000574555 |  | 3.0772 |  |
| MDP0000122972 |  | 1.1614 |  | MDP0000203919 | 1.1527 |  |  | MDP0000282711 | -1.3009 |  |  | MDP0000574641 | 1.1115 |  |  |
| MDP0000123135 |  | 1.5685 |  | MDP0000204472 |  | -1.7222 |  | MDP0000282836 |  |  | -1.6519 | MDP0000575740 | 3.3995 | 2.2017 | 1.6808 |
| MDP0000123455 |  |  | 1.3185 | MDP0000204616 |  | -1.751 |  | MDP0000283002 |  |  | 1.9632 | MDP0000576682 | -3.5244 |  |  |
| MDP0000123466 |  | -1.4279 |  | MDP0000205024 |  | 1.5172 |  | MDP0000283260 | -1.5968 |  |  | MDP0000578146 |  |  | -1.2058 |
| MDP0000123573 |  |  | 1.1426 | MDP0000205665 | 1.2774 |  | -1.5582 | MDP0000283317 |  | 1.0125 |  | MDP0000578465 | -1.0518 |  |  |
| MDP0000123832 | -1.3141 | -1.0392 |  | MDP0000205890 |  |  | 1.1742 | MDP0000283534 | 1.3148 |  |  | MDP0000579343 | -1.8901 |  |  |
| MDP0000123890 |  | 1.3305 |  | MDP0000205904 |  | 1.2267 |  | MDP0000283589 |  | 2.9841 |  | MDP0000579912 |  | 1.6327 |  |
| MDP0000123998 |  | -1.553 |  | MDP0000206052 | 1.8528 |  |  | MDP0000283879 | 1.2598 |  |  | MDP0000580411 | 1.0184 | 1.0115 |  |
| MDP0000124016 |  | 2.9444 |  | MDP0000206109 | 1.3266 |  |  | MDP0000283949 | 1.7797 |  |  | MDP0000581514 | -1.403 |  |  |
| MDP0000124103 |  | -1.8055 |  | MDP0000206212 | -2.7231 |  |  | MDP0000284608 |  | -1.3223 |  | MDP0000581822 | 1.159 |  |  |
| MDP0000124491 |  | 1.4562 |  | MDP0000206276 | 1.4935 |  |  | MDP0000284920 |  | 1.0055 |  | MDP0000586415 |  | -1.0162 |  |
| MDP0000124555 | 1.6788 | 1.1321 |  | MDP0000207170 | -2.2578 |  |  | MDP0000285032 | -1.8275 | -2.5097 |  | MDP0000589856 |  | -1.1829 |  |
| MDP0000124654 | 2.1483 | 1.2541 |  | MDP0000207225 |  |  | 1.231 | MDP0000285142 | 1.2016 | 1.0021 |  | MDP0000592603 | -1.3874 |  |  |
| MDP0000124672 |  |  | -1.4333 | MDP0000207554 | 1.34 |  |  | MDP0000285414 |  |  | 1.2789 | MDP0000592869 | -1.9016 |  |  |
| MDP0000124838 |  | -3.1279 |  | MDP0000207754 | 1.714 | 1.8955 |  | MDP0000285642 | -1.2682 |  |  | MDP0000594960 | -1.9886 |  |  |
| MDP0000124968 |  |  | -3.4366 | MDP0000207854 |  | 1.0808 |  | MDP0000286425 | -1.0464 |  |  | MDP0000595961 | -2.1163 |  |  |
| MDP0000124995 |  |  | -1.4095 | MDP0000208200 | -1.6284 |  |  | MDP0000286444 |  | -1.3246 |  | MDP0000596615 |  |  | 1.0959 |
| MDP0000125070 | 1.0893 |  | -1.2237 | MDP0000208204 | 1.0487 |  | -1.274 | MDP0000286811 |  | 1.0509 |  | MDP0000597996 |  | -2.3689 |  |
| MDP0000125282 |  | 1.0761 |  | MDP0000208320 | 1.5116 |  |  | MDP0000286933 | 2.7122 | 1.733 | 1.4028 | MDP0000598183 | 1.8534 |  |  |
| MDP0000125609 |  | 1.6843 | 2.1233 | MDP0000208403 | 1.3753 |  |  | MDP0000286949 |  |  | 2.8266 | MDP0000598249 |  | 1.215 |  |
| MDP0000125814 | -1.9438 |  |  | MDP0000208491 | 1.9377 |  |  | MDP0000287017 | 1.2529 |  |  | MDP0000598822 |  |  | 1.0316 |
| MDP0000125950 | 1.0598 |  |  | MDP0000208497 |  | 1.1507 |  | MDP0000287293 | 1.7331 |  |  | MDP0000600063 | 3.837 |  |  |
| MDP0000126070 |  | 1.2369 |  | MDP0000208592 |  | 2.2983 |  | MDP0000287581 |  | -3.2551 |  | MDP0000600199 | -1.9977 |  |  |
| MDP0000126080 | 1.0042 |  |  | MDP0000208667 |  | -1.1439 |  | MDP0000287919 |  | -1.1974 |  | MDP0000602301 | -2.0708 |  |  |
| MDP0000126194 | -1.0444 |  |  | MDP0000209143 |  |  | 1.0048 | MDP0000287992 | 1.7448 |  |  | MDP0000602757 |  | 1.7929 |  |
| MDP0000126245 |  | -2.4998 |  | MDP0000209147 |  | 2.1907 |  | MDP0000288395 | -1.1328 |  |  | MDP0000604285 |  |  | 1.0594 |
| MDP0000126295 |  | -3.3822 |  | MDP0000209440 |  |  | 1.0208 | MDP0000288486 | 3.0768 |  |  | MDP0000605874 |  | 1.96 |  |
| MDP0000126335 | -1.1379 |  |  | MDP0000209633 | -2.5263 |  |  | MDP0000288729 | 1.3767 |  |  | MDP0000606021 |  | -1.5927 |  |
| MDP0000126481 |  |  | -1.5498 | MDP0000209713 |  | -1.7056 |  | MDP0000288768 |  |  | 1.0958 | MDP0000607654 | -1.7486 |  |  |
| MDP0000126567 | 3.9541 | 2.1826 | 1.5602 | MDP0000210012 |  | 1.0694 |  | MDP0000289110 | 1.1804 |  |  | MDP0000607785 | -1.275 | -1.1368 |  |
| MDP0000126693 |  |  | 1.2486 | MDP0000210077 | 1.2412 | 1.9518 | 1.2311 | MDP0000289303 |  | 3.1291 |  | MDP0000608081 |  | 1.122 |  |
| MDP0000126873 | -1.8775 | -2.9369 |  | MDP0000210218 |  | -2.6328 |  | MDP0000289414 | 1.542 |  |  | MDP0000609114 | -1.0499 |  |  |
| MDP0000126946 |  |  | 1.1971 | MDP0000210279 | -1.3409 |  |  | MDP0000289536 | 1.3278 | 1.1243 |  | MDP0000609235 | 1.2492 |  |  |
| MDP0000127009 | 1.0752 |  |  | MDP0000210534 |  | -1.5724 |  | MDP0000290028 |  |  | 2.2599 | MDP0000609318 | 1.0708 |  |  |
| MDP0000127023 | 1.5957 |  |  | MDP0000210654 |  |  | -2.9712 | MDP0000290071 |  | -1.1057 |  | MDP0000610961 | -2.2617 | -2.2489 |  |
| MDP0000127588 | 1.1484 |  |  | MDP0000210851 | 1.6981 |  |  | MDP0000290143 |  | 1.6905 | 1.076 | MDP0000611897 |  | 1.5833 |  |
| MDP0000127630 |  |  | -2.83 | MDP0000210917 |  |  | 1.4285 | MDP0000290211 | -1.2183 |  |  | MDP0000612660 |  | -1.2048 |  |
| MDP0000127633 | 1.2961 |  |  | MDP0000210939 |  |  | -1.0322 | MDP0000290295 |  |  | -1.4215 | MDP0000613143 | 1.8615 |  |  |
| MDP0000127691 | 2.1429 | 1.8664 |  | MDP0000211186 | 1.3418 |  |  | MDP0000290422 | 2.7878 | 1.7229 |  | MDP0000613561 |  | -1.0247 |  |
| MDP0000127732 |  | -1.0393 |  | MDP0000211202 | 1.0998 |  |  | MDP0000290601 | -2.5467 |  |  | MDP0000617077 | 1.9841 |  |  |
| MDP0000127757 |  |  | 1.3341 | MDP0000211411 | -2.4417 | -1.4475 |  | MDP0000290646 | 2.0669 |  |  | MDP0000617479 | 1.7388 |  |  |
| MDP0000127834 |  |  | -1.4399 | MDP0000211516 |  | -1.8132 |  | MDP0000291076 | 1.5712 |  |  | MDP0000618087 |  |  | 1.2757 |
| MDP0000127858 | -1.6514 | -1.535 |  | MDP0000211698 | -2.0635 |  |  | MDP0000291081 |  | -2.6953 | -1.6562 | MDP0000619503 |  |  | 1.0876 |
| MDP0000128089 | -2.4779 | -1.5307 | -1.9957 | MDP0000211848 |  | 1.051 |  | MDP0000291544 | -1.7062 | -1.0951 |  | MDP0000620944 | -1.0897 |  |  |
| MDP0000128161 |  | -3.5298 |  | MDP0000212002 |  | -2.388 |  | MDP0000292031 |  |  | -1.4041 | MDP0000622919 |  | 1.5317 |  |
| MDP0000128701 | -4.1006 | -3.2792 |  | MDP0000212156 |  | -1.0871 |  | MDP0000292132 | -4.2097 |  |  | MDP0000622920 |  | 1.5813 |  |
| MDP0000128723 |  | 1.6652 | 4.0447 | MDP0000212424 | 1.1631 |  |  | MDP0000292164 |  | -1.3897 |  | MDP0000624125 |  | -1.2283 |  |
| MDP0000128857 |  |  | 1.6873 | MDP0000212502 |  | 1.9216 |  | MDP0000292206 | 1.2684 |  | -1.2584 | MDP0000629767 |  |  | -1.0935 |
| MDP0000128910 | 1.4129 |  | 1.3329 | MDP0000212760 | 3.2547 |  |  | MDP0000292432 | -1.1473 |  |  | MDP0000629985 |  | 1.689 |  |
| MDP0000128951 | 2.0501 |  |  | MDP0000213002 |  |  | 1.0717 | MDP0000292492 |  | 1.2384 |  | MDP0000630439 |  | -1.8385 |  |
| MDP0000128968 |  |  | 1.6966 | MDP0000213638 |  |  | 1.1672 | MDP0000292908 |  | -2.2814 |  | MDP0000630746 |  |  | 1.6609 |
| MDP0000129470 | 1.1678 |  |  | MDP0000213770 | 1.1469 | 1.4702 |  | MDP0000293002 | -2.3708 |  | -1.0826 | MDP0000633623 |  | -1.9858 |  |
| MDP0000129505 | -1.6374 |  |  | MDP0000214532 |  | -3.3781 |  | MDP0000293045 | 1.4159 | 1.4202 |  | MDP0000633759 |  |  | -1.4349 |
| MDP0000130212 |  |  | 1.1398 | MDP0000214814 |  | 4.8546 |  | MDP0000293087 | -1.1123 |  |  | MDP0000634676 |  |  | -2.1025 |
| MDP0000130368 |  |  | -4.7265 | MDP0000215167 |  | 1.4496 |  | MDP0000293119 |  |  | 2.9627 | MDP0000635733 | 1.1849 |  |  |
| MDP0000130459 |  |  | 1.3914 | MDP0000215328 |  | 1.0934 |  | MDP0000293214 | -2.2646 |  |  | MDP0000635740 |  |  | -1.1832 |
| MDP0000130467 |  | 1.0315 |  | MDP0000215367 |  |  | -1.4418 | MDP0000293232 | 1.0088 |  |  | MDP0000636593 | -2.263 | -2.3066 |  |
| MDP0000131142 | -1.9543 | -1.6723 |  | MDP0000215478 | -4.0765 |  |  | MDP0000293578 | 3.3881 | 2.288 | 2.2147 | MDP0000636876 |  |  | -1.9446 |
| MDP0000131267 | 1.3691 | 1.24 |  | MDP0000215585 | 1.385 |  | -1.5494 | MDP0000293642 | -2.0308 |  |  | MDP0000636927 | 4.6062 |  |  |
| MDP0000131322 |  |  | 1.6835 | MDP0000215611 | 1.0238 |  | -1.1061 | MDP0000294334 |  |  | 1.0758 | MDP0000639264 | 1.4305 |  |  |
| MDP0000131365 |  | -1.9929 |  | MDP0000216164 |  | -2.4762 |  | MDP0000294360 | -1.1205 |  |  | MDP0000640906 | -1.264 |  |  |
| MDP0000131529 |  | 1.5981 |  | MDP0000216225 |  |  | 1.4926 | MDP0000294365 |  | -1.5428 |  | MDP0000642253 | -2.3793 |  |  |
| MDP0000131617 | 1.1002 |  |  | MDP0000216487 | -1.0899 |  |  | MDP0000294444 | 3.2648 |  |  | MDP0000642530 | 1.5359 |  |  |
| MDP0000131763 | -1.8036 |  |  | MDP0000216517 |  | -2.5322 |  | MDP0000294568 | -1.8919 |  |  | MDP0000642594 | -1.1179 |  |  |
| MDP0000131964 |  | 1.2197 |  | MDP0000216647 |  | 1.1228 |  | MDP0000294667 | 3.3065 | 2.6168 | 1.5623 | MDP0000642826 | 1.6206 |  |  |
| MDP0000132207 |  |  | 1.3103 | MDP0000216786 |  |  | -2.4235 | MDP0000295258 |  |  | 1.159 | MDP0000644797 |  | 1.1775 |  |
| MDP0000132209 |  |  | 1.2976 | MDP0000216812 | 1.3088 |  |  | MDP0000295390 |  | 1.7146 |  | MDP0000646125 | 1.4594 | 1.4204 |  |
| MDP0000132346 |  | 1.286 |  | MDP0000217043 | 1.1522 |  | -1.0014 | MDP0000295392 | 1.926 |  |  | MDP0000647263 |  | 1.4258 |  |
| MDP0000132431 |  | 2.0503 |  | MDP0000217090 |  |  | -1.6568 | MDP0000295562 | 1.1178 |  |  | MDP0000647373 |  |  | -2.8492 |
| MDP0000132527 | -1.8161 |  |  | MDP0000217124 | -1.4131 |  |  | MDP0000295794 |  | -1.1941 |  | MDP0000649022 |  | -1.6556 |  |
| MDP0000133203 |  | -1.3589 |  | MDP0000217406 | 1.0336 |  |  | MDP0000295959 | 3.1642 |  |  | MDP0000649866 |  | -1.7753 |  |
| MDP0000133262 | -1.9757 |  |  | MDP0000217423 | -1.371 | -1.155 |  | MDP0000296000 | 2.3455 |  |  | MDP0000650358 | -1.3095 |  |  |
| MDP0000133416 |  | 1.2962 |  | MDP0000217508 |  | 1.2733 |  | MDP0000296317 |  | -1.5355 |  | MDP0000651353 |  | -1.932 |  |
| MDP0000133622 | 1.1279 |  |  | MDP0000217690 | -1.3 |  |  | MDP0000296357 |  |  | 2.2703 | MDP0000652004 |  | 1.7668 | 1.5987 |
| MDP0000134325 |  |  | 1.7188 | MDP0000217946 |  | 2.4054 |  | MDP0000296410 |  |  | 1.3176 | MDP0000654314 | -3.6998 | -5.8516 |  |
| MDP0000134377 |  |  | 1.4031 | MDP0000218302 | 1.7812 |  |  | MDP0000296468 | 1.6301 |  |  | MDP0000654738 |  |  | 1.3376 |
| MDP0000134621 |  | -2.3962 | -1.4603 | MDP0000218420 |  |  | 1.3537 | MDP0000296673 |  |  | 1.1163 | MDP0000654783 |  |  | -1.2614 |
| MDP0000134728 | 1.1302 |  |  | MDP0000218462 |  | 1.4068 |  | MDP0000296953 | 2.323 |  |  | MDP0000655330 | 2.321 |  |  |
| MDP0000134742 |  |  | 1.4546 | MDP0000218549 |  | 1.0348 |  | MDP0000297138 |  |  | -1.2304 | MDP0000656197 |  |  | 1.4051 |
| MDP0000134791 | 2.5431 | 1.5105 |  | MDP0000218748 | -2.8636 | -2.1491 |  | MDP0000297646 | 2.0115 | 1.3056 | 1.9142 | MDP0000656802 | -1.8061 |  |  |
| MDP0000134994 |  |  | 1.3558 | MDP0000218810 | 1.9557 |  |  | MDP0000298597 | 1.0808 |  |  | MDP0000657852 |  | -1.9956 |  |
| MDP0000135062 |  | -2.38 |  | MDP0000218824 | -2.6724 |  |  | MDP0000298615 | 1.4005 |  |  | MDP0000657945 |  | -1.0346 |  |
| MDP0000135937 |  |  | -1.0383 | MDP0000219042 |  |  | 1.5601 | MDP0000298689 | -1.32 |  |  | MDP0000658479 | -4.1311 |  |  |
| MDP0000136248 |  | 1.1163 |  | MDP0000219072 |  | -1.6734 |  | MDP0000299430 | 1.2565 |  |  | MDP0000659853 | -1.5336 |  |  |
| MDP0000136295 |  |  | -1.3676 | MDP0000219268 | -1.0246 |  |  | MDP0000299479 | 1.227 |  |  | MDP0000661908 |  |  | 1.4333 |
| MDP0000136345 |  | 1.0887 |  | MDP0000219285 |  | 1.0851 |  | MDP0000299555 | -5.1399 |  |  | MDP0000663057 | -1.3758 |  |  |
| MDP0000136398 |  | 1.1091 |  | MDP0000219684 |  | -1.3163 |  | MDP0000299693 |  | 1.1209 |  | MDP0000666415 | 1.1729 |  |  |
| MDP0000136500 |  |  | 1.044 | MDP0000219689 |  |  | 1.046 | MDP0000300051 |  |  | 4.7558 | MDP0000667895 |  | 1.0901 |  |
| MDP0000136609 |  | 2.6039 |  | MDP0000219838 |  |  | 1.5435 | MDP0000300139 | 1.0341 |  |  | MDP0000668041 | -1.2161 |  |  |
| MDP0000137221 | 1.2523 |  | 1.5799 | MDP0000220114 |  |  | 1.0733 | MDP0000300384 | 1.0329 |  |  | MDP0000669437 | -1.5841 |  |  |
| MDP0000137225 | -1.0722 |  |  | MDP0000220160 |  |  | 2.1744 | MDP0000300808 |  |  | -1.0289 | MDP0000672726 | -1.4941 | -1.4304 |  |
| MDP0000137305 | -1.6105 |  |  | MDP0000220179 |  | -2.0114 |  | MDP0000300987 |  | -1.1552 |  | MDP0000673070 |  |  | -2.2908 |
| MDP0000137551 | 1.3397 |  |  | MDP0000220480 | -1.558 | -1.3277 |  | MDP0000301020 |  | 1.326 |  | MDP0000674141 | -1.6805 |  |  |
| MDP0000137969 |  | 1.3935 |  | MDP0000220876 | 2.5598 |  |  | MDP0000301199 | -1.1652 |  |  | MDP0000674377 |  | 1.0125 |  |
| MDP0000138061 | -1.924 | -1.9281 |  | MDP0000221292 |  | 3.3879 |  | MDP0000301521 | -2.5449 |  |  | MDP0000676670 |  | -1.2334 |  |
| MDP0000138581 | 1.1997 |  |  | MDP0000221363 | -1.0074 |  |  | MDP0000301576 |  | 1.9118 |  | MDP0000679993 |  | 1.3537 |  |
| MDP0000138655 |  |  | 2.5887 | MDP0000221435 | 1.1596 |  |  | MDP0000301606 | 1.1651 |  |  | MDP0000680997 |  |  | 1.2718 |
| MDP0000138668 | 1.6321 |  |  | MDP0000222184 |  | -1.5042 |  | MDP0000301828 |  | 1.4045 |  | MDP0000681060 | -2.1801 |  |  |
| MDP0000138826 | 7.1664 |  |  | MDP0000222186 |  | -1.035 |  | MDP0000301958 | -1.6782 |  |  | MDP0000681131 | 2.2035 |  |  |
| MDP0000138855 |  | 1.1711 |  | MDP0000222189 |  |  | -1.4117 | MDP0000302024 | 2.3899 |  | 1.5855 | MDP0000681201 | 1.092 |  |  |
| MDP0000139219 | -1.3051 |  |  | MDP0000222305 |  |  | 1.056 | MDP0000302115 |  | 2.4927 |  | MDP0000681936 | 2.0296 |  | 1.2817 |
| MDP0000139221 |  |  | -1.0256 | MDP0000222555 |  |  | 1.2988 | MDP0000302222 | -1.1748 |  |  | MDP0000684133 | -2.7587 |  |  |
| MDP0000139278 |  |  | -3.6768 | MDP0000222633 | 1.3516 |  |  | MDP0000302399 | -1.1073 |  |  | MDP0000686661 | 3.5637 | 2.4883 | 1.7821 |
| MDP0000139525 |  | 2.658 |  | MDP0000222681 | 1.2236 |  |  | MDP0000302467 |  |  | -1.0075 | MDP0000686666 | 3.4945 | 2.1681 | 2.1387 |
| MDP0000139736 |  |  | -1.0232 | MDP0000222706 | -1.4778 | -1.7025 |  | MDP0000303216 | 2.8552 |  |  | MDP0000686885 |  |  | 1.0285 |
| MDP0000139821 | -1.7086 | -2.4066 |  | MDP0000222724 | 2.0868 |  |  | MDP0000303291 | 1.1857 |  |  | MDP0000687738 | -1.6973 |  |  |
| MDP0000139847 |  |  | -1.3755 | MDP0000222913 |  |  | 1.2139 | MDP0000303333 | -4.4736 |  |  | MDP0000688415 | 2.3802 | 2.1227 | 1.9103 |
| MDP0000139875 |  | 1.1817 |  | MDP0000223122 | -1.4717 |  |  | MDP0000303739 |  |  | 1.0534 | MDP0000688643 |  |  | -1.0114 |
| MDP0000140078 |  |  | 1.3886 | MDP0000223309 |  |  | 1.0814 | MDP0000303786 | -2.132 |  |  | MDP0000689386 | -1.1235 | -1.0393 |  |
| MDP0000140621 | 2.7775 |  |  | MDP0000223519 | 1.2903 |  |  | MDP0000303799 | 1.8925 |  |  | MDP0000689477 | -1.6569 |  |  |
| MDP0000140815 | -2.616 |  |  | MDP0000223568 |  | 1.7331 |  | MDP0000303818 | 3.3789 |  |  | MDP0000690108 |  | 1.3071 |  |
| MDP0000141719 | 1.584 | 1.3344 |  | MDP0000223631 | 1.519 |  |  | MDP0000304131 |  | -2.3635 |  | MDP0000691789 | 1.1739 |  |  |
| MDP0000141915 |  | 1.8789 |  | MDP0000223787 | 1.1909 |  |  | MDP0000304369 |  | -3.4069 |  | MDP0000693678 |  | -2.1897 |  |
| MDP0000142164 |  | -2.768 |  | MDP0000224423 |  | 1.0036 |  | MDP0000304842 |  | 1.2933 |  | MDP0000694227 |  | 1.0898 |  |
| MDP0000142206 |  |  | 1.0396 | MDP0000224533 |  |  | -1.5044 | MDP0000305017 |  |  | 1.7942 | MDP0000695737 | -2.591 |  |  |
| MDP0000142524 | 2.5274 |  |  | MDP0000224545 |  |  | 1.6828 | MDP0000305345 |  | 1.0158 |  | MDP0000696333 | -4.6754 |  |  |
| MDP0000142739 |  | -1.1257 |  | MDP0000224741 |  | -1.7097 |  | MDP0000305369 | 1.8053 |  |  | MDP0000697285 | 1.3255 | 1.004 |  |
| MDP0000142893 | 1.499 |  |  | MDP0000224773 | -1.6197 |  |  | MDP0000305731 | 1.266 |  |  | MDP0000697620 |  |  | 1.3253 |
| MDP0000143677 |  |  | -1.0196 | MDP0000225088 |  |  | -1.3305 | MDP0000305934 |  | 2.2591 |  | MDP0000697638 | 1.4435 |  |  |
| MDP0000144150 | 1.691 |  |  | MDP0000225491 | 3.0211 |  |  | MDP0000306888 |  | -1.7321 |  | MDP0000698220 |  | -1.9299 |  |
| MDP0000144280 |  | -1.5074 |  | MDP0000225680 | 2.5671 | 1.4825 | 1.1364 | MDP0000307173 |  |  | -6.6119 | MDP0000699531 | 1.7552 |  |  |
| MDP0000144481 | -1.139 | -1.0502 |  | MDP0000225793 | -1.0245 |  |  | MDP0000307703 |  |  | 1.0949 | MDP0000700267 | -2.8831 |  |  |
| MDP0000144558 |  |  | -1.0874 | MDP0000226223 |  | -1.1389 |  | MDP0000307728 |  |  | 1.6827 | MDP0000702868 | -1.0884 |  |  |
| MDP0000144734 |  |  | 1.407 | MDP0000226787 | 1.316 |  |  | MDP0000307853 | -2.3379 |  |  | MDP0000703247 | -1.0686 |  |  |
| MDP0000145036 |  | 1.0114 |  | MDP0000226967 | 1.2679 |  |  | MDP0000307964 |  |  | 1.1498 | MDP0000704196 |  |  | -1.3521 |
| MDP0000145279 | -1.6569 |  |  | MDP0000227119 | -2.9015 | -1.2075 |  | MDP0000308285 | 1.257 | 1.7732 |  | MDP0000706020 | 2.3175 |  |  |
| MDP0000145335 |  |  | 1.3474 | MDP0000227335 |  | 1.1733 |  | MDP0000308419 | 1.2881 |  |  | MDP0000710349 |  | -1.1311 |  |
| MDP0000145448 |  | -1.0909 |  | MDP0000227623 |  | -2.8088 |  | MDP0000309059 |  |  | -1.4981 | MDP0000710522 | -3.0174 |  |  |
| MDP0000145643 |  | 1.1419 |  | MDP0000228529 |  | -1.7409 |  | MDP0000309093 | 1.2205 |  |  | MDP0000711832 | -1.5483 |  |  |
| MDP0000145972 |  | -1.3603 | -1.1627 | MDP0000228662 |  |  | 1.6672 | MDP0000309192 |  | 1.1773 |  | MDP0000711891 | -1.5202 |  |  |
| MDP0000146033 |  |  | 1.0423 | MDP0000228839 |  |  | -1.2972 | MDP0000309291 | 2.3289 |  |  | MDP0000714688 |  |  | 1.105 |
| MDP0000146360 |  |  | -1.4573 | MDP0000229726 | -2.1604 | -1.6584 |  | MDP0000309383 |  |  | -1.1531 | MDP0000716151 | 1.3431 |  |  |
| MDP0000146449 |  | -1.1641 |  | MDP0000230194 |  |  | 2.1345 | MDP0000309451 | 1.345 |  |  | MDP0000716238 |  | 1.7353 |  |
| MDP0000146639 |  |  | -1.0333 | MDP0000230223 |  | 1.333 |  | MDP0000309756 |  |  | 1.3421 | MDP0000716986 | 1.0875 | 1.5558 | 1.5235 |
| MDP0000146863 |  | -1.0236 |  | MDP0000230265 | 3.0607 |  |  | MDP0000310001 | -1.4499 |  |  | MDP0000721987 | 1.1537 | 1.0286 |  |
| MDP0000147216 | 2.411 | 1.6015 | 1.9954 | MDP0000230429 | 2.167 |  |  | MDP0000310094 |  |  | -1.3404 | MDP0000723447 | -1.2721 |  |  |
| MDP0000147232 | -2.1165 |  |  | MDP0000230433 |  |  | 1.1454 | MDP0000310866 | 1.127 |  |  | MDP0000724659 |  |  | 1.4308 |
| MDP0000147368 | 4.3582 |  |  | MDP0000230638 | 1.0769 | 1.0136 |  | MDP0000310948 | -2.7245 |  |  | MDP0000727570 | -2.6303 | -1.6876 |  |
| MDP0000147596 | 3.7006 |  |  | MDP0000230836 | -1.2639 |  |  | MDP0000311338 |  | 2.4496 |  | MDP0000727860 |  | 1.0792 |  |
| MDP0000147913 | 2.0115 |  |  | MDP0000231093 |  | -2.8865 |  | MDP0000311922 | -2.5466 |  |  | MDP0000729348 | -1.2122 |  |  |
| MDP0000147996 | -2.6782 |  |  | MDP0000231376 | 1.2221 |  |  | MDP0000312212 |  |  | -2.7051 | MDP0000729533 | -1.5348 |  |  |
| MDP0000148169 | 1.7683 |  |  | MDP0000231390 |  |  | -1.7816 | MDP0000312565 | 3.8395 |  |  | MDP0000732635 | -1.1588 |  |  |
| MDP0000148287 |  |  | 1.1188 | MDP0000231417 |  |  | -2.3616 | MDP0000312665 |  | -1.5673 |  | MDP0000734535 | 1.3246 |  |  |
| MDP0000148545 | 1.9432 |  |  | MDP0000231545 |  | 1.3295 |  | MDP0000312701 | -1.9928 |  |  | MDP0000738420 |  | 2.0497 |  |
| MDP0000148686 | 1.2723 |  |  | MDP0000231668 | -1.1771 |  |  | MDP0000313434 |  | 1.3233 |  | MDP0000738653 | 1.3489 |  |  |
| MDP0000148815 |  |  | -1.22 | MDP0000231935 |  | -5.6937 |  | MDP0000313485 |  | 4.1057 |  | MDP0000741963 | 1.0844 |  |  |
| MDP0000148873 |  |  | 1.1825 | MDP0000232035 |  | -2.9067 |  | MDP0000313559 |  |  | 1.0887 | MDP0000744397 |  |  | 1.3003 |
| MDP0000149317 | -1.1846 |  |  | MDP0000232050 |  |  | 1.1208 | MDP0000313603 |  |  | -1.0639 | MDP0000747755 | -2.1537 |  |  |
| MDP0000149570 | 2.7229 |  |  | MDP0000232292 |  |  | 1.0323 | MDP0000313762 | 1.8864 |  |  | MDP0000750556 | -1.2034 |  |  |
| MDP0000149653 |  |  | 1.7442 | MDP0000232330 | 1.2985 |  |  | MDP0000314223 |  |  | -1.1445 | MDP0000751192 | -1.0176 |  |  |
| MDP0000149835 | -1.2428 |  |  | MDP0000232410 | 1.3233 |  | -1.1171 | MDP0000314595 | 2.733 |  |  | MDP0000753748 |  |  | 1.1931 |
| MDP0000149947 |  |  | 1.1737 | MDP0000232589 | 1.1149 | 1.15 |  | MDP0000314984 | -1.7245 |  |  | MDP0000754054 |  |  | 1.0587 |
| MDP0000150049 | 1.478 |  |  | MDP0000232662 |  |  | 1.0385 | MDP0000315320 |  | -1.2901 |  | MDP0000754356 | 2.0696 |  |  |
| MDP0000150108 | 1.1142 |  |  | MDP0000232941 |  |  | 1.2909 | MDP0000315378 | -1.7478 |  |  | MDP0000754445 | 1.0639 |  |  |
| MDP0000150235 |  |  | 1.4539 | MDP0000232957 | -1.2066 |  |  | MDP0000315480 | 1.0076 |  |  | MDP0000754989 | -2.3291 |  |  |
| MDP0000150279 |  |  | -1.391 | MDP0000232990 | 1.184 |  |  | MDP0000315482 | 1.8885 |  |  | MDP0000755899 |  | 1.1154 |  |
| MDP0000151202 | 1.0702 |  |  | MDP0000233037 | 1.1773 | 1.0506 |  | MDP0000315483 |  |  | 1.0003 | MDP0000757585 | -1.2156 |  |  |
| MDP0000151537 | -1.0005 |  |  | MDP0000233070 |  |  | -1.4376 | MDP0000316256 |  |  | 1.3378 | MDP0000759336 | 2.4107 | 1.2268 |  |
| MDP0000151618 | 1.4666 |  |  | MDP0000233235 | 1.8308 |  |  | MDP0000317061 |  |  | -1.4041 | MDP0000759646 | -2.6173 |  |  |
| MDP0000151767 |  |  | 1.9817 | MDP0000233303 | -1.7591 |  |  | MDP0000317257 | 2.077 |  |  | MDP0000759988 | 1.1125 |  |  |
| MDP0000152369 | 1.2643 |  |  | MDP0000233409 | 1.0104 |  |  | MDP0000317366 |  |  | -1.3056 | MDP0000760994 |  |  | 1.4766 |
| MDP0000152497 |  | -2.5359 |  | MDP0000233546 |  |  | 1.1417 | MDP0000317906 | 1.0857 |  |  | MDP0000761511 | 1.2305 |  |  |
| MDP0000152821 | 1.0324 | 1.19 |  | MDP0000233667 | 1.4887 |  |  | MDP0000317932 |  |  | -2.8924 | MDP0000762227 |  |  | 1.5636 |
| MDP0000153063 | 2.2941 | 2.2439 |  | MDP0000233668 | -1.3109 |  |  | MDP0000317970 | 1.2551 |  |  | MDP0000764712 |  | 1.0404 |  |
| MDP0000153069 |  |  | 1.1906 | MDP0000233691 | 1.4051 |  | 1.9302 | MDP0000318040 |  | 1.2053 |  | MDP0000764760 |  | 1.4929 |  |
| MDP0000153382 | 1.0838 | 1.1245 | 1.0425 | MDP0000233737 | 3.2319 |  |  | MDP0000318130 | 1.2779 |  |  | MDP0000767063 | -2.244 | -1.1876 |  |
| MDP0000153419 |  |  | 1.5393 | MDP0000234215 |  |  | 1.3004 | MDP0000318732 |  | -1.6637 |  | MDP0000768176 |  |  | 5.5127 |
| MDP0000153539 | -2.5932 |  |  | MDP0000234244 | 1.5008 |  |  | MDP0000318866 |  |  | -1.3422 | MDP0000768369 | -3.8803 |  |  |
| MDP0000153630 | 4.0411 |  |  | MDP0000234291 |  |  | -1.2838 | MDP0000318891 | 1.352 | 1.0486 |  | MDP0000768370 |  |  | -1.5868 |
| MDP0000153807 | 1.3518 |  |  | MDP0000234528 |  | -1.1202 |  | MDP0000318900 |  |  | 2.522 | MDP0000770205 |  |  | -1.2327 |
| MDP0000154070 | 1.3678 |  | -1.554 | MDP0000234707 | 2.0794 |  |  | MDP0000319016 | 1.3349 |  |  | MDP0000770377 | -1.3267 |  |  |
| MDP0000154475 |  |  | 2.1267 | MDP0000234847 |  | 1.1288 |  | MDP0000319079 |  |  | -1.2322 | MDP0000771561 | 1.024 |  |  |
| MDP0000154838 | -3.0152 |  |  | MDP0000235023 |  |  | 1.1836 | MDP0000319132 | 1.0344 |  |  | MDP0000771567 | -1.1112 |  |  |
| MDP0000154855 | -2.9435 | -2.6938 |  | MDP0000235569 | 1.0384 |  |  | MDP0000319179 | 1.6167 | 1.7336 |  | MDP0000772420 | 3.3562 |  |  |
| MDP0000155158 | 1.5672 |  | -1.0655 | MDP0000235802 |  |  | 1.0761 | MDP0000319328 | 1.0927 |  |  | MDP0000772938 | -2.47 |  |  |
| MDP0000155293 |  |  | 2.0739 | MDP0000236072 | -1.2359 |  |  | MDP0000319451 |  |  | -1.4389 | MDP0000775970 | 1.0843 |  |  |
| MDP0000155446 |  | 2.0317 |  | MDP0000236081 |  |  | 1.3079 | MDP0000319460 |  |  | 1.0848 | MDP0000776040 | -1.7192 |  |  |
| MDP0000155579 | -1.1723 |  |  | MDP0000236150 | -1.3678 |  |  | MDP0000319502 |  |  | 1.5326 | MDP0000777336 | -1.3362 |  |  |
| MDP0000155715 | 1.6635 |  |  | MDP0000236614 |  |  | 1.6463 | MDP0000319744 |  | 1.6217 |  | MDP0000778201 |  |  | -2.2893 |
| MDP0000156088 |  | -1.8318 |  | MDP0000237039 | 1.1298 |  |  | MDP0000319966 |  | 1.3765 |  | MDP0000778861 |  |  | -1.7475 |
| MDP0000156131 |  |  | 1.4155 | MDP0000237150 | -3.2083 |  |  | MDP0000320406 | -1.6191 |  | -1.4063 | MDP0000780353 | -1.5654 |  |  |
| MDP0000156135 |  |  | 1.1198 | MDP0000237443 |  |  | -1.4132 | MDP0000320471 | 1.8285 |  |  | MDP0000780459 | 1.1216 |  |  |
| MDP0000156139 |  |  | 1.0897 | MDP0000237586 | 1.4018 |  |  | MDP0000320534 | 1.5232 |  |  | MDP0000781314 |  |  | -1.1028 |
| MDP0000156241 |  | 1.0992 |  | MDP0000237668 | 1.2713 |  |  | MDP0000320717 | 1.5304 |  | -1.0251 | MDP0000782085 |  | -2.0156 |  |
| MDP0000156351 |  | -1.6952 |  | MDP0000237742 | -1.9944 |  |  | MDP0000320783 | 1.3716 |  | -1.2798 | MDP0000782642 | 1.8827 |  |  |
| MDP0000156478 |  |  | 1.5949 | MDP0000237989 |  |  | -1.2147 | MDP0000320866 |  | -2.4139 |  | MDP0000784168 |  | -1.1374 |  |
| MDP0000156530 | -3.4199 | -2.7274 |  | MDP0000238382 |  | 1.8945 |  | MDP0000320910 |  | 1.1971 |  | MDP0000784187 |  | 1.1333 |  |
| MDP0000156548 | 1.1233 | 1.054 |  | MDP0000238675 |  |  | 1.0181 | MDP0000321018 |  |  | -1.2119 | MDP0000785966 | 1.2175 |  |  |
| MDP0000156580 | -1.4484 |  |  | MDP0000238940 |  | 1.1116 |  | MDP0000321031 | 1.3603 |  |  | MDP0000786540 | -1.8532 | -1.5863 |  |
| MDP0000156921 |  | -1.9035 |  | MDP0000238942 |  |  | 1.1473 | MDP0000321062 |  | 1.1047 |  | MDP0000786569 |  | -1.3519 |  |
| MDP0000157072 |  |  | 1.2727 | MDP0000239296 |  | 3.3904 |  | MDP0000321215 | 1.2521 |  |  | MDP0000786815 | 2.1851 |  |  |
| MDP0000157094 | 2.3493 |  | 1.2166 | MDP0000239381 |  |  | 1.0185 | MDP0000321577 | 2.0187 |  |  | MDP0000787216 |  | -1.0316 |  |
| MDP0000157124 | -2.8145 |  |  | MDP0000239530 |  |  | 1.8424 | MDP0000321850 | -1.0257 |  |  | MDP0000787808 |  | -2.1151 |  |
| MDP0000157170 | -1.5402 |  |  | MDP0000239646 | 1.0149 |  |  | MDP0000321910 | 2.4131 |  | 2.2924 | MDP0000787842 |  |  | 1.0919 |
| MDP0000157345 | 2.6787 |  |  | MDP0000239754 |  | -1.1976 |  | MDP0000321920 | 1.4937 |  |  | MDP0000787909 |  |  | 1.5921 |
| MDP0000157447 |  |  | 1.1653 | MDP0000239951 |  |  | -1.3138 | MDP0000321945 |  |  | 1.5608 | MDP0000788934 | 3.3429 | 2.1478 | 2.3962 |
| MDP0000157711 | -1.2513 | -1.1917 |  | MDP0000240094 |  |  | -3.4084 | MDP0000322202 |  |  | -1.133 | MDP0000790376 |  |  | -2.6436 |
| MDP0000157816 | 1.614 |  |  | MDP0000240315 |  |  | -1.2827 | MDP0000322261 |  |  | 1.35 | MDP0000790788 | 1.5377 |  |  |
| MDP0000157828 |  | 1.0451 |  | MDP0000240641 | 4.823 |  |  | MDP0000322416 |  | -1.6225 |  | MDP0000791177 | -2.8637 |  |  |
| MDP0000157932 |  | 1.0164 |  | MDP0000240643 | 2.8224 | 2.4704 | 2.1633 | MDP0000322504 |  |  | 1.2889 | MDP0000791184 |  |  | 1.1418 |
| MDP0000157962 |  | -1.1581 |  | MDP0000240651 |  | 2.5141 |  | MDP0000322563 |  |  | 1.1792 | MDP0000793247 |  |  | 1.1333 |
| MDP0000158047 | -1.9476 |  |  | MDP0000240735 | 1.2784 | 1.6036 |  | MDP0000322989 |  | -1.1982 |  | MDP0000793268 | 3.148 |  |  |
| MDP0000158057 |  |  | 2.6666 | MDP0000240781 | -1.5725 |  |  | MDP0000323263 |  |  | 1.3251 | MDP0000793896 | 1.1669 |  |  |
| MDP0000158520 |  | 1.0021 |  | MDP0000240888 |  |  | -2.261 | MDP0000323277 |  |  | -1.3363 | MDP0000794528 |  |  | 1.054 |
| MDP0000159011 | 2.081 |  |  | MDP0000241185 | 1.4961 | 1.396 |  | MDP0000323375 |  |  | 1.2523 | MDP0000795650 | 2.4742 |  |  |
| MDP0000159402 | 6.099 |  |  | MDP0000241199 | 2.4789 |  |  | MDP0000323864 | 1.8075 | 1.2016 | 1.3307 | MDP0000797782 |  |  | 1.9536 |
| MDP0000159583 |  | -1.1199 |  | MDP0000241392 |  |  | 1.0963 | MDP0000323950 | -2.1453 |  |  | MDP0000798156 |  | 1.2363 |  |
| MDP0000159721 | 1.6701 |  |  | MDP0000241444 |  |  | -1.3138 | MDP0000324249 |  | 1.4224 |  | MDP0000800945 |  |  | 1.0324 |
| MDP0000159766 |  | -1.8858 |  | MDP0000241455 |  | -1.9627 |  | MDP0000324681 | -1.4374 |  |  | MDP0000801117 | 1.0583 |  |  |
| MDP0000160289 | -2.1141 |  |  | MDP0000241847 |  | -3.3325 |  | MDP0000325497 | -1.3221 |  |  | MDP0000801659 |  |  | 1.1438 |
| MDP0000160384 | 4.596 |  |  | MDP0000242152 | 1.1348 |  |  | MDP0000325781 |  |  | 1.0276 | MDP0000803773 |  |  | 1.7767 |
| MDP0000160393 | 1.2776 |  |  | MDP0000242413 | -1.7759 |  |  | MDP0000325832 |  | 1.4881 |  | MDP0000803920 | -2.6552 |  |  |
| MDP0000161063 | -3.0256 |  |  | MDP0000242554 | -2.8142 |  |  | MDP0000326906 |  |  | 1.1185 | MDP0000805369 |  |  | 1.2128 |
| MDP0000161121 |  |  | 2.0552 | MDP0000242568 | 1.2482 |  |  | MDP0000327079 | -1.4104 |  |  | MDP0000807738 |  | -1.5793 |  |
| MDP0000161388 | -1.1351 |  |  | MDP0000242611 |  | 1.1299 |  | MDP0000328589 | 1.3626 |  |  | MDP0000808124 |  | -2.1795 |  |
| MDP0000162426 |  | 1.1551 |  | MDP0000242922 |  |  | 1.2845 | MDP0000329746 |  |  | 1.058 | MDP0000808334 | -2.4579 |  |  |
| MDP0000162904 | -1.1932 |  |  | MDP0000242926 | -1.2416 |  |  | MDP0000329863 | 2.2696 |  |  | MDP0000809488 |  |  | 1.2976 |
| MDP0000162922 |  | 1.084 |  | MDP0000243138 |  | 1.6364 |  | MDP0000330571 |  | 1.1895 |  | MDP0000809801 | 1.0637 | 1.4849 |  |
| MDP0000163007 |  | 1.3761 |  | MDP0000243375 | 1.021 |  |  | MDP0000331309 |  |  | -2.584 | MDP0000810488 |  |  | 1.0749 |
| MDP0000163032 | -1.4261 |  |  | MDP0000243380 | 1.8666 |  |  | MDP0000332388 | 1.0373 |  |  | MDP0000811127 |  | -1.3556 |  |
| MDP0000163088 | 1.086 |  |  | MDP0000243404 | -1.4064 |  |  | MDP0000332597 |  |  | -1.2883 | MDP0000813805 | -1.0161 |  |  |
| MDP0000163338 | -2.4143 |  |  | MDP0000243738 |  | 1.2399 |  | MDP0000332721 |  |  | -1.3728 | MDP0000816500 | 1.2615 | 1.0524 |  |
| MDP0000163588 | 1.7295 |  |  | MDP0000243940 | 1.1676 |  |  | MDP0000332899 |  |  | 1.4254 | MDP0000817733 | -1.9638 |  |  |
| MDP0000163811 | -2.603 |  |  | MDP0000244238 |  | 1.7714 |  | MDP0000334585 |  | 1.2067 |  | MDP0000818047 |  |  | -1.0706 |
| MDP0000164054 |  | 1.0736 |  | MDP0000244450 | 1.5312 |  |  | MDP0000336127 |  | -1.6583 |  | MDP0000819856 |  |  | 1.3103 |
| MDP0000164160 | -1.0877 |  |  | MDP0000244506 |  | 1.1928 |  | MDP0000336128 | 1.0869 |  |  | MDP0000820483 | 1.2014 |  |  |
| MDP0000164504 | 1.2384 |  |  | MDP0000244775 |  |  | 1.3668 | MDP0000336174 | 1.7731 |  |  | MDP0000821608 |  | 3.3772 |  |
| MDP0000164794 |  |  | -1.2161 | MDP0000245208 | -2.9578 |  |  | MDP0000336462 | 1.1727 |  |  | MDP0000821959 | 1.7798 | 1.5779 |  |
| MDP0000165031 | -1.7653 | -1.3498 |  | MDP0000245551 | 1.1364 |  |  | MDP0000340864 |  |  | 1.1566 | MDP0000822752 | -2.1089 |  |  |
| MDP0000165206 | 1.2693 |  |  | MDP0000245975 |  |  | 1.0389 | MDP0000342592 | -1.2848 |  |  | MDP0000822948 | 1.3403 |  |  |
| MDP0000165287 | 1.1342 |  |  | MDP0000246674 |  | -1.1999 |  | MDP0000342703 | -1.9848 |  |  | MDP0000824381 |  | -1.4681 |  |
| MDP0000165656 |  | 1.2001 | 1.3355 | MDP0000246957 | -1.6122 |  |  | MDP0000345066 |  | -2.3561 |  | MDP0000827881 | -1.0656 |  | -1.5807 |
| MDP0000165836 | 1.1203 |  |  | MDP0000247311 |  |  | 1.0472 | MDP0000345457 |  | 1.006 |  | MDP0000831501 | 1.0332 |  |  |
| MDP0000166068 |  | -2.4233 |  | MDP0000247698 | 2.2738 | 1.8227 |  | MDP0000346782 | 1.0737 | 1.1598 |  | MDP0000831734 |  |  | -2.1567 |
| MDP0000166359 | 1.5186 |  |  | MDP0000247868 |  | 1.2234 |  | MDP0000348036 | 2.9093 |  |  | MDP0000832066 |  | 2.459 |  |
| MDP0000166406 |  | 1.0589 |  | MDP0000247869 | 1.847 |  |  | MDP0000348067 |  | -1.9841 |  | MDP0000832104 |  | -5.3434 |  |
| MDP0000166587 |  |  | -1.4571 | MDP0000248043 | 1.1335 |  |  | MDP0000348372 | 1.4793 |  |  | MDP0000832105 | 1.5421 |  |  |
| MDP0000166644 |  | -1.368 | -1.4151 | MDP0000248100 | 1.1294 |  | -1.1155 | MDP0000350055 | 1.041 | 1.1955 |  | MDP0000832994 |  |  | 1.0492 |
| MDP0000166732 |  |  | 1.096 | MDP0000248312 |  | 1.0449 | 1.0157 | MDP0000350343 | 1.1415 | 1.2846 |  | MDP0000833178 |  |  | -1.1053 |
| MDP0000166916 | 1.175 |  |  | MDP0000248648 |  | 1.0222 |  | MDP0000355440 | -1.0633 |  |  | MDP0000834150 |  |  | -1.3699 |
| MDP0000167107 | -1.9114 |  |  | MDP0000248777 |  |  | -1.0029 | MDP0000356969 | -1.5552 |  |  | MDP0000835211 | -1.8138 |  |  |
| MDP0000167199 |  |  | 1.0316 | MDP0000248856 |  | 1.198 |  | MDP0000357231 |  | 2.096 |  | MDP0000836051 | -2.2362 |  |  |
| MDP0000167288 | -3.5627 |  |  | MDP0000248865 | 1.9481 |  |  | MDP0000360394 |  | -1.9023 |  | MDP0000838695 | 1.5639 |  |  |
| MDP0000167338 | -1.2162 |  |  | MDP0000249260 | -1.257 |  |  | MDP0000360414 | 2.1787 |  |  | MDP0000839207 |  | 1.0527 |  |
| MDP0000167387 |  |  | -1.3324 | MDP0000249427 | -1.8841 |  |  | MDP0000360438 |  | -1.6596 |  | MDP0000840330 | -3.0321 |  |  |
| MDP0000168026 | 1.0581 |  |  | MDP0000249714 |  |  | 1.7992 | MDP0000360447 |  | 2.3807 | 1.6841 | MDP0000840536 | 1.9546 | 1.6378 |  |
| MDP0000168556 | 3.4443 |  |  | MDP0000249986 | -1.4809 | -1.0887 |  | MDP0000361134 |  |  | 1.2038 | MDP0000841002 | 1.5512 |  | 1.2994 |
| MDP0000168714 | -1.0236 |  |  | MDP0000250118 | 1.2674 |  |  | MDP0000361449 | 1.2592 |  |  | MDP0000842520 |  |  | -1.3128 |
| MDP0000168972 |  | -1.1866 |  | MDP0000250440 | -1.6043 | -1.537 |  | MDP0000361589 | 1.3269 |  |  | MDP0000842702 | -1.2983 |  |  |
| MDP0000169059 |  | -1.9545 |  | MDP0000250519 |  |  | 1.0615 | MDP0000361644 |  | -1.2397 |  | MDP0000843015 |  |  | -1.2016 |
| MDP0000169311 |  | -1.5726 |  | MDP0000250602 | 1.265 |  |  | MDP0000361876 |  | 1.837 |  | MDP0000843137 |  | 1.5381 | 1.4903 |
| MDP0000170162 |  | -1.0909 |  | MDP0000250722 |  | 1.1561 |  | MDP0000364301 |  | -1.6448 |  | MDP0000843310 | 1.25 |  |  |
| MDP0000170286 |  |  | 1.0533 | MDP0000250866 | 1.8406 |  |  | MDP0000364547 | 1.3774 |  |  | MDP0000844719 | 2.2781 |  |  |
| MDP0000170603 |  |  | 1.3421 | MDP0000250895 |  |  | -1.1038 | MDP0000366412 | 3.7827 | 1.7265 | 2.4437 | MDP0000844941 | -1.5407 |  |  |
| MDP0000170822 |  |  | -1.7892 | MDP0000250932 |  | 1.096 |  | MDP0000366869 | -1.0394 |  |  | MDP0000846849 | 1.1634 |  |  |
| MDP0000171170 | 1.0244 |  |  | MDP0000250943 | -1.1372 |  |  | MDP0000368098 | -1.4392 |  |  | MDP0000846861 | 1.5276 |  |  |
| MDP0000171277 | 1.0159 |  |  | MDP0000251234 | 1.2809 |  |  | MDP0000368496 |  | 1.0234 |  | MDP0000850643 | -1.1694 | -1.7555 |  |
| MDP0000171695 |  | 2.77 |  | MDP0000251253 |  |  | 1.065 | MDP0000368719 |  | -1.7266 |  | MDP0000851342 |  |  | 2.4245 |
| MDP0000171707 |  |  | -1.0995 | MDP0000251398 |  | -3.316 |  | MDP0000369641 |  | 1.3488 |  | MDP0000854242 |  | 1.3188 |  |
| MDP0000171795 |  |  | 1.0494 | MDP0000251418 |  | 1.0595 | 1.7938 | MDP0000369704 | -1.2395 |  |  | MDP0000854432 |  | -1.4158 |  |
| MDP0000171825 | 1.1675 |  |  | MDP0000251419 |  |  | 2.8272 | MDP0000370825 |  | 1.1601 |  | MDP0000854541 |  |  | -1.9404 |
| MDP0000171955 | 1.0119 |  |  | MDP0000251424 |  |  | 1.191 | MDP0000374836 |  |  | 1.0295 | MDP0000854767 | 1.0674 |  |  |
| MDP0000172149 | 1.3612 |  |  | MDP0000251560 |  | -1.2606 |  | MDP0000377084 | 1.6334 |  |  | MDP0000854831 | 2.707 |  |  |
| MDP0000172356 | 1.5305 |  |  | MDP0000251783 | 1.4952 |  |  | MDP0000377788 | -1.1429 |  |  | MDP0000855442 | 1.1917 |  |  |
| MDP0000172376 | -1.4824 |  |  | MDP0000251865 | -1.7469 |  |  | MDP0000378203 |  | 1.4341 | -1.9386 | MDP0000856775 |  |  | 1.6165 |
| MDP0000172451 | -1.8271 |  |  | MDP0000251956 |  | 2.1789 |  | MDP0000381084 | 1.5322 |  |  | MDP0000857724 | 2.4328 |  |  |
| MDP0000172464 | -1.377 |  |  | MDP0000251967 | 1.7481 |  |  | MDP0000381669 |  |  | 1.0272 | MDP0000857733 | -2.9017 |  | -1.9224 |
| MDP0000172584 |  | -1.4042 |  | MDP0000252292 | 3.144 | 1.8226 | 2.207 | MDP0000383809 |  |  | 1.5395 | MDP0000860403 |  | -1.7172 |  |
| MDP0000173092 | -1.2684 |  |  | MDP0000252549 | -1.3663 | -1.3268 |  | MDP0000386723 | -2.1652 |  |  | MDP0000861303 |  |  | 1.2714 |
| MDP0000173095 | -1.5056 |  | 1.2661 | MDP0000252585 |  | 2.9025 |  | MDP0000386815 |  | -1.0458 |  | MDP0000864349 |  |  | 1.5651 |
| MDP0000173145 |  | -1.7743 |  | MDP0000252589 | 1.7776 | 1.175 | 1.2008 | MDP0000392154 | 1.2746 |  |  | MDP0000864817 |  | -1.1444 |  |
| MDP0000173235 | 2.6162 |  |  | MDP0000252680 |  |  | -1.4067 | MDP0000393227 |  | -1.7939 |  | MDP0000866270 | -2.5816 |  |  |
| MDP0000173300 |  |  | 1.0994 | MDP0000252819 |  |  | 1.26 | MDP0000393462 |  |  | 1.101 | MDP0000866840 | 3.7156 |  |  |
| MDP0000173534 | -4.7217 |  |  | MDP0000252967 |  | -2.1639 |  | MDP0000394847 |  | 3.1059 |  | MDP0000868045 |  | -1.8512 |  |
| MDP0000173611 |  | -1.148 |  | MDP0000252999 | 1.0508 |  |  | MDP0000394944 | 1.7502 |  |  | MDP0000868556 |  |  | -2.0948 |
| MDP0000173635 |  | 1.1553 |  | MDP0000253047 |  | -1.0491 |  | MDP0000396450 | -1.1509 |  |  | MDP0000869097 | -1.5494 | -1.2237 |  |
| MDP0000173694 | -1.5384 |  |  | MDP0000253113 |  | 1.4286 |  | MDP0000397367 |  |  | 1.0855 | MDP0000869725 | 1.0078 |  | 1.0277 |
| MDP0000174018 |  | -1.72 |  | MDP0000253189 | -1.8962 | -2.3645 |  | MDP0000398010 | 1.78 |  |  | MDP0000870405 | 1.0168 | 1.0404 |  |
| MDP0000174090 | -1.4456 |  |  | MDP0000253406 |  | -1.4862 |  | MDP0000400108 |  |  | -1.2004 | MDP0000870909 |  |  | 1.5385 |
| MDP0000174161 |  |  | 1.2261 | MDP0000253708 |  | 1.406 |  | MDP0000401034 |  | 1.0086 |  | MDP0000871602 |  |  | -1.3558 |
| MDP0000174496 | -4.3343 |  |  | MDP0000253735 |  | 1.2694 |  | MDP0000401049 |  |  | 1.5894 | MDP0000872262 | -2.0264 |  |  |
| MDP0000174501 |  |  | 1.4785 | MDP0000253785 |  | 1.2097 |  | MDP0000402656 | -2.028 |  |  | MDP0000873235 |  | -2.756 |  |
| MDP0000174532 |  | -3.8351 |  | MDP0000253809 | -1.9464 | -1.2495 |  | MDP0000403302 |  | -2.0619 |  | MDP0000873268 |  | 1.117 |  |
| MDP0000174597 | 1.4703 |  |  | MDP0000253860 | 1.159 |  |  | MDP0000404331 | -3.4999 |  |  | MDP0000873427 | 1.0061 |  |  |
| MDP0000174621 | 2.2804 |  |  | MDP0000254164 |  |  | -2.4644 | MDP0000404948 |  | 1.2082 |  | MDP0000874407 | 1.1267 |  |  |
| MDP0000175055 | 2.7644 |  |  | MDP0000254558 | 2.5121 |  |  | MDP0000405010 | 2.0227 |  |  | MDP0000874800 |  |  | -1.6724 |
| MDP0000175240 | -2.1928 |  |  | MDP0000254933 |  | 1.0343 |  | MDP0000405668 |  | -1.1604 |  | MDP0000876794 | 1.163 |  |  |
| MDP0000175375 |  |  | -2.9276 | MDP0000255044 | 1.243 |  | -1.4056 | MDP0000407273 |  |  | 2.6749 | MDP0000876872 |  |  | -3.8786 |
| MDP0000175821 |  | -2.1988 |  | MDP0000255080 |  |  | -4.2554 | MDP0000407613 |  |  | -1.5226 | MDP0000877084 |  | -3.2871 |  |
| MDP0000175918 |  |  | 1.1217 | MDP0000255146 |  |  | 2.057 | MDP0000410264 | 1.1484 |  |  | MDP0000878181 | -2.3615 | -1.6057 |  |
| MDP0000176234 | 2.6569 |  |  | MDP0000255534 |  |  | 1.1544 | MDP0000410696 | -2.3556 |  |  | MDP0000878790 | 1.3974 |  |  |
| MDP0000176374 |  |  | 1.0707 | MDP0000255711 |  | 1.3955 |  | MDP0000414607 |  | -2.3429 |  | MDP0000880312 |  | 1.4918 |  |
| MDP0000176956 |  |  | 1.0125 | MDP0000255974 |  |  | 1.176 | MDP0000415558 |  | -2.0717 |  | MDP0000880453 | 1.4833 |  |  |
| MDP0000177197 | 5.5156 |  |  | MDP0000256017 |  |  | -1.4973 | MDP0000416021 | -1.6643 | -1.3247 |  | MDP0000881763 |  | -1.0034 |  |
| MDP0000177244 | 1.6449 |  |  | MDP0000256199 |  | 1.5406 | 2.004 | MDP0000416118 |  |  | 1.0188 | MDP0000884644 |  |  | 1.1905 |
| MDP0000177906 | -1.1962 |  |  | MDP0000256413 | 1.0845 |  | -1.2843 | MDP0000417633 | 1.1562 |  |  | MDP0000885773 | 1.5529 |  |  |
| MDP0000178024 |  | -1.1182 |  | MDP0000256494 |  |  | 1.072 | MDP0000420871 |  | -2.1474 |  | MDP0000886138 | -1.4736 |  |  |
| MDP0000178042 | 1.7665 |  |  | MDP0000256522 | 1.694 |  |  | MDP0000421767 |  | 1.6108 |  | MDP0000886364 | -1.6512 |  |  |
| MDP0000178133 | -1.821 | -2.1998 |  | MDP0000256696 |  | -1.0832 |  | MDP0000424752 | 1.2675 |  | -1.5368 | MDP0000887107 | 1.6014 |  |  |
| MDP0000178245 |  |  | 1.2102 | MDP0000256697 |  |  | 1.3077 | MDP0000425375 |  |  | -1.2006 | MDP0000887768 | -3.1668 |  |  |
| MDP0000178529 |  | 1.2388 |  | MDP0000256805 |  |  | -1.0494 | MDP0000427753 | 1.264 |  |  | MDP0000888928 | 1.234 | 1.3662 | 1.6487 |
| MDP0000179121 | 1.015 |  |  | MDP0000256892 | -1.2368 |  |  | MDP0000428615 |  | -1.1043 |  | MDP0000889159 | 1.1627 | 1.5039 |  |
| MDP0000179301 | -1.4243 |  |  | MDP0000257814 |  | 1.386 |  | MDP0000429106 |  |  | 1.0411 | MDP0000889787 | -2.3749 |  |  |
| MDP0000179620 | 3.0072 |  |  | MDP0000258335 |  | 1.9771 |  | MDP0000429824 | 1.0476 |  | 1.4406 | MDP0000889955 |  | 1.406 |  |
| MDP0000179650 |  | 1.1955 |  | MDP0000258367 |  | -1.0866 |  | MDP0000430391 |  | 1.08 |  | MDP0000890206 | 1.4223 |  |  |
| MDP0000179654 | -1.5623 |  |  | MDP0000258414 |  |  | 1.0971 | MDP0000431417 |  | -2.0079 |  | MDP0000891532 |  |  | 1.0119 |
| MDP0000179747 |  | 1.1315 |  | MDP0000258428 |  | 1.4358 |  | MDP0000431696 | 2.2445 |  | 1.2147 | MDP0000891902 |  |  | 1.0593 |
| MDP0000179901 | 1.3908 |  | -1.338 | MDP0000258943 |  | -1.398 |  | MDP0000432128 | 1.6518 |  |  | MDP0000893240 |  | 2.0835 |  |
| MDP0000180376 |  |  | 1.1031 | MDP0000259057 | 1.4828 |  | -1.2119 | MDP0000432471 |  | -1.4361 |  | MDP0000893502 |  |  | -1.2648 |
| MDP0000180420 |  |  | 1.2798 | MDP0000259495 | 1.4359 |  |  | MDP0000433167 |  |  | -1.2856 | MDP0000893535 | -1.1402 |  |  |
| MDP0000180684 | -1.4366 | -1.6251 |  | MDP0000259614 |  | 2.5053 |  | MDP0000436064 |  |  | 1.3851 | MDP0000894176 | 1.2886 |  |  |
| MDP0000180974 |  |  | -2.7939 | MDP0000259621 |  |  | -1.2261 | MDP0000436155 |  | 1.0593 |  | MDP0000895175 |  |  | 1.1419 |
| MDP0000181436 | 3.0012 |  |  | MDP0000259632 |  |  | 2.2237 | MDP0000437664 | 3.6727 |  |  | MDP0000896759 |  |  | 1.4531 |
| MDP0000181884 | -2.2979 |  | -2.3988 | MDP0000259734 | 1.0632 |  |  | MDP0000438093 |  | -1.4443 |  | MDP0000897416 | -1.2211 |  |  |
| MDP0000182000 |  |  | 1.3796 | MDP0000259991 |  |  | -2.9948 | MDP0000439660 |  |  | -1.0644 | MDP0000898423 |  |  | 1.0544 |
| MDP0000182105 | 1.2652 |  |  | MDP0000260110 | -1.0042 |  |  | MDP0000440443 |  |  | -1.2678 | MDP0000899966 |  | 1.2098 |  |
| MDP0000182482 | 1.1593 |  |  | MDP0000260404 | 2.973 | 2.352 | 1.6298 | MDP0000442056 |  | -1.2312 |  | MDP0000901801 |  | -1.5962 |  |
| MDP0000182514 |  |  | 1.4774 | MDP0000260819 | 1.1203 |  |  | MDP0000442206 |  |  | 1.0127 | MDP0000902338 | 2.2403 |  | 1.6793 |
| MDP0000183140 |  | -1.4179 | -1.6508 | MDP0000261265 | 2.1913 | 1.6755 | 1.5728 | MDP0000442780 |  | -1.6207 |  | MDP0000903481 |  | 1.0241 |  |
| MDP0000183277 | -1.4865 |  |  | MDP0000261447 | 1.1913 |  |  | MDP0000443024 | 2.2765 |  |  | MDP0000907218 |  |  | 1.8642 |
| MDP0000183294 | 1.2871 |  |  | MDP0000261563 |  | 1.2464 |  | MDP0000444145 | -1.1239 |  |  | MDP0000907486 | -1.1104 | -1.1348 |  |
| MDP0000183470 |  | 1.0498 |  | MDP0000261625 |  | -1.1417 |  | MDP0000448896 |  |  | 1.0749 | MDP0000908305 |  |  | -4.2494 |
| MDP0000183682 | 3.2165 | 2.5218 | 1.6108 | MDP0000261713 | 1.1058 | 1.5264 | 1.5726 | MDP0000449345 |  | 1.7791 | 2.5946 | MDP0000908647 |  | 2.7455 |  |
| MDP0000183751 |  | -1.1474 |  | MDP0000261726 | 1.9054 |  |  | MDP0000450325 | -1.6852 |  |  | MDP0000908727 |  |  | -4.0908 |
| MDP0000184157 | 1.6474 |  |  | MDP0000261830 |  |  | 1.1639 | MDP0000453188 |  |  | 1.0588 | MDP0000909262 | -1.9226 | -2.0408 |  |
| MDP0000184324 |  |  | 1.2299 | MDP0000261839 | -1.2243 | -1.3888 |  | MDP0000454694 |  |  | 1.3692 | MDP0000909590 | 1.0175 |  |  |
| MDP0000184385 | 1.439 |  |  | MDP0000262388 |  |  | 1.0502 | MDP0000455180 |  | -1.5041 |  | MDP0000909752 |  |  | 1.062 |
| MDP0000184480 | 1.1732 |  |  | MDP0000262661 | 1.8722 |  |  | MDP0000456717 | 1.2996 |  |  | MDP0000910523 | -1.004 |  |  |
| MDP0000184534 | 1.5238 |  |  | MDP0000262753 |  | 1.4938 |  | MDP0000457380 | 1.0215 |  |  | MDP0000911918 |  | 1.0924 | 1.3259 |
| MDP0000184562 | -1.8483 |  |  | MDP0000263022 |  |  | 2.5384 | MDP0000458350 | 1.1148 |  |  | MDP0000912311 | -1.3692 |  |  |
| MDP0000184825 | 1.4357 |  |  | MDP0000263180 |  |  | 1.5789 | MDP0000459561 | -1.0755 |  |  | MDP0000914155 |  |  | 1.0576 |
| MDP0000184848 | 1.7967 |  |  | MDP0000263529 | -2.3886 | -2.1458 |  | MDP0000460051 |  | 1.4025 |  | MDP0000915138 |  | 1.5356 | 2.1635 |
| MDP0000185406 | 1.244 |  | 1.2827 | MDP0000263680 | -2.0406 |  |  | MDP0000460074 |  | 3.0507 |  | MDP0000915330 | 1.288 |  |  |
| MDP0000186135 | -2.9251 |  |  | MDP0000263736 | 1.6137 |  |  | MDP0000460502 |  | -1.0024 |  | MDP0000915991 | 1.1914 |  |  |
| MDP0000186322 | 1.1972 |  |  | MDP0000263835 |  | 1.3506 |  | MDP0000463045 | -2.4276 |  |  | MDP0000916623 | 1.6234 |  |  |
| MDP0000186494 | -1.4794 |  |  | MDP0000264409 | 1.4608 | 1.0208 |  | MDP0000463117 |  | -1.011 |  | MDP0000916647 |  |  | 1.1922 |
| MDP0000186587 |  |  | 1.2568 | MDP0000264424 | -1.7122 |  |  | MDP0000463624 | 1.2328 |  |  | MDP0000916930 |  |  | -1.079 |
| MDP0000187482 | -1.4934 |  |  | MDP0000264731 |  |  | 1.3584 | MDP0000465335 |  |  | 2.2259 | MDP0000917496 | 3.4996 |  |  |
| MDP0000187617 | 1.3892 |  |  | MDP0000264936 |  | 1.2499 |  | MDP0000465844 |  | -1.9578 | -2.2069 | MDP0000917582 |  | 1.1299 |  |
| MDP0000187703 | 2.0759 |  | -1.7292 | MDP0000265094 | -1.2248 |  |  | MDP0000466825 |  |  | 1.4186 | MDP0000918133 | 1.0408 |  |  |
| MDP0000187851 | -3.9879 |  |  | MDP0000265157 |  | 1.0457 |  | MDP0000468391 |  | -2.3154 |  | MDP0000918738 |  |  | 1.4443 |
| MDP0000187900 | -1.7772 | -1.4842 |  | MDP0000265372 |  |  | -1.0231 | MDP0000469664 |  | 1.4894 |  | MDP0000919471 |  |  | -1.6572 |
| MDP0000187921 |  | -1.2351 |  | MDP0000265423 |  |  | 1.6887 | MDP0000470297 | 1.069 | 2.5946 |  | MDP0000920069 | -1.1951 |  |  |
| MDP0000188093 |  |  | 1.0902 | MDP0000265560 | 1.0974 | 1.2067 |  | MDP0000472943 |  |  | 1.4861 | MDP0000920189 |  |  | 1.369 |
| MDP0000188275 | -3.5499 |  |  | MDP0000265654 |  | 1.3227 | 1.0845 | MDP0000475144 |  | -1.9707 | -2.6494 | MDP0000920266 | -1.5944 |  |  |
| MDP0000188310 |  | -1.3447 | -1.477 | MDP0000265806 | -1.0667 |  |  | MDP0000475658 |  | -1.6484 |  | MDP0000920792 | 2.753 |  |  |
| MDP0000188909 |  | -2.7534 |  | MDP0000265817 |  |  | -1.1224 | MDP0000476095 |  | -1.0727 |  | MDP0000920996 | 2.5992 |  |  |
| MDP0000189281 |  |  | 2.1348 | MDP0000265875 | 1.0644 |  |  | MDP0000477900 | -1.6694 |  |  | MDP0000921871 | -3.541 |  |  |
| MDP0000189486 |  |  | -1.4066 | MDP0000266004 |  | 1.3965 |  | MDP0000479094 | -2.0888 |  |  | MDP0000923711 |  |  | -1.1234 |
| MDP0000189901 |  | -1.852 |  | MDP0000266107 |  | -1.5587 |  | MDP0000479177 |  |  | -2.3778 | MDP0000924327 |  | 1.7822 |  |
| MDP0000190029 | -1.6662 |  |  | MDP0000266156 | -1.5818 |  |  | MDP0000480293 |  |  | 2.7068 | MDP0000925058 | -2.8797 |  |  |
| MDP0000190452 | -1.4054 |  |  | MDP0000267047 | -3.1442 |  |  | MDP0000480581 | -3.8837 |  |  | MDP0000928620 |  | -1.1907 |  |
| MDP0000190460 | 1.12 |  |  | MDP0000267128 |  | 1.3786 |  | MDP0000480605 | 1.1829 | 1.537 |  | MDP0000928643 |  |  | -1.2273 |
| MDP0000190785 | 1.1341 |  |  | MDP0000267178 |  | 1.6551 |  | MDP0000482092 |  |  | -1.2676 | MDP0000929213 | 1.8698 | 1.8087 |  |
| MDP0000190809 | -1.7551 |  |  | MDP0000267249 |  |  | 4.0528 | MDP0000482268 |  | 1.815 |  | MDP0000930268 | 1.1271 |  |  |
| MDP0000191472 |  | 1.5742 |  | MDP0000267662 | 1.5725 |  |  | MDP0000486046 |  | 1.4401 |  | MDP0000930655 | -3.4974 |  |  |
| MDP0000191620 |  |  | 1.1233 | MDP0000267894 | 1.1105 |  |  | MDP0000487271 |  |  | 1.3284 | MDP0000931334 |  |  | 1.1333 |
| MDP0000191851 | -2.4117 | -1.9169 |  | MDP0000268423 |  |  | -1.4433 | MDP0000487488 |  |  | -1.3402 | MDP0000932449 |  | 1.4005 |  |
| MDP0000191921 | -1.1864 |  |  | MDP0000268505 |  |  | -1.4963 | MDP0000487946 | -1.3902 |  |  | MDP0000932804 | -2.583 |  |  |
| MDP0000191939 | 1.3693 |  | 1.1078 | MDP0000268589 |  | 1.3801 |  | MDP0000491872 |  | -1.0239 |  | MDP0000933711 |  |  | 1.2977 |
| MDP0000192000 | 1.0924 |  |  | MDP0000268890 | 1.3427 |  |  | MDP0000494976 | 2.5743 | 1.3714 | 1.2547 | MDP0000934489 |  |  | 1.0824 |
| MDP0000192210 |  |  | -1.5381 | MDP0000268980 | 1.822 | 1.8662 |  | MDP0000495801 | -2.2145 |  |  | MDP0000935120 | 1.2207 |  |  |
| MDP0000192374 |  | 1.414 | 1.082 | MDP0000269628 | 1.2342 |  |  | MDP0000496370 |  |  | 1.0235 | MDP0000935925 | 1.3975 |  |  |
| MDP0000192586 |  |  | -1.4857 | MDP0000269735 |  | -1.8353 |  | MDP0000498615 | -2.636 |  |  | MDP0000936591 |  |  | 1.7724 |
| MDP0000192960 |  | 1.8204 |  | MDP0000270189 | 1.5276 |  |  | MDP0000499282 | -2.3776 | -2.6286 |  | MDP0000937996 |  | 2.9467 |  |
| MDP0000193181 |  | 1.285 |  | MDP0000270365 | -2.3191 |  |  | MDP0000501816 |  | 1.5739 |  | MDP0000939379 | 1.7674 |  | -1.8536 |
| MDP0000193206 |  |  | 1.2994 | MDP0000270618 | 1.0658 |  |  | MDP0000502306 |  |  | 1.8529 | MDP0000939502 |  | -1.2556 |  |
| MDP0000193325 | 1.0388 |  |  | MDP0000270938 |  | 1.403 |  | MDP0000505556 | 1.9485 |  |  | MDP0000940411 |  |  | 1.103 |
| MDP0000193385 |  | -2.0556 |  | MDP0000270966 |  |  | 1.1265 | MDP0000512219 | -7.08 |  |  | MDP0000940828 |  | -1.2369 |  |
| MDP0000193734 | 1.859 |  |  | MDP0000270977 |  |  | 1.3021 | MDP0000514153 | 1.6613 | 1.2437 |  | MDP0000943292 | -1.5178 |  |  |
| MDP0000193947 |  |  | 1.1381 | MDP0000271115 | -1.6787 |  |  | MDP0000516194 | 1.0789 |  |  | MDP0000943413 | 1.6719 |  |  |
| MDP0000194255 |  | -1.1269 |  | MDP0000271480 |  | -1.3856 |  | MDP0000516287 | -1.3932 |  |  | MDP0000943529 |  | 1.8479 |  |
| MDP0000194319 | -2.3466 |  |  | MDP0000271527 | -1.5747 |  |  | MDP0000517257 | 1.339 |  |  | MDP0000943790 | 1.0926 | 1.464 |  |
| MDP0000194613 |  |  | -1.0306 | MDP0000271554 |  |  | 1.1875 | MDP0000517262 | 1.0052 |  |  | MDP0000944210 |  | 1.0158 |  |
| MDP0000194772 |  |  | 1.0214 | MDP0000271872 |  |  | -2.1928 | MDP0000519318 | -2.581 |  |  | MDP0000944576 | -1.0886 |  |  |
| MDP0000195256 | 1.0679 |  |  | MDP0000272336 | 1.731 |  |  | MDP0000519389 | 1.1464 |  |  | MDP0000945035 |  | -1.6362 |  |
| MDP0000195390 | 1.5266 |  |  | MDP0000272499 | -2.8553 |  |  | MDP0000521662 | 1.9764 |  |  | MDP0000946489 |  |  | 1.1731 |
| MDP0000195397 | -2.5355 |  |  | MDP0000272549 | 1.3255 |  |  | MDP0000523477 |  |  | 1.4004 | MDP0000949258 |  | 1.7374 |  |
| MDP0000195801 | 1.129 |  |  | MDP0000272597 | -2.9016 |  |  | MDP0000523942 |  |  | 1.0572 | MDP0000950137 | 1.0858 |  |  |
| MDP0000195855 | -2.1906 |  |  | MDP0000272612 |  |  | 1.098 | MDP0000525641 |  | 1.3318 |  | MDP0000950422 |  |  | 1.0911 |
| MDP0000196399 |  | -1.0822 |  | MDP0000272674 |  | 1.4082 |  | MDP0000532061 | 1.1491 |  |  | MDP0000950554 | -2.516 |  |  |
| MDP0000196402 | 1.1533 | 1.1451 |  | MDP0000272843 |  |  | 1.538 | MDP0000532338 |  |  | 1.6287 | MDP0000951795 | 2.6377 |  |  |
| MDP0000196404 | 2.2895 | 1.7838 |  | MDP0000272901 |  |  | 1.2623 | MDP0000533075 | -2.4589 |  | -1.0355 | MDP0000951863 |  |  | -1.3128 |
| MDP0000196639 |  |  | -1.8409 | MDP0000273148 | 1.751 |  |  | MDP0000535805 |  |  | 1.0148 | MDP0000951897 |  |  | 1.0223 |

**Supplementary Table S7. GO Annotation of DEGs**

| Class | GO_Name | GO_ID | GO_Level | P_value | Enrichment Score | Hits Genes Counts In Selected Set | Hits Genes Counts In Background | All Genes Counts In Selected Set | All Genes Counts In Background | corrected p-value |
| --- | --- | --- | --- | --- | --- | --- | --- | --- | --- | --- |
| Molecular function | sequence-specific DNA binding | GO:0043565 | 6 | 0 | 2.587697 | 113 | 2544 | 1130 | 65831 | 0 |
|  | sequence-specific double-stranded DNA binding | GO:1990837 | 7 | 4.88E-15 | 2.571074 | 85 | 1926 | 1130 | 65831 | 2.48E-12 |
|  | double-stranded DNA binding | GO:0003690 | 6 | 1.73E-14 | 2.433029 | 90 | 2155 | 1130 | 65831 | 5.86E-12 |
|  | protein kinase activity | GO:0004672 | 6 | 7.72E-13 | 1.912204 | 133 | 4052 | 1130 | 65831 | 1.96E-10 |
|  | DNA-binding transcription factor activity | GO:0003700 | 3 | 1.40E-12 | 2.019263 | 114 | 3289 | 1130 | 65831 | 2.84E-10 |
|  | transcription regulator activity | GO:0140110 | 2 | 4.11E-12 | 1.943537 | 120 | 3597 | 1130 | 65831 | 6.95E-10 |
|  | transcription regulatory region sequence-specific DNA binding | GO:0000976 | 8 | 6.74E-12 | 2.408412 | 74 | 1790 | 1130 | 65831 | 9.77E-10 |
|  | regulatory region nucleic acid binding | GO:0001067 | 5 | 6.74E-12 | 2.408412 | 74 | 1790 | 1130 | 65831 | 9.77E-10 |
|  | protein serine/threonine kinase activity | GO:0004674 | 7 | 7.72E-12 | 1.905765 | 123 | 3760 | 1130 | 65831 | 8.71E-10 |
|  | kinase activity | GO:0016301 | 5 | 5.89E-11 | 1.757824 | 141 | 4673 | 1130 | 65831 | 5.98E-09 |
|  | phosphotransferase activity, alcohol group as acceptor | GO:0016773 | 5 | 1.13E-10 | 1.766966 | 135 | 4451 | 1130 | 65831 | 1.05E-08 |
|  | small molecule binding | GO:0036094 | 3 | 4.97E-09 | 1.318923 | 346 | 15283 | 1130 | 65831 | 4.20E-07 |
|  | microtubule binding | GO:0008017 | 6 | 7.01E-09 | 3.745126 | 27 | 420 | 1130 | 65831 | 5.48E-07 |
|  | anion binding | GO:0043168 | 4 | 2.13E-08 | 1.306222 | 340 | 15164 | 1130 | 65831 | 1.55E-06 |
|  | tubulin binding | GO:0015631 | 5 | 5.04E-08 | 3.40466 | 27 | 462 | 1130 | 65831 | 3.41E-06 |
|  | carbohydrate derivative binding | GO:0097367 | 3 | 8.62E-08 | 1.31072 | 311 | 13823 | 1130 | 65831 | 5.47E-06 |
|  | microtubule motor activity | GO:0003777 | 9 | 2.74E-07 | 5.170786 | 15 | 169 | 1130 | 65831 | 1.64E-05 |
|  | protein binding | GO:0005515 | 3 | 3.27E-07 | 1.42549 | 190 | 7765 | 1130 | 65831 | 1.85E-05 |
|  | nucleotide binding | GO:0000166 | 5 | 3.31E-07 | 1.284506 | 322 | 14604 | 1130 | 65831 | 1.77E-05 |
|  | nucleoside phosphate binding | GO:1901265 | 4 | 3.31E-07 | 1.284506 | 322 | 14604 | 1130 | 65831 | 1.77E-05 |
|  | flavonol synthase activity | GO:0045431 | 6 | 3.47E-07 | 29.12876 | 5 | 10 | 1130 | 65831 | 1.67E-05 |
|  | motor activity | GO:0003774 | 8 | 5.46E-07 | 4.343763 | 17 | 228 | 1130 | 65831 | 2.31E-05 |
|  | purine nucleotide binding | GO:0017076 | 6 | 9.69E-07 | 1.28547 | 300 | 13596 | 1130 | 65831 | 3.94E-05 |
|  | cytoskeletal protein binding | GO:0008092 | 4 | 1.26E-06 | 2.520045 | 34 | 786 | 1130 | 65831 | 4.93E-05 |
|  | hydrolase activity, acting on glycosyl bonds | GO:0016798 | 4 | 1.68E-06 | 1.865389 | 65 | 2030 | 1130 | 65831 | 6.30E-05 |
|  | DNA primase activity | GO:0003896 | 9 | 1.70E-06 | 22.40674 | 5 | 13 | 1130 | 65831 | 6.15E-05 |
|  | purine ribonucleotide binding | GO:0032555 | 7 | 2.51E-06 | 1.273646 | 297 | 13585 | 1130 | 65831 | 8.77E-05 |
|  | ribonucleotide binding | GO:0032553 | 6 | 2.67E-06 | 1.271274 | 299 | 13702 | 1130 | 65831 | 9.04E-05 |
|  | DNA binding | GO:0003677 | 5 | 2.71E-06 | 1.349669 | 213 | 9194 | 1130 | 65831 | 8.89E-05 |
|  | oxidoreductase activity | GO:0016491 | 3 | 3.49E-06 | 1.501056 | 127 | 4929 | 1130 | 65831 | 1.11E-04 |
|  | protein heterodimerization activity | GO:0046982 | 5 | 4.22E-06 | 3.426913 | 19 | 323 | 1130 | 65831 | 1.30E-04 |
|  | L-ascorbic acid binding | GO:0031418 | 6 | 5.71E-06 | 6.990903 | 9 | 75 | 1130 | 65831 | 1.70E-04 |
|  | adenyl ribonucleotide binding | GO:0032559 | 8 | 7.92E-06 | 1.267945 | 280 | 12865 | 1130 | 65831 | 2.30E-04 |
|  | adenyl nucleotide binding | GO:0030554 | 7 | 8.31E-06 | 1.267157 | 280 | 12873 | 1130 | 65831 | 2.34E-04 |
|  | polysaccharide binding | GO:0030247 | 4 | 9.92E-06 | 4.403185 | 13 | 172 | 1130 | 65831 | 2.72E-04 |
|  | organic acid binding | GO:0043177 | 4 | 1.33E-05 | 3.426913 | 17 | 289 | 1130 | 65831 | 3.54E-04 |
|  | carboxylic acid binding | GO:0031406 | 5 | 1.33E-05 | 3.426913 | 17 | 289 | 1130 | 65831 | 3.54E-04 |
|  | carbohydrate binding | GO:0030246 | 3 | 1.44E-05 | 2.02512 | 43 | 1237 | 1130 | 65831 | 3.66E-04 |
|  | naringenin 3-dioxygenase activity | GO:0045486 | 6 | 1.85E-05 | 14.56438 | 5 | 20 | 1130 | 65831 | 4.58E-04 |
|  | chalcone isomerase activity | GO:0045430 | 5 | 3.05E-05 | 13.24035 | 5 | 22 | 1130 | 65831 | 7.37E-04 |
|  | chitinase activity | GO:0004568 | 6 | 4.07E-05 | 6.29811 | 8 | 74 | 1130 | 65831 | 9.61E-04 |
|  | transferase activity, transferring glycosyl groups | GO:0016757 | 4 | 5.70E-05 | 1.784094 | 52 | 1698 | 1130 | 65831 | 0.001314 |
|  | dioxygenase activity | GO:0051213 | 4 | 7.88E-05 | 2.375919 | 25 | 613 | 1130 | 65831 | 0.001778 |
|  | calcium ion binding | GO:0005509 | 6 | 8.03E-05 | 2.326721 | 26 | 651 | 1130 | 65831 | 0.001773 |
|  | DNA replication origin binding | GO:0003688 | 8 | 8.39E-05 | 6.685289 | 7 | 61 | 1130 | 65831 | 0.001812 |
|  | drug binding | GO:0008144 | 3 | 9.08E-05 | 1.236246 | 265 | 12488 | 1130 | 65831 | 0.00192 |
|  | S-adenosylmethionine-dependent methyltransferase activity | GO:0008757 | 6 | 1.13E-04 | 2.543468 | 21 | 481 | 1130 | 65831 | 0.002344 |
|  | protein dimerization activity | GO:0046983 | 4 | 1.15E-04 | 1.609589 | 67 | 2425 | 1130 | 65831 | 0.002342 |
|  | oxidoreductase activity, acting on single donors with incorporation of molecular oxygen, incorporation of two atoms of oxygen | GO:0016702 | 5 | 1.22E-04 | 4.315372 | 10 | 135 | 1130 | 65831 | 0.002422 |
|  | active transmembrane transporter activity | GO:0022804 | 4 | 1.47E-04 | 1.716369 | 52 | 1765 | 1130 | 65831 | 0.002878 |
|  | purine ribonucleoside triphosphate binding | GO:0035639 | 5 | 1.50E-04 | 1.225031 | 268 | 12745 | 1130 | 65831 | 0.002877 |
|  | oxidoreductase activity, acting on CH or CH2 groups | GO:0016725 | 4 | 1.73E-04 | 9.396375 | 5 | 31 | 1130 | 65831 | 0.003196 |
|  | monosaccharide binding | GO:0048029 | 4 | 2.25E-04 | 4.406031 | 9 | 119 | 1130 | 65831 | 0.004078 |
|  | intramolecular lyase activity | GO:0016872 | 4 | 3.13E-04 | 8.322503 | 5 | 35 | 1130 | 65831 | 0.005289 |
|  | ATPase-coupled xenobiotic transmembrane transporter activity | GO:0008559 | 7 | 3.23E-04 | 6.473058 | 6 | 54 | 1130 | 65831 | 0.005374 |
|  | xyloglucan:xyloglucosyl transferase activity | GO:0016762 | 7 | 3.94E-04 | 6.241877 | 6 | 56 | 1130 | 65831 | 0.006457 |
|  | ATP binding | GO:0005524 | 9 | 4.09E-04 | 1.21602 | 251 | 12025 | 1130 | 65831 | 0.006586 |
|  | hydrolase activity, hydrolyzing O-glycosyl compounds | GO:0004553 | 5 | 4.26E-04 | 1.899702 | 33 | 1012 | 1130 | 65831 | 0.00675 |
|  | glucosyltransferase activity | GO:0046527 | 6 | 5.38E-04 | 2.16816 | 23 | 618 | 1130 | 65831 | 0.008402 |
|  | protein homodimerization activity | GO:0042803 | 5 | 5.90E-04 | 1.955403 | 29 | 864 | 1130 | 65831 | 0.009071 |
|  | transferase activity, transferring hexosyl groups | GO:0016758 | 5 | 6.20E-04 | 1.771029 | 38 | 1250 | 1130 | 65831 | 0.009385 |
|  | oxidoreductase activity, acting on single donors with incorporation of molecular oxygen | GO:0016701 | 4 | 7.34E-04 | 3.447191 | 10 | 169 | 1130 | 65831 | 0.010955 |
|  | 2-oxoglutarate-dependent dioxygenase activity | GO:0016706 | 5 | 7.68E-04 | 3.426913 | 10 | 170 | 1130 | 65831 | 0.011297 |
|  | methyltransferase activity | GO:0008168 | 5 | 8.35E-04 | 1.937305 | 28 | 842 | 1130 | 65831 | 0.012107 |
|  | hydrolase activity, hydrolyzing N-glycosyl compounds | GO:0016799 | 5 | 8.84E-04 | 1.838502 | 32 | 1014 | 1130 | 65831 | 0.012286 |
|  | nucleoside-triphosphatase activity | GO:0017111 | 7 | 0.001026 | 1.509388 | 62 | 2393 | 1130 | 65831 | 0.013887 |
|  | transferase activity, transferring one-carbon groups | GO:0016741 | 4 | 0.001177 | 1.868881 | 29 | 904 | 1130 | 65831 | 0.015722 |
|  | transmembrane transporter activity | GO:0022857 | 3 | 0.001372 | 1.412732 | 80 | 3299 | 1130 | 65831 | 0.01786 |
|  | pyrophosphatase activity | GO:0016462 | 6 | 0.001413 | 1.473439 | 65 | 2570 | 1130 | 65831 | 0.01815 |
|  | signaling receptor activity | GO:0038023 | 3 | 0.001553 | 1.812993 | 30 | 964 | 1130 | 65831 | 0.019459 |
|  | cis-regulatory region sequence-specific DNA binding | GO:0000987 | 9 | 0.001577 | 3.115376 | 10 | 187 | 1130 | 65831 | 0.019521 |
|  | xenobiotic transmembrane transporter activity | GO:0042910 | 4 | 0.001586 | 3.361011 | 9 | 156 | 1130 | 65831 | 0.01939 |
|  | hydrolase activity, acting on acid anhydrides, in phosphorus-containing anhydrides | GO:0016818 | 5 | 0.001683 | 1.462626 | 65 | 2589 | 1130 | 65831 | 0.020097 |
|  | transporter activity | GO:0005215 | 2 | 0.001877 | 1.386686 | 83 | 3487 | 1130 | 65831 | 0.022147 |
|  | transmembrane receptor protein kinase activity | GO:0019199 | 7 | 0.001887 | 2.132742 | 19 | 519 | 1130 | 65831 | 0.022016 |
|  | ubiquitin-like protein transferase activity | GO:0019787 | 4 | 0.002295 | 1.713457 | 33 | 1122 | 1130 | 65831 | 0.025877 |
|  | sucrose synthase activity | GO:0016157 | 8 | 0.00233 | 5.394215 | 5 | 54 | 1130 | 65831 | 0.025987 |
|  | hydrolase activity, acting on acid anhydrides | GO:0016817 | 4 | 0.002343 | 1.442018 | 65 | 2626 | 1130 | 65831 | 0.02585 |
|  | identical protein binding | GO:0042802 | 4 | 0.002357 | 1.607374 | 41 | 1486 | 1130 | 65831 | 0.025721 |
|  | ubiquitin-protein transferase activity | GO:0004842 | 5 | 0.002612 | 1.715033 | 32 | 1087 | 1130 | 65831 | 0.027616 |
|  | secondary active transmembrane transporter activity | GO:0015291 | 5 | 0.002612 | 1.715033 | 32 | 1087 | 1130 | 65831 | 0.027616 |
|  | protein disulfide oxidoreductase activity | GO:0015035 | 6 | 0.002631 | 3.401899 | 8 | 137 | 1130 | 65831 | 0.02725 |
|  | RNA polymerase II cis-regulatory region sequence-specific DNA binding | GO:0000978 | 10 | 0.002631 | 3.401899 | 8 | 137 | 1130 | 65831 | 0.02725 |
|  | molecular transducer activity | GO:0060089 | 2 | 0.0027 | 1.74598 | 30 | 1001 | 1130 | 65831 | 0.027408 |
|  | hormone binding | GO:0042562 | 3 | 0.002752 | 3.377248 | 8 | 138 | 1130 | 65831 | 0.027654 |
|  | disulfide oxidoreductase activity | GO:0015036 | 5 | 0.00307 | 3.048359 | 9 | 172 | 1130 | 65831 | 0.029395 |
|  | enzyme regulator activity | GO:0030234 | 3 | 0.003118 | 1.694764 | 32 | 1100 | 1130 | 65831 | 0.029579 |
|  | organic anion transmembrane transporter activity | GO:0008514 | 6 | 0.003124 | 2.263893 | 15 | 386 | 1130 | 65831 | 0.029362 |
|  | oxidoreductase activity, acting on paired donors, with incorporation or reduction of molecular oxygen | GO:0016705 | 4 | 0.003127 | 1.679038 | 33 | 1145 | 1130 | 65831 | 0.029117 |
|  | NAD+ nucleotidase, cyclic ADP-ribose generating | GO:0061809 | 7 | 0.003155 | 1.765379 | 28 | 924 | 1130 | 65831 | 0.029108 |
|  | NAD(P)+ nucleosidase activity | GO:0050135 | 6 | 0.003155 | 1.765379 | 28 | 924 | 1130 | 65831 | 0.029108 |
|  | NAD+ nucleosidase activity | GO:0003953 | 6 | 0.003155 | 1.765379 | 28 | 924 | 1130 | 65831 | 0.029108 |
|  | RNA polymerase II transcription regulatory region sequence-specific DNA binding | GO:0000977 | 9 | 0.003277 | 2.252224 | 15 | 388 | 1130 | 65831 | 0.029438 |
|  | proton-exporting ATPase activity, phosphorylative mechanism | GO:0008553 | 10 | 0.003973 | 4.775207 | 5 | 61 | 1130 | 65831 | 0.035067 |
|  | chitin binding | GO:0008061 | 4 | 0.003973 | 4.775207 | 5 | 61 | 1130 | 65831 | 0.035067 |
|  | ATPase-coupled transmembrane transporter activity | GO:0042626 | 6 | 0.004351 | 1.935466 | 20 | 602 | 1130 | 65831 | 0.037428 |
|  | oxidoreductase activity, acting on a sulfur group of donors | GO:0016667 | 4 | 0.005258 | 2.493513 | 11 | 257 | 1130 | 65831 | 0.044104 |
|  | enzyme inhibitor activity | GO:0004857 | 4 | 0.005657 | 2.062213 | 16 | 452 | 1130 | 65831 | 0.047068 |
|  | inorganic molecular entity transmembrane transporter activity | GO:0015318 | 4 | 0.006105 | 1.436116 | 53 | 2150 | 1130 | 65831 | 0.048789 |
| Cellular component | cell periphery | GO:0071944 | 3 | 1.58E-08 | 1.308467 | 344 | 11592 | 1181 | 52073 | 6.75E-06 |
|  | protein-DNA complex | GO:0032993 | 3 | 1.28E-06 | 3.416746 | 21 | 271 | 1181 | 52073 | 2.73E-04 |
|  | microtubule | GO:0005874 | 7 | 1.60E-06 | 2.44569 | 35 | 631 | 1181 | 52073 | 2.28E-04 |
|  | polymeric cytoskeletal fiber | GO:0099513 | 6 | 2.44E-06 | 2.365607 | 36 | 671 | 1181 | 52073 | 2.60E-04 |
|  | alpha DNA polymerase:primase complex | GO:0005658 | 9 | 2.45E-06 | 20.04195 | 5 | 11 | 1181 | 52073 | 2.10E-04 |
|  | supramolecular fiber | GO:0099512 | 5 | 2.95E-06 | 2.314064 | 37 | 705 | 1181 | 52073 | 2.10E-04 |
|  | supramolecular polymer | GO:0099081 | 4 | 2.95E-06 | 2.314064 | 37 | 705 | 1181 | 52073 | 2.10E-04 |
|  | kinesin complex | GO:0005871 | 4 | 8.12E-06 | 3.913517 | 15 | 169 | 1181 | 52073 | 4.34E-04 |
|  | intrinsic component of membrane | GO:0031224 | 3 | 8.73E-06 | 1.219718 | 361 | 13050 | 1181 | 52073 | 4.14E-04 |
|  | plasma membrane | GO:0005886 | 4 | 8.90E-06 | 1.260652 | 289 | 10108 | 1181 | 52073 | 3.80E-04 |
|  | nuclear replisome | GO:0043601 | 5 | 1.83E-05 | 6.916438 | 8 | 51 | 1181 | 52073 | 7.12E-04 |
|  | replisome | GO:0030894 | 4 | 2.12E-05 | 6.78343 | 8 | 52 | 1181 | 52073 | 7.56E-04 |
|  | membrane | GO:0016020 | 3 | 3.83E-05 | 1.149977 | 506 | 19401 | 1181 | 52073 | 0.001257 |
|  | integral component of membrane | GO:0016021 | 4 | 7.92E-05 | 1.198237 | 341 | 12548 | 1181 | 52073 | 0.002417 |
|  | microtubule associated complex | GO:0005875 | 3 | 9.31E-05 | 2.814402 | 18 | 282 | 1181 | 52073 | 0.002649 |
|  | DNA packaging complex | GO:0044815 | 3 | 9.87E-05 | 3.318775 | 14 | 186 | 1181 | 52073 | 0.002633 |
|  | supramolecular complex | GO:0099080 | 3 | 1.31E-04 | 1.84818 | 42 | 1002 | 1181 | 52073 | 0.003294 |
|  | microtubule cytoskeleton | GO:0015630 | 7 | 1.44E-04 | 1.872284 | 40 | 942 | 1181 | 52073 | 0.003412 |
|  | nuclear replication fork | GO:0043596 | 4 | 2.06E-04 | 4.968146 | 8 | 71 | 1181 | 52073 | 0.004619 |
|  | nucleosome | GO:0000786 | 4 | 2.48E-04 | 3.391715 | 12 | 156 | 1181 | 52073 | 0.005297 |
|  | cell wall | GO:0005618 | 4 | 4.13E-04 | 1.552204 | 64 | 1818 | 1181 | 52073 | 0.008407 |
|  | external encapsulating structure | GO:0030312 | 3 | 4.19E-04 | 1.551351 | 64 | 1819 | 1181 | 52073 | 0.00814 |
|  | plant-type vacuole membrane | GO:0009705 | 7 | 7.94E-04 | 2.42579 | 17 | 309 | 1181 | 52073 | 0.014735 |
|  | plant-type vacuole | GO:0000325 | 7 | 0.001934 | 1.995947 | 22 | 486 | 1181 | 52073 | 0.030582 |
|  | replication fork | GO:0005657 | 3 | 0.002116 | 2.979209 | 10 | 148 | 1181 | 52073 | 0.032272 |
|  | cytoskeleton | GO:0005856 | 6 | 0.002271 | 1.54484 | 48 | 1370 | 1181 | 52073 | 0.033442 |
|  | secretory vesicle | GO:0099503 | 8 | 0.003714 | 2.150844 | 16 | 328 | 1181 | 52073 | 0.049554 |
|  | DNA polymerase complex | GO:0042575 | 6 | 0.003776 | 4.792641 | 5 | 46 | 1181 | 52073 | 0.04886 |
| Biological process | response to abiotic stimulus | GO:0009628 | 3 | 0 | 1.927861 | 171 | 5111 | 1110 | 63960 | 0 |
|  | response to hormone | GO:0009725 | 5 | 0 | 2.053938 | 166 | 4657 | 1110 | 63960 | 0 |
|  | regulation of biological process | GO:0050789 | 3 | 0 | 1.688157 | 429 | 14643 | 1110 | 63960 | 0 |
|  | response to stimulus | GO:0050896 | 2 | 0 | 1.760801 | 523 | 17115 | 1110 | 63960 | 0 |
|  | response to organic substance | GO:0010033 | 4 | 0 | 1.893668 | 189 | 5751 | 1110 | 63960 | 0 |
|  | cellular response to stimulus | GO:0051716 | 3 | 0 | 1.798795 | 269 | 8617 | 1110 | 63960 | 0 |
|  | response to stress | GO:0006950 | 3 | 0 | 1.702526 | 335 | 11338 | 1110 | 63960 | 0 |
|  | biological regulation | GO:0065007 | 2 | 0 | 1.643712 | 466 | 16336 | 1110 | 63960 | 0 |
|  | response to endogenous stimulus | GO:0009719 | 3 | 1.11E-16 | 1.997613 | 168 | 4846 | 1110 | 63960 | 3.59E-14 |
|  | flavonoid metabolic process | GO:0009812 | 4 | 1.11E-16 | 6.05027 | 42 | 400 | 1110 | 63960 | 3.59E-14 |
|  | response to chemical | GO:0042221 | 3 | 2.22E-16 | 1.795575 | 242 | 7766 | 1110 | 63960 | 5.87E-14 |
|  | flavonoid biosynthetic process | GO:0009813 | 5 | 2.22E-16 | 6.599124 | 41 | 358 | 1110 | 63960 | 5.87E-14 |
|  | regulation of cellular process | GO:0050794 | 4 | 3.33E-16 | 1.695556 | 373 | 12676 | 1110 | 63960 | 7.46E-14 |
|  | anatomical structure development | GO:0048856 | 3 | 9.99E-16 | 1.634702 | 253 | 8918 | 1110 | 63960 | 2.08E-13 |
|  | developmental process | GO:0032502 | 2 | 1.67E-15 | 1.61946 | 260 | 9251 | 1110 | 63960 | 3.23E-13 |
|  | response to oxygen-containing compound | GO:1901700 | 4 | 2.66E-15 | 1.932356 | 155 | 4622 | 1110 | 63960 | 4.85E-13 |
|  | cell cycle | GO:0007049 | 3 | 3.44E-15 | 2.516351 | 89 | 2038 | 1110 | 63960 | 5.89E-13 |
|  | signal transduction | GO:0007165 | 5 | 1.03E-14 | 1.807697 | 177 | 5642 | 1110 | 63960 | 1.67E-12 |
|  | signaling | GO:0023052 | 2 | 1.25E-14 | 1.798465 | 178 | 5703 | 1110 | 63960 | 1.92E-12 |
|  | cellular response to chemical stimulus | GO:0070887 | 4 | 2.08E-14 | 1.927276 | 147 | 4395 | 1110 | 63960 | 3.02E-12 |
|  | defense response | GO:0006952 | 4 | 2.10E-14 | 1.757788 | 187 | 6130 | 1110 | 63960 | 2.91E-12 |
|  | multicellular organismal process | GO:0032501 | 2 | 3.50E-14 | 1.610333 | 239 | 8552 | 1110 | 63960 | 4.63E-12 |
|  | response to biotic stimulus | GO:0009607 | 3 | 4.87E-14 | 1.881118 | 152 | 4656 | 1110 | 63960 | 6.17E-12 |
|  | response to acid chemical | GO:0001101 | 4 | 7.23E-14 | 2.028359 | 125 | 3551 | 1110 | 63960 | 8.76E-12 |
|  | response to chitin | GO:0010200 | 6 | 8.14E-14 | 5.284823 | 31 | 338 | 1110 | 63960 | 9.47E-12 |
|  | hormone-mediated signaling pathway | GO:0009755 | 6 | 1.84E-13 | 2.163128 | 105 | 2797 | 1110 | 63960 | 2.06E-11 |
|  | cell communication | GO:0007154 | 3 | 1.96E-13 | 1.699701 | 192 | 6509 | 1110 | 63960 | 2.11E-11 |
|  | cellular response to hormone stimulus | GO:0032870 | 6 | 1.97E-13 | 2.106026 | 111 | 3037 | 1110 | 63960 | 2.05E-11 |
|  | multicellular organism development | GO:0007275 | 4 | 3.09E-13 | 1.634928 | 213 | 7507 | 1110 | 63960 | 3.10E-11 |
|  | response to external biotic stimulus | GO:0043207 | 4 | 5.45E-13 | 1.856697 | 145 | 4500 | 1110 | 63960 | 5.28E-11 |
|  | response to other organism | GO:0051707 | 5 | 5.45E-13 | 1.856697 | 145 | 4500 | 1110 | 63960 | 5.28E-11 |
|  | response to external stimulus | GO:0009605 | 3 | 6.16E-13 | 1.745491 | 171 | 5645 | 1110 | 63960 | 5.60E-11 |
|  | cellular response to endogenous stimulus | GO:0071495 | 4 | 1.90E-12 | 2.015865 | 113 | 3230 | 1110 | 63960 | 1.67E-10 |
|  | regulation of metabolic process | GO:0019222 | 4 | 3.56E-12 | 1.607503 | 207 | 7420 | 1110 | 63960 | 3.04E-10 |
|  | response to drug | GO:0042493 | 4 | 7.68E-12 | 2.577705 | 65 | 1453 | 1110 | 63960 | 6.38E-10 |
|  | biosynthetic process | GO:0009058 | 3 | 1.71E-11 | 1.392491 | 334 | 13821 | 1110 | 63960 | 1.38E-09 |
|  | organic substance biosynthetic process | GO:1901576 | 4 | 3.38E-11 | 1.392797 | 326 | 13487 | 1110 | 63960 | 2.66E-09 |
|  | regulation of cellular metabolic process | GO:0031323 | 5 | 3.75E-11 | 1.626921 | 182 | 6446 | 1110 | 63960 | 2.87E-09 |
|  | anthocyanin-containing compound biosynthetic process | GO:0009718 | 6 | 4.08E-11 | 7.515864 | 18 | 138 | 1110 | 63960 | 3.04E-09 |
|  | mitotic cell cycle | GO:0000278 | 4 | 4.82E-11 | 2.958115 | 48 | 935 | 1110 | 63960 | 3.51E-09 |
|  | regulation of biosynthetic process | GO:0009889 | 5 | 4.88E-11 | 1.724854 | 150 | 5011 | 1110 | 63960 | 3.46E-09 |
|  | regulation of cellular macromolecule biosynthetic process | GO:2000112 | 7 | 5.24E-11 | 1.755475 | 142 | 4661 | 1110 | 63960 | 3.63E-09 |
|  | regulation of cellular biosynthetic process | GO:0031326 | 6 | 5.32E-11 | 1.730519 | 148 | 4928 | 1110 | 63960 | 3.60E-09 |
|  | anthocyanin-containing compound metabolic process | GO:0046283 | 5 | 6.65E-11 | 6.402402 | 20 | 180 | 1110 | 63960 | 4.40E-09 |
|  | regulation of macromolecule biosynthetic process | GO:0010556 | 6 | 1.02E-10 | 1.73832 | 142 | 4707 | 1110 | 63960 | 6.62E-09 |
|  | cellular response to hypoxia | GO:0071456 | 7 | 2.10E-10 | 4.125993 | 29 | 405 | 1110 | 63960 | 1.33E-08 |
|  | cellular response to oxygen levels | GO:0071453 | 5 | 2.49E-10 | 4.095654 | 29 | 408 | 1110 | 63960 | 1.54E-08 |
|  | cellular response to decreased oxygen levels | GO:0036294 | 6 | 2.49E-10 | 4.095654 | 29 | 408 | 1110 | 63960 | 1.54E-08 |
|  | regulation of transcription, DNA-templated | GO:0006355 | 10 | 3.25E-10 | 1.779055 | 126 | 4081 | 1110 | 63960 | 1.93E-08 |
|  | regulation of nucleic acid-templated transcription | GO:1903506 | 9 | 3.67E-10 | 1.775575 | 126 | 4089 | 1110 | 63960 | 2.13E-08 |
|  | regulation of RNA biosynthetic process | GO:2001141 | 8 | 4.39E-10 | 1.770379 | 126 | 4101 | 1110 | 63960 | 2.50E-08 |
|  | cellular response to organic substance | GO:0071310 | 5 | 4.40E-10 | 1.834366 | 114 | 3581 | 1110 | 63960 | 2.46E-08 |
|  | regulation of primary metabolic process | GO:0080090 | 5 | 6.17E-10 | 1.606977 | 168 | 6024 | 1110 | 63960 | 3.39E-08 |
|  | secondary metabolic process | GO:0019748 | 3 | 6.47E-10 | 2.887644 | 44 | 878 | 1110 | 63960 | 3.49E-08 |
|  | response to hypoxia | GO:0001666 | 6 | 7.76E-10 | 3.690641 | 31 | 484 | 1110 | 63960 | 4.11E-08 |
|  | regulation of nitrogen compound metabolic process | GO:0051171 | 5 | 1.07E-09 | 1.611933 | 162 | 5791 | 1110 | 63960 | 5.55E-08 |
|  | response to decreased oxygen levels | GO:0036293 | 5 | 1.39E-09 | 3.601351 | 31 | 496 | 1110 | 63960 | 7.12E-08 |
|  | response to oxygen levels | GO:0070482 | 4 | 1.53E-09 | 3.586888 | 31 | 498 | 1110 | 63960 | 7.70E-08 |
|  | cell cycle process | GO:0022402 | 3 | 2.08E-09 | 2.399147 | 57 | 1369 | 1110 | 63960 | 1.03E-07 |
|  | RNA biosynthetic process | GO:0032774 | 8 | 2.58E-09 | 1.697421 | 131 | 4447 | 1110 | 63960 | 1.25E-07 |
|  | regulation of RNA metabolic process | GO:0051252 | 7 | 2.69E-09 | 1.696277 | 131 | 4450 | 1110 | 63960 | 1.28E-07 |
|  | proanthocyanidin biosynthetic process | GO:0010023 | 7 | 3.12E-09 | 20.0423 | 8 | 23 | 1110 | 63960 | 1.47E-07 |
|  | aromatic compound biosynthetic process | GO:0019438 | 5 | 3.44E-09 | 1.515523 | 191 | 7262 | 1110 | 63960 | 1.59E-07 |
|  | reproductive process | GO:0022414 | 2 | 3.46E-09 | 1.651016 | 141 | 4921 | 1110 | 63960 | 1.57E-07 |
|  | organic cyclic compound biosynthetic process | GO:1901362 | 5 | 3.61E-09 | 1.496665 | 200 | 7700 | 1110 | 63960 | 1.62E-07 |
|  | regulation of macromolecule metabolic process | GO:0060255 | 5 | 3.95E-09 | 1.54367 | 177 | 6607 | 1110 | 63960 | 1.74E-07 |
|  | cell division | GO:0051301 | 3 | 4.00E-09 | 2.399083 | 55 | 1321 | 1110 | 63960 | 1.74E-07 |
|  | regulation of nucleobase-containing compound metabolic process | GO:0019219 | 6 | 4.17E-09 | 1.668222 | 135 | 4663 | 1110 | 63960 | 1.78E-07 |
|  | reproduction | GO:0000003 | 2 | 4.41E-09 | 1.644666 | 141 | 4940 | 1110 | 63960 | 1.86E-07 |
|  | defense response to other organism | GO:0098542 | 6 | 5.53E-09 | 1.738007 | 117 | 3879 | 1110 | 63960 | 2.30E-07 |
|  | transcription, DNA-templated | GO:0006351 | 10 | 5.93E-09 | 1.682098 | 129 | 4419 | 1110 | 63960 | 2.43E-07 |
|  | cellular component organization | GO:0016043 | 4 | 6.57E-09 | 1.494774 | 195 | 7517 | 1110 | 63960 | 2.66E-07 |
|  | nucleic acid-templated transcription | GO:0097659 | 9 | 6.59E-09 | 1.679058 | 129 | 4427 | 1110 | 63960 | 2.63E-07 |
|  | mitotic cell cycle process | GO:1903047 | 4 | 9.93E-09 | 2.984717 | 36 | 695 | 1110 | 63960 | 3.91E-07 |
|  | interspecies interaction between organisms | GO:0044419 | 2 | 1.02E-08 | 1.573594 | 157 | 5749 | 1110 | 63960 | 3.94E-07 |
|  | positive regulation of anthocyanin biosynthetic process | GO:0031542 | 8 | 1.33E-08 | 17.07307 | 8 | 27 | 1110 | 63960 | 5.09E-07 |
|  | pigment biosynthetic process | GO:0046148 | 4 | 1.37E-08 | 3.431267 | 29 | 487 | 1110 | 63960 | 5.16E-07 |
|  | DNA replication | GO:0006260 | 7 | 1.45E-08 | 3.184615 | 32 | 579 | 1110 | 63960 | 5.42E-07 |
|  | cell wall organization | GO:0071555 | 6 | 1.87E-08 | 2.233993 | 58 | 1496 | 1110 | 63960 | 6.90E-07 |
|  | cellular component organization or biogenesis | GO:0071840 | 3 | 1.89E-08 | 1.4448 | 212 | 8455 | 1110 | 63960 | 6.86E-07 |
|  | cell wall organization or biogenesis | GO:0071554 | 3 | 1.89E-08 | 2.091359 | 67 | 1846 | 1110 | 63960 | 6.79E-07 |
|  | regulation of gene expression | GO:0010468 | 6 | 1.98E-08 | 1.577234 | 150 | 5480 | 1110 | 63960 | 7.03E-07 |
|  | regulation of anthocyanin biosynthetic process | GO:0031540 | 7 | 2.94E-08 | 12.64865 | 9 | 41 | 1110 | 63960 | 1.03E-06 |
|  | post-embryonic development | GO:0009791 | 3 | 3.19E-08 | 1.65711 | 123 | 4277 | 1110 | 63960 | 1.11E-06 |
|  | cellular response to stress | GO:0033554 | 4 | 3.46E-08 | 1.756022 | 102 | 3347 | 1110 | 63960 | 1.19E-06 |
|  | response to fungus | GO:0009620 | 6 | 6.86E-08 | 2.020224 | 67 | 1911 | 1110 | 63960 | 2.32E-06 |
|  | positive regulation of biological process | GO:0048518 | 4 | 7.58E-08 | 1.619524 | 126 | 4483 | 1110 | 63960 | 2.53E-06 |
|  | positive regulation of anthocyanin metabolic process | GO:0031539 | 7 | 7.58E-08 | 13.96888 | 8 | 33 | 1110 | 63960 | 2.51E-06 |
|  | defense response to fungus | GO:0050832 | 7 | 8.30E-08 | 2.090969 | 61 | 1681 | 1110 | 63960 | 2.71E-06 |
|  | system development | GO:0048731 | 4 | 8.37E-08 | 1.557633 | 144 | 5327 | 1110 | 63960 | 2.71E-06 |
|  | external encapsulating structure organization | GO:0045229 | 5 | 8.81E-08 | 2.134134 | 58 | 1566 | 1110 | 63960 | 2.82E-06 |
|  | regulation of flavonoid biosynthetic process | GO:0009962 | 6 | 9.63E-08 | 8.23166 | 11 | 77 | 1110 | 63960 | 3.05E-06 |
|  | pigment metabolic process | GO:0042440 | 3 | 1.05E-07 | 3.048763 | 30 | 567 | 1110 | 63960 | 3.28E-06 |
|  | response to abscisic acid | GO:0009737 | 6 | 1.05E-07 | 2.091529 | 60 | 1653 | 1110 | 63960 | 3.25E-06 |
|  | heterocycle biosynthetic process | GO:0018130 | 5 | 1.10E-07 | 1.469855 | 178 | 6978 | 1110 | 63960 | 3.36E-06 |
|  | microtubule-based process | GO:0007017 | 3 | 1.12E-07 | 2.912941 | 32 | 633 | 1110 | 63960 | 3.39E-06 |
|  | response to organonitrogen compound | GO:0010243 | 5 | 1.44E-07 | 2.597416 | 38 | 843 | 1110 | 63960 | 4.31E-06 |
|  | phenylpropanoid metabolic process | GO:0009698 | 5 | 1.51E-07 | 3.066105 | 29 | 545 | 1110 | 63960 | 4.49E-06 |
|  | cellular biosynthetic process | GO:0044249 | 4 | 1.75E-07 | 1.310482 | 298 | 13103 | 1110 | 63960 | 5.15E-06 |
|  | gametophyte development | GO:0048229 | 5 | 2.35E-07 | 2.412869 | 42 | 1003 | 1110 | 63960 | 6.83E-06 |
|  | response to alcohol | GO:0097305 | 5 | 2.53E-07 | 2.0373 | 60 | 1697 | 1110 | 63960 | 7.28E-06 |
|  | phenol-containing compound biosynthetic process | GO:0046189 | 6 | 2.71E-07 | 7.456916 | 11 | 85 | 1110 | 63960 | 7.73E-06 |
|  | DNA-dependent DNA replication | GO:0006261 | 8 | 3.45E-07 | 3.469365 | 23 | 382 | 1110 | 63960 | 9.75E-06 |
|  | syncytium formation | GO:0006949 | 4 | 4.01E-07 | 14.40541 | 7 | 28 | 1110 | 63960 | 1.12E-05 |
|  | macromolecule modification | GO:0043412 | 5 | 4.57E-07 | 1.443841 | 175 | 6984 | 1110 | 63960 | 1.27E-05 |
|  | positive regulation of flavonoid biosynthetic process | GO:0009963 | 7 | 5.04E-07 | 9.260618 | 9 | 56 | 1110 | 63960 | 1.38E-05 |
|  | leading strand elongation | GO:0006272 | 10 | 6.03E-07 | 18.1963 | 6 | 19 | 1110 | 63960 | 1.64E-05 |
|  | response to nitrogen compound | GO:1901698 | 4 | 6.66E-07 | 2.346064 | 41 | 1007 | 1110 | 63960 | 1.79E-05 |
|  | DNA replication initiation | GO:0006270 | 8 | 6.83E-07 | 6.815461 | 11 | 93 | 1110 | 63960 | 1.82E-05 |
|  | nuclear DNA replication | GO:0033260 | 10 | 7.98E-07 | 8.789739 | 9 | 59 | 1110 | 63960 | 2.11E-05 |
|  | response to salicylic acid | GO:0009751 | 6 | 8.93E-07 | 2.752625 | 30 | 628 | 1110 | 63960 | 2.34E-05 |
|  | cell cycle DNA replication initiation | GO:1902292 | 9 | 1.12E-06 | 24.00901 | 5 | 12 | 1110 | 63960 | 2.90E-05 |
|  | nuclear cell cycle DNA replication initiation | GO:1902315 | 10 | 1.12E-06 | 24.00901 | 5 | 12 | 1110 | 63960 | 2.90E-05 |
|  | mitotic DNA replication initiation | GO:1902975 | 11 | 1.12E-06 | 24.00901 | 5 | 12 | 1110 | 63960 | 2.90E-05 |
|  | microtubule-based movement | GO:0007018 | 4 | 1.14E-06 | 4.348802 | 16 | 212 | 1110 | 63960 | 2.89E-05 |
|  | developmental process involved in reproduction | GO:0003006 | 3 | 1.32E-06 | 1.593292 | 109 | 3942 | 1110 | 63960 | 3.31E-05 |
|  | regulation of anthocyanin metabolic process | GO:0031537 | 6 | 1.41E-06 | 8.23166 | 9 | 63 | 1110 | 63960 | 3.52E-05 |
|  | cellular protein modification process | GO:0006464 | 7 | 1.46E-06 | 1.476181 | 146 | 5699 | 1110 | 63960 | 3.60E-05 |
|  | protein modification process | GO:0036211 | 6 | 1.46E-06 | 1.476181 | 146 | 5699 | 1110 | 63960 | 3.60E-05 |
|  | nucleobase-containing compound biosynthetic process | GO:0034654 | 6 | 1.48E-06 | 1.449254 | 158 | 6282 | 1110 | 63960 | 3.58E-05 |
|  | reproductive system development | GO:0061458 | 5 | 1.79E-06 | 1.639987 | 96 | 3373 | 1110 | 63960 | 4.31E-05 |
|  | reproductive structure development | GO:0048608 | 4 | 1.79E-06 | 1.639987 | 96 | 3373 | 1110 | 63960 | 4.31E-05 |
|  | response to jasmonic acid | GO:0009753 | 6 | 2.11E-06 | 2.588797 | 31 | 690 | 1110 | 63960 | 4.99E-05 |
|  | phenylpropanoid biosynthetic process | GO:0009699 | 6 | 2.22E-06 | 3.111027 | 23 | 426 | 1110 | 63960 | 5.21E-05 |
|  | organonitrogen compound metabolic process | GO:1901564 | 4 | 2.58E-06 | 1.285806 | 279 | 12503 | 1110 | 63960 | 6.01E-05 |
|  | macromolecule biosynthetic process | GO:0009059 | 5 | 2.63E-06 | 1.363426 | 202 | 8537 | 1110 | 63960 | 6.08E-05 |
|  | regulation of response to stimulus | GO:0048583 | 4 | 3.09E-06 | 1.736417 | 76 | 2522 | 1110 | 63960 | 7.09E-05 |
|  | protein phosphorylation | GO:0006468 | 8 | 3.39E-06 | 1.787721 | 69 | 2224 | 1110 | 63960 | 7.72E-05 |
|  | response to radiation | GO:0009314 | 4 | 3.60E-06 | 1.897816 | 58 | 1761 | 1110 | 63960 | 8.11E-05 |
|  | secondary metabolite biosynthetic process | GO:0044550 | 4 | 3.67E-06 | 2.789874 | 26 | 537 | 1110 | 63960 | 8.22E-05 |
|  | response to antibiotic | GO:0046677 | 4 | 4.21E-06 | 2.315154 | 36 | 896 | 1110 | 63960 | 9.35E-05 |
|  | response to lipid | GO:0033993 | 5 | 4.45E-06 | 1.781842 | 68 | 2199 | 1110 | 63960 | 9.81E-05 |
|  | carbohydrate metabolic process | GO:0005975 | 4 | 6.20E-06 | 1.619596 | 90 | 3202 | 1110 | 63960 | 1.36E-04 |
|  | seed development | GO:0048316 | 5 | 6.51E-06 | 1.896582 | 55 | 1671 | 1110 | 63960 | 1.41E-04 |
|  | mitotic DNA replication | GO:1902969 | 11 | 8.12E-06 | 16.94754 | 5 | 17 | 1110 | 63960 | 1.75E-04 |
|  | tissue development | GO:0009888 | 4 | 9.41E-06 | 1.859833 | 56 | 1735 | 1110 | 63960 | 2.01E-04 |
|  | cellular macromolecule biosynthetic process | GO:0034645 | 6 | 1.01E-05 | 1.343884 | 195 | 8361 | 1110 | 63960 | 2.14E-04 |
|  | regulation of signaling | GO:0023051 | 4 | 1.03E-05 | 2.118442 | 40 | 1088 | 1110 | 63960 | 2.16E-04 |
|  | response to light stimulus | GO:0009416 | 5 | 1.07E-05 | 1.863133 | 55 | 1701 | 1110 | 63960 | 2.25E-04 |
|  | negative regulation of cellular process | GO:0048523 | 5 | 1.24E-05 | 1.734344 | 67 | 2226 | 1110 | 63960 | 2.57E-04 |
|  | DNA strand elongation involved in DNA replication | GO:0006271 | 9 | 1.34E-05 | 7.317031 | 8 | 63 | 1110 | 63960 | 2.76E-04 |
|  | fruit development | GO:0010154 | 5 | 1.37E-05 | 1.823672 | 57 | 1801 | 1110 | 63960 | 2.81E-04 |
|  | regulation of signal transduction | GO:0009966 | 6 | 1.38E-05 | 2.114058 | 39 | 1063 | 1110 | 63960 | 2.81E-04 |
|  | DNA strand elongation | GO:0022616 | 8 | 1.50E-05 | 7.202703 | 8 | 64 | 1110 | 63960 | 3.04E-04 |
|  | glucosamine-containing compound catabolic process | GO:1901072 | 7 | 1.59E-05 | 6.173745 | 9 | 84 | 1110 | 63960 | 3.19E-04 |
|  | amino sugar catabolic process | GO:0046348 | 6 | 1.59E-05 | 6.173745 | 9 | 84 | 1110 | 63960 | 3.19E-04 |
|  | chitin metabolic process | GO:0006030 | 7 | 1.59E-05 | 6.173745 | 9 | 84 | 1110 | 63960 | 3.19E-04 |
|  | chitin catabolic process | GO:0006032 | 8 | 1.59E-05 | 6.173745 | 9 | 84 | 1110 | 63960 | 3.19E-04 |
|  | regulation of cell communication | GO:0010646 | 5 | 1.62E-05 | 2.076455 | 40 | 1110 | 1110 | 63960 | 3.15E-04 |
|  | phenol-containing compound metabolic process | GO:0018958 | 5 | 1.64E-05 | 3.935135 | 14 | 205 | 1110 | 63960 | 3.18E-04 |
|  | positive regulation of macromolecule metabolic process | GO:0010604 | 6 | 1.89E-05 | 1.744432 | 63 | 2081 | 1110 | 63960 | 3.63E-04 |
|  | positive regulation of metabolic process | GO:0009893 | 5 | 1.91E-05 | 1.680138 | 71 | 2435 | 1110 | 63960 | 3.66E-04 |
|  | aminoglycan catabolic process | GO:0006026 | 6 | 2.11E-05 | 5.960857 | 9 | 87 | 1110 | 63960 | 4.02E-04 |
|  | abscisic acid-activated signaling pathway | GO:0009738 | 7 | 2.40E-05 | 2.287158 | 31 | 781 | 1110 | 63960 | 4.53E-04 |
|  | megagametogenesis | GO:0009561 | 3 | 2.70E-05 | 4.005781 | 13 | 187 | 1110 | 63960 | 5.07E-04 |
|  | negative regulation of biological process | GO:0048519 | 4 | 2.93E-05 | 1.540774 | 93 | 3478 | 1110 | 63960 | 5.46E-04 |
|  | RNA metabolic process | GO:0016070 | 7 | 2.93E-05 | 1.336111 | 182 | 7849 | 1110 | 63960 | 5.44E-04 |
|  | cellular response to acid chemical | GO:0071229 | 5 | 2.97E-05 | 1.817825 | 53 | 1680 | 1110 | 63960 | 5.47E-04 |
|  | positive regulation of cellular process | GO:0048522 | 5 | 3.16E-05 | 1.525559 | 96 | 3626 | 1110 | 63960 | 5.78E-04 |
|  | positive regulation of nitrogen compound metabolic process | GO:0051173 | 6 | 3.22E-05 | 1.748804 | 59 | 1944 | 1110 | 63960 | 5.85E-04 |
|  | phosphorelay signal transduction system | GO:0000160 | 7 | 3.59E-05 | 2.443984 | 26 | 613 | 1110 | 63960 | 6.49E-04 |
|  | regulation of auxin mediated signaling pathway | GO:0010928 | 7 | 3.88E-05 | 4.495304 | 11 | 141 | 1110 | 63960 | 6.97E-04 |
|  | glucosamine-containing compound metabolic process | GO:1901071 | 6 | 3.94E-05 | 5.516964 | 9 | 94 | 1110 | 63960 | 7.03E-04 |
|  | response to auxin | GO:0009733 | 6 | 3.99E-05 | 2.013659 | 39 | 1116 | 1110 | 63960 | 7.07E-04 |
|  | response to karrikin | GO:0080167 | 4 | 4.53E-05 | 3.402852 | 15 | 254 | 1110 | 63960 | 7.98E-04 |
|  | positive regulation of gene expression | GO:0010628 | 7 | 4.58E-05 | 1.823469 | 50 | 1580 | 1110 | 63960 | 8.03E-04 |
|  | positive regulation of cellular metabolic process | GO:0031325 | 6 | 4.89E-05 | 1.680084 | 64 | 2195 | 1110 | 63960 | 8.53E-04 |
|  | growth | GO:0040007 | 2 | 5.33E-05 | 1.717008 | 59 | 1980 | 1110 | 63960 | 9.23E-04 |
|  | positive regulation of RNA metabolic process | GO:0051254 | 8 | 5.56E-05 | 1.877605 | 45 | 1381 | 1110 | 63960 | 9.57E-04 |
|  | ethylene-activated signaling pathway | GO:0009873 | 8 | 5.84E-05 | 2.473916 | 24 | 559 | 1110 | 63960 | 1.00E-03 |
|  | maintenance of seed dormancy | GO:0010231 | 5 | 7.58E-05 | 11.08108 | 5 | 26 | 1110 | 63960 | 0.00129 |
|  | maintenance of dormancy | GO:0097437 | 4 | 7.58E-05 | 11.08108 | 5 | 26 | 1110 | 63960 | 0.00129 |
|  | positive regulation of transcription, DNA-templated | GO:0045893 | 11 | 7.58E-05 | 1.882773 | 43 | 1316 | 1110 | 63960 | 0.001276 |
|  | cellular response to ethylene stimulus | GO:0071369 | 7 | 7.67E-05 | 2.430437 | 24 | 569 | 1110 | 63960 | 0.001282 |
|  | cellular response to abscisic acid stimulus | GO:0071215 | 7 | 7.69E-05 | 2.146959 | 31 | 832 | 1110 | 63960 | 0.001279 |
|  | response to water | GO:0009415 | 5 | 7.73E-05 | 2.062248 | 34 | 950 | 1110 | 63960 | 0.001278 |
|  | cellular response to alcohol | GO:0097306 | 6 | 7.86E-05 | 2.144382 | 31 | 833 | 1110 | 63960 | 0.001292 |
|  | positive regulation of macromolecule biosynthetic process | GO:0010557 | 7 | 8.00E-05 | 1.820038 | 47 | 1488 | 1110 | 63960 | 0.001308 |
|  | photomorphogenesis | GO:0009640 | 7 | 8.08E-05 | 3.601351 | 13 | 208 | 1110 | 63960 | 0.001313 |
|  | positive regulation of nucleobase-containing compound metabolic process | GO:0045935 | 7 | 9.00E-05 | 1.824222 | 46 | 1453 | 1110 | 63960 | 0.001455 |
|  | positive regulation of nucleic acid-templated transcription | GO:1903508 | 10 | 9.57E-05 | 1.862955 | 43 | 1330 | 1110 | 63960 | 0.001538 |
|  | positive regulation of RNA biosynthetic process | GO:1902680 | 9 | 9.73E-05 | 1.861555 | 43 | 1331 | 1110 | 63960 | 0.001555 |
|  | stomatal movement | GO:0010118 | 3 | 1.07E-04 | 2.625131 | 20 | 439 | 1110 | 63960 | 0.001698 |
|  | response to inorganic substance | GO:0010035 | 4 | 1.07E-04 | 1.633932 | 64 | 2257 | 1110 | 63960 | 0.001698 |
|  | cellular water homeostasis | GO:0009992 | 8 | 1.10E-04 | 10.28958 | 5 | 28 | 1110 | 63960 | 0.001731 |
|  | cellular response to auxin stimulus | GO:0071365 | 7 | 1.16E-04 | 2.195109 | 28 | 735 | 1110 | 63960 | 0.001817 |
|  | response to water deprivation | GO:0009414 | 6 | 1.29E-04 | 2.031532 | 33 | 936 | 1110 | 63960 | 0.002007 |
|  | response to ethylene | GO:0009723 | 6 | 1.39E-04 | 2.10554 | 30 | 821 | 1110 | 63960 | 0.002159 |
|  | anatomical structure morphogenesis | GO:0009653 | 3 | 1.51E-04 | 1.537908 | 77 | 2885 | 1110 | 63960 | 0.002324 |
|  | response to UV-B | GO:0010224 | 7 | 1.59E-04 | 3.841441 | 11 | 165 | 1110 | 63960 | 0.002442 |
|  | cell cycle DNA replication | GO:0044786 | 9 | 1.68E-04 | 3.8183 | 11 | 166 | 1110 | 63960 | 0.002562 |
|  | cell volume homeostasis | GO:0006884 | 7 | 1.82E-04 | 9.29381 | 5 | 31 | 1110 | 63960 | 0.002761 |
|  | regulation of developmental growth | GO:0048638 | 5 | 1.85E-04 | 2.76714 | 17 | 354 | 1110 | 63960 | 0.002788 |
|  | response to organic cyclic compound | GO:0014070 | 5 | 1.87E-04 | 1.885979 | 38 | 1161 | 1110 | 63960 | 0.002807 |
|  | aminoglycan metabolic process | GO:0006022 | 5 | 1.88E-04 | 4.509518 | 9 | 115 | 1110 | 63960 | 0.002812 |
|  | plant organ development | GO:0099402 | 4 | 1.89E-04 | 1.552098 | 72 | 2673 | 1110 | 63960 | 0.002804 |
|  | positive regulation of cellular biosynthetic process | GO:0031328 | 7 | 1.90E-04 | 1.740615 | 48 | 1589 | 1110 | 63960 | 0.002802 |
|  | regulation of response to stress | GO:0080134 | 5 | 2.21E-04 | 1.777946 | 44 | 1426 | 1110 | 63960 | 0.003245 |
|  | positive regulation of biosynthetic process | GO:0009891 | 6 | 2.22E-04 | 1.717433 | 49 | 1644 | 1110 | 63960 | 0.003246 |
|  | movement of cell or subcellular component | GO:0006928 | 3 | 2.32E-04 | 2.713484 | 17 | 361 | 1110 | 63960 | 0.003382 |
|  | embryo sac development | GO:0009553 | 6 | 2.40E-04 | 2.705988 | 17 | 362 | 1110 | 63960 | 0.003475 |
|  | regulation of stomatal movement | GO:0010119 | 5 | 2.46E-04 | 3.055692 | 14 | 264 | 1110 | 63960 | 0.003545 |
|  | cellular response to jasmonic acid stimulus | GO:0071395 | 7 | 2.56E-04 | 2.69112 | 17 | 364 | 1110 | 63960 | 0.003668 |
|  | embryo development ending in seed dormancy | GO:0009793 | 6 | 2.65E-04 | 1.870175 | 37 | 1140 | 1110 | 63960 | 0.003782 |
|  | anatomical structure formation involved in morphogenesis | GO:0048646 | 3 | 2.74E-04 | 2.230514 | 24 | 620 | 1110 | 63960 | 0.003894 |
|  | cellular response to oxygen-containing compound | GO:1901701 | 5 | 2.84E-04 | 1.596239 | 61 | 2202 | 1110 | 63960 | 0.004006 |
|  | amino sugar metabolic process | GO:0006040 | 5 | 2.94E-04 | 4.250775 | 9 | 122 | 1110 | 63960 | 0.004126 |
|  | nuclear division | GO:0000280 | 7 | 2.99E-04 | 2.426174 | 20 | 475 | 1110 | 63960 | 0.004189 |
|  | double-strand break repair | GO:0006302 | 9 | 3.00E-04 | 2.752077 | 16 | 335 | 1110 | 63960 | 0.004175 |
|  | pollen development | GO:0009555 | 6 | 3.28E-04 | 2.125053 | 26 | 705 | 1110 | 63960 | 0.00452 |
|  | organelle organization | GO:0006996 | 5 | 3.30E-04 | 1.392012 | 109 | 4512 | 1110 | 63960 | 0.004524 |
|  | regulation of multicellular organismal process | GO:0051239 | 4 | 3.82E-04 | 1.666328 | 50 | 1729 | 1110 | 63960 | 0.005219 |
|  | karyogamy | GO:0000741 | 7 | 3.90E-04 | 5.238329 | 7 | 77 | 1110 | 63960 | 0.005297 |
|  | polar nucleus fusion | GO:0010197 | 8 | 3.90E-04 | 5.238329 | 7 | 77 | 1110 | 63960 | 0.005297 |
|  | phosphorylation | GO:0016310 | 6 | 3.96E-04 | 1.511938 | 72 | 2744 | 1110 | 63960 | 0.005339 |
|  | auxin-activated signaling pathway | GO:0009734 | 7 | 4.24E-04 | 2.124691 | 25 | 678 | 1110 | 63960 | 0.00568 |
|  | plant-type cell wall organization | GO:0009664 | 7 | 4.31E-04 | 2.571043 | 17 | 381 | 1110 | 63960 | 0.005729 |
|  | chromosome organization | GO:0051276 | 6 | 4.38E-04 | 1.665758 | 49 | 1695 | 1110 | 63960 | 0.005797 |
|  | mitotic nuclear division | GO:0140014 | 8 | 4.51E-04 | 3.020488 | 13 | 248 | 1110 | 63960 | 0.005938 |
|  | response to UV | GO:0009411 | 6 | 4.61E-04 | 2.870828 | 14 | 281 | 1110 | 63960 | 0.006037 |
|  | jasmonic acid mediated signaling pathway | GO:0009867 | 7 | 4.99E-04 | 2.626627 | 16 | 351 | 1110 | 63960 | 0.00651 |
|  | regulation of growth | GO:0040008 | 4 | 5.00E-04 | 1.902881 | 32 | 969 | 1110 | 63960 | 0.006497 |
|  | water homeostasis | GO:0030104 | 6 | 5.06E-04 | 5.960857 | 6 | 58 | 1110 | 63960 | 0.006542 |
|  | regulation of DNA replication | GO:0006275 | 8 | 5.24E-04 | 3.928747 | 9 | 132 | 1110 | 63960 | 0.006746 |
|  | regulation of microtubule cytoskeleton organization | GO:0070507 | 8 | 5.55E-04 | 5.859826 | 6 | 59 | 1110 | 63960 | 0.007113 |
|  | negative regulation of cellular macromolecule biosynthetic process | GO:2000113 | 8 | 5.89E-04 | 1.906372 | 31 | 937 | 1110 | 63960 | 0.007519 |
|  | negative regulation of transcription, DNA-templated | GO:0045892 | 11 | 5.94E-04 | 2.010057 | 27 | 774 | 1110 | 63960 | 0.007552 |
|  | cellular nitrogen compound biosynthetic process | GO:0044271 | 5 | 6.00E-04 | 1.255271 | 187 | 8584 | 1110 | 63960 | 0.007597 |
|  | cellular response to lipid | GO:0071396 | 6 | 6.12E-04 | 1.757031 | 39 | 1279 | 1110 | 63960 | 0.007706 |
|  | organelle fission | GO:0048285 | 6 | 6.36E-04 | 2.14103 | 23 | 619 | 1110 | 63960 | 0.007944 |
|  | meiotic cell cycle | GO:0051321 | 4 | 6.66E-04 | 2.334351 | 19 | 469 | 1110 | 63960 | 0.008278 |
|  | cellular developmental process | GO:0048869 | 3 | 6.68E-04 | 1.542308 | 61 | 2279 | 1110 | 63960 | 0.008266 |
|  | response to bacterium | GO:0009617 | 6 | 6.89E-04 | 1.631115 | 49 | 1731 | 1110 | 63960 | 0.008499 |
|  | negative regulation of macromolecule biosynthetic process | GO:0010558 | 7 | 7.36E-04 | 1.880284 | 31 | 950 | 1110 | 63960 | 0.009036 |
|  | negative regulation of RNA biosynthetic process | GO:1902679 | 9 | 7.46E-04 | 1.979369 | 27 | 786 | 1110 | 63960 | 0.009126 |
|  | negative regulation of nucleic acid-templated transcription | GO:1903507 | 10 | 7.46E-04 | 1.979369 | 27 | 786 | 1110 | 63960 | 0.009126 |
|  | negative regulation of cellular biosynthetic process | GO:0031327 | 7 | 7.49E-04 | 1.85689 | 32 | 993 | 1110 | 63960 | 0.009086 |
|  | gene expression | GO:0010467 | 5 | 7.59E-04 | 1.240326 | 197 | 9152 | 1110 | 63960 | 0.009159 |
|  | polysaccharide metabolic process | GO:0005976 | 5 | 7.70E-04 | 1.684385 | 43 | 1471 | 1110 | 63960 | 0.009253 |
|  | shoot system development | GO:0048367 | 5 | 7.89E-04 | 1.498117 | 67 | 2577 | 1110 | 63960 | 0.009445 |
|  | inflorescence development | GO:0010229 | 7 | 7.89E-04 | 5.487773 | 6 | 63 | 1110 | 63960 | 0.009411 |
|  | negative regulation of biosynthetic process | GO:0009890 | 6 | 8.13E-04 | 1.847587 | 32 | 998 | 1110 | 63960 | 0.00966 |
|  | mitotic spindle organization | GO:0007052 | 9 | 8.15E-04 | 4.636222 | 7 | 87 | 1110 | 63960 | 0.009645 |
|  | carbohydrate derivative catabolic process | GO:1901136 | 5 | 8.16E-04 | 3.169189 | 11 | 200 | 1110 | 63960 | 0.009614 |
|  | response to salt stress | GO:0009651 | 5 | 8.27E-04 | 1.728649 | 39 | 1300 | 1110 | 63960 | 0.009699 |
|  | regulation of biological quality | GO:0065008 | 3 | 8.30E-04 | 1.41088 | 88 | 3594 | 1110 | 63960 | 0.009697 |
|  | response to temperature stimulus | GO:0009266 | 4 | 9.01E-04 | 1.670755 | 43 | 1483 | 1110 | 63960 | 0.010363 |
|  | response to osmotic stress | GO:0006970 | 4 | 9.53E-04 | 1.654929 | 44 | 1532 | 1110 | 63960 | 0.010914 |
|  | methylation | GO:0032259 | 3 | 9.78E-04 | 1.807522 | 33 | 1052 | 1110 | 63960 | 0.011159 |
|  | phosphate ion transport | GO:0006817 | 8 | 9.97E-04 | 4.481682 | 7 | 90 | 1110 | 63960 | 0.011333 |
|  | macromolecule methylation | GO:0043414 | 6 | 0.001086 | 2.095332 | 22 | 605 | 1110 | 63960 | 0.012251 |
|  | protein metabolic process | GO:0019538 | 5 | 0.001098 | 1.236655 | 190 | 8853 | 1110 | 63960 | 0.012337 |
|  | developmental growth | GO:0048589 | 3 | 0.001106 | 1.714661 | 38 | 1277 | 1110 | 63960 | 0.012376 |
|  | negative regulation of response to stimulus | GO:0048585 | 5 | 0.001108 | 1.90036 | 28 | 849 | 1110 | 63960 | 0.01235 |
|  | cell growth | GO:0016049 | 3 | 0.001129 | 1.741711 | 36 | 1191 | 1110 | 63960 | 0.012541 |
|  | response to red or far red light | GO:0009639 | 6 | 0.001131 | 2.178511 | 20 | 529 | 1110 | 63960 | 0.012513 |
|  | transmembrane transport | GO:0055085 | 5 | 0.001132 | 1.620168 | 46 | 1636 | 1110 | 63960 | 0.012478 |
|  | DNA repair | GO:0006281 | 8 | 0.001146 | 1.828322 | 31 | 977 | 1110 | 63960 | 0.01259 |
|  | drug catabolic process | GO:0042737 | 5 | 0.001163 | 2.010057 | 24 | 688 | 1110 | 63960 | 0.012719 |
|  | negative regulation of nucleobase-containing compound metabolic process | GO:0045934 | 7 | 0.001171 | 1.869158 | 29 | 894 | 1110 | 63960 | 0.012767 |
|  | regulation of molecular function | GO:0065009 | 3 | 0.001184 | 1.824587 | 31 | 979 | 1110 | 63960 | 0.012852 |
|  | cytoskeleton-dependent cytokinesis | GO:0061640 | 5 | 0.001259 | 3.003971 | 11 | 211 | 1110 | 63960 | 0.013617 |
|  | root system development | GO:0022622 | 5 | 0.001339 | 1.696066 | 38 | 1291 | 1110 | 63960 | 0.014329 |
|  | root development | GO:0048364 | 5 | 0.001339 | 1.696066 | 38 | 1291 | 1110 | 63960 | 0.014329 |
|  | double-strand break repair via homologous recombination | GO:0000724 | 10 | 0.001357 | 2.975764 | 11 | 213 | 1110 | 63960 | 0.014415 |
|  | cell surface receptor signaling pathway | GO:0007166 | 6 | 0.001366 | 2.810811 | 12 | 246 | 1110 | 63960 | 0.01445 |
|  | drug metabolic process | GO:0017144 | 4 | 0.001446 | 1.582287 | 48 | 1748 | 1110 | 63960 | 0.015243 |
|  | spindle organization | GO:0007051 | 8 | 0.001515 | 3.13161 | 10 | 184 | 1110 | 63960 | 0.015915 |
|  | response to nematode | GO:0009624 | 6 | 0.001517 | 2.934434 | 11 | 216 | 1110 | 63960 | 0.015878 |
|  | regulation of cell size | GO:0008361 | 6 | 0.001545 | 4.158261 | 7 | 97 | 1110 | 63960 | 0.016113 |
|  | embryo sac central cell differentiation | GO:0009559 | 5 | 0.001545 | 4.158261 | 7 | 97 | 1110 | 63960 | 0.016113 |
|  | response to wounding | GO:0009611 | 4 | 0.001593 | 2.07201 | 21 | 584 | 1110 | 63960 | 0.0165 |
|  | cellular response to DNA damage stimulus | GO:0006974 | 5 | 0.001632 | 1.750933 | 33 | 1086 | 1110 | 63960 | 0.016839 |
|  | negative regulation of RNA metabolic process | GO:0051253 | 8 | 0.00166 | 1.872183 | 27 | 831 | 1110 | 63960 | 0.017007 |
|  | multidimensional cell growth | GO:0009825 | 4 | 0.001667 | 3.658516 | 8 | 126 | 1110 | 63960 | 0.017017 |
|  | cellular protein metabolic process | GO:0044267 | 6 | 0.001691 | 1.234355 | 179 | 8356 | 1110 | 63960 | 0.017201 |
|  | response to heat | GO:0009408 | 5 | 0.00173 | 2.018592 | 22 | 628 | 1110 | 63960 | 0.017544 |
|  | positive regulation of transcription from RNA polymerase II promoter in response to stress | GO:0036003 | 13 | 0.001893 | 5.649179 | 5 | 51 | 1110 | 63960 | 0.018866 |
|  | positive regulation of transcription from RNA polymerase II promoter in response to heat stress | GO:0061408 | 14 | 0.001893 | 5.649179 | 5 | 51 | 1110 | 63960 | 0.018866 |
|  | fluid transport | GO:0042044 | 5 | 0.001963 | 4.60973 | 6 | 75 | 1110 | 63960 | 0.019362 |
|  | water transport | GO:0006833 | 6 | 0.001963 | 4.60973 | 6 | 75 | 1110 | 63960 | 0.019362 |
|  | negative regulation of cellular metabolic process | GO:0031324 | 6 | 0.002006 | 1.669538 | 37 | 1277 | 1110 | 63960 | 0.019656 |
|  | embryo development | GO:0009790 | 5 | 0.002015 | 1.656295 | 38 | 1322 | 1110 | 63960 | 0.019678 |
|  | organic hydroxy compound biosynthetic process | GO:1901617 | 5 | 0.002151 | 2.061596 | 20 | 559 | 1110 | 63960 | 0.02073 |
|  | defense response to bacterium | GO:0042742 | 7 | 0.002166 | 1.614823 | 41 | 1463 | 1110 | 63960 | 0.020801 |
|  | anion transport | GO:0006820 | 6 | 0.002168 | 1.861071 | 26 | 805 | 1110 | 63960 | 0.020748 |
|  | recombinational repair | GO:0000725 | 9 | 0.002239 | 2.792237 | 11 | 227 | 1110 | 63960 | 0.021358 |
|  | microtubule cytoskeleton organization involved in mitosis | GO:1902850 | 8 | 0.002303 | 3.878378 | 7 | 104 | 1110 | 63960 | 0.021898 |
|  | cellular homeostasis | GO:0019725 | 5 | 0.002394 | 1.847302 | 26 | 811 | 1110 | 63960 | 0.02269 |
|  | cell wall macromolecule metabolic process | GO:0044036 | 6 | 0.002475 | 2.248649 | 16 | 410 | 1110 | 63960 | 0.023385 |
|  | mitotic cytokinesis | GO:0000281 | 6 | 0.002499 | 3.142998 | 9 | 165 | 1110 | 63960 | 0.023537 |
|  | glycosinolate metabolic process | GO:0019757 | 7 | 0.002567 | 3.414615 | 8 | 135 | 1110 | 63960 | 0.024101 |
|  | glucosinolate metabolic process | GO:0019760 | 8 | 0.002567 | 3.414615 | 8 | 135 | 1110 | 63960 | 0.024101 |
|  | S-glycoside metabolic process | GO:0016143 | 6 | 0.002567 | 3.414615 | 8 | 135 | 1110 | 63960 | 0.024101 |
|  | cellular response to heat | GO:0034605 | 6 | 0.002603 | 3.124064 | 9 | 166 | 1110 | 63960 | 0.024198 |
|  | regulation of cell cycle | GO:0051726 | 5 | 0.002977 | 1.868809 | 24 | 740 | 1110 | 63960 | 0.027154 |
|  | regulation of developmental process | GO:0050793 | 4 | 0.003037 | 1.448993 | 60 | 2386 | 1110 | 63960 | 0.027615 |
|  | cell differentiation | GO:0030154 | 4 | 0.003182 | 1.478882 | 54 | 2104 | 1110 | 63960 | 0.028847 |
|  | meristem development | GO:0048507 | 5 | 0.003183 | 1.858762 | 24 | 744 | 1110 | 63960 | 0.028769 |
|  | defense response to Gram-negative bacterium | GO:0050829 | 8 | 0.003275 | 4.165418 | 6 | 83 | 1110 | 63960 | 0.029506 |
|  | cytokinesis by cell plate formation | GO:0000911 | 5 | 0.003353 | 3.269312 | 8 | 141 | 1110 | 63960 | 0.030117 |
|  | amine metabolic process | GO:0009308 | 5 | 0.003409 | 2.408621 | 13 | 311 | 1110 | 63960 | 0.030519 |
|  | mitotic cell cycle phase transition | GO:0044772 | 5 | 0.003426 | 2.640991 | 11 | 240 | 1110 | 63960 | 0.030586 |
|  | homeostatic process | GO:0042592 | 4 | 0.003495 | 1.52684 | 46 | 1736 | 1110 | 63960 | 0.031105 |
|  | regulation of multicellular organismal development | GO:2000026 | 5 | 0.00357 | 1.559348 | 42 | 1552 | 1110 | 63960 | 0.031387 |
|  | regulation of jasmonic acid mediated signaling pathway | GO:2000022 | 7 | 0.003664 | 3.569481 | 7 | 113 | 1110 | 63960 | 0.032119 |
|  | positive regulation of response to stimulus | GO:0048584 | 5 | 0.004197 | 1.748072 | 27 | 890 | 1110 | 63960 | 0.036454 |
|  | microtubule cytoskeleton organization | GO:0000226 | 7 | 0.004472 | 2.114555 | 16 | 436 | 1110 | 63960 | 0.038614 |
|  | protein ubiquitination | GO:0016567 | 10 | 0.00463 | 1.554191 | 40 | 1483 | 1110 | 63960 | 0.039864 |
|  | regulation of meristem development | GO:0048509 | 5 | 0.00467 | 2.319136 | 13 | 323 | 1110 | 63960 | 0.040091 |
|  | phloem or xylem histogenesis | GO:0010087 | 5 | 0.004912 | 2.400901 | 12 | 288 | 1110 | 63960 | 0.041919 |
|  | regulation of catalytic activity | GO:0050790 | 4 | 0.004996 | 1.791346 | 24 | 772 | 1110 | 63960 | 0.04251 |
|  | organic hydroxy compound metabolic process | GO:1901615 | 4 | 0.005123 | 1.684503 | 29 | 992 | 1110 | 63960 | 0.043336 |
|  | regulation of microtubule-based process | GO:0032886 | 5 | 0.00515 | 3.799228 | 6 | 91 | 1110 | 63960 | 0.043442 |
|  | negative regulation of nitrogen compound metabolic process | GO:0051172 | 6 | 0.005239 | 1.621069 | 33 | 1173 | 1110 | 63960 | 0.04406 |
|  | cellular response to radiation | GO:0071478 | 6 | 0.005299 | 2.204106 | 14 | 366 | 1110 | 63960 | 0.044441 |
|  | cellular response to blue light | GO:0071483 | 8 | 0.005459 | 4.432432 | 5 | 65 | 1110 | 63960 | 0.045648 |
|  | meiotic cell cycle process | GO:1903046 | 4 | 0.005681 | 2.186186 | 14 | 369 | 1110 | 63960 | 0.04683 |
|  | chromatin organization involved in negative regulation of transcription | GO:0097549 | 7 | 0.005689 | 2.466295 | 11 | 257 | 1110 | 63960 | 0.046767 |
|  | regulation of meristem growth | GO:0010075 | 6 | 0.006073 | 3.252833 | 7 | 124 | 1110 | 63960 | 0.048283 |
|  | regulation of mitotic cell cycle | GO:0007346 | 6 | 0.006084 | 2.168556 | 14 | 372 | 1110 | 63960 | 0.048242 |
|  | positive regulation of growth | GO:0045927 | 5 | 0.006136 | 2.954955 | 8 | 156 | 1110 | 63960 | 0.048518 |
|  | negative regulation of cell cycle | GO:0045786 | 6 | 0.006335 | 2.560961 | 10 | 225 | 1110 | 63960 | 0.049827 |
|  | positive regulation of catalytic activity | GO:0043085 | 5 | 0.006346 | 2.092795 | 15 | 413 | 1110 | 63960 | 0.049778 |
|  | nucleus organization | GO:0006997 | 6 | 0.006369 | 2.936134 | 8 | 157 | 1110 | 63960 | 0.049824 |

**Supplementary Table S8. The DEGs significantly enriched in KEGG pathway after ALA treatment**

| Illumination Time (h) | ID | Pathway | DEGs |
| --- | --- | --- | --- |
| 24 | mdm00941 | Flavonoid biosynthesis | MDP0000686666\|MDP0000729533\|MDP0000686661\|MDP0000264424\|MDP0000759336\|MDP0000575740\|MDP0000183682\|MDP0000494976\|MDP0000140621\|MDP0000636927\|MDP0000294667\|MDP0000286933\|MDP0000225491\|MDP0000240641\|MDP0000688415\|MDP0000252589\|MDP0000134791\|MDP0000240643\|MDP0000788934\|MDP0000271527\|MDP0000361449 |
|  | mdm03430 | Mismatch repair | MDP0000184480\|MDP0000125070\|MDP0000568341\|MDP0000208491\|MDP0000248100\|MDP0000786815\|MDP0000245551\|MDP0000321215 |
|  | mdm03030 | DNA replication | MDP0000184480\|MDP0000125070\|MDP0000195390\|MDP0000208491\|MDP0000196740\|MDP0000786815\|MDP0000568341\|MDP0000321215\|MDP0000131617 |
|  | mdm04712 | Circadian rhythm - plant | MDP0000686666\|MDP0000686661\|MDP0000575740\|MDP0000252999\|MDP0000287992\|MDP0000241199 |
| 48 | mdm00941 | Flavonoid biosynthesis | MDP0000686666\|MDP0000286933\|MDP0000686661\|MDP0000494976\|MDP0000575740\|MDP0000183682\|MDP0000759336\|MDP0000170162\|MDP0000294667\|MDP0000360447\|MDP0000788934\|MDP0000688415\|MDP0000252589\|MDP0000240643\|MDP0000134791 |
| 72 | mdm00941 | Flavonoid biosynthesis | MDP0000686666\|MDP0000286933\|MDP0000686661\|MDP0000494976\|MDP0000575740\|MDP0000183682\|MDP0000205890\|MDP0000294667\|MDP0000523477\|MDP0000360447\|MDP0000788934\|MDP0000688415\|MDP0000252589\|MDP0000240643 |
|  | mdm01110 | Biosynthesis of secondary metabolites | MDP0000286933\|MDP0000307964\|MDP0000280527\|MDP0000332597\|MDP0000788934\|MDP0000271872\|MDP0000252589\|MDP0000523942\|MDP0000171795\|MDP0000183682\|MDP0000198843\|MDP0000494976\|MDP0000251253\|MDP0000303739\|MDP0000294667\|MDP0000293578\|MDP0000523477\|MDP0000360447\|MDP0000194772\|MDP0000248777\|MDP0000176374\|MDP0000240643\|MDP0000800945\|MDP0000272901\|MDP0000274409\|MDP0000238942\|MDP0000130459\|MDP0000223309\|MDP0000575740\|MDP0000397367\|MDP0000205890\|MDP0000874800\|MDP0000931334\|MDP0000680997\|MDP0000754054\|MDP0000280662\|MDP0000263180\|MDP0000686666\|MDP0000686661\|MDP0000547655\|MDP0000634676\|MDP0000442206\|MDP0000272843\|MDP0000688415\|MDP0000296410 |
|  | mdm01100 | Metabolic pathways | MDP0000286933\|MDP0000307964\|MDP0000297138\|MDP0000280527\|MDP0000270977\|MDP0000788934\|MDP0000271872\|MDP0000252589\|MDP0000523942\|MDP0000575740\|MDP0000194772\|MDP0000183682\|MDP0000126946\|MDP0000209143\|MDP0000198843\|MDP0000494976\|MDP0000251253\|MDP0000303739\|MDP0000332597\|MDP0000293578\|MDP0000156131\|MDP0000523477\|MDP0000360447\|MDP0000171795\|MDP0000248777\|MDP0000176374\|MDP0000252292\|MDP0000240643\|MDP0000800945\|MDP0000272901\|MDP0000274409\|MDP0000238942\|MDP0000233546\|MDP0000130459\|MDP0000223309\|MDP0000939379\|MDP0000787842\|MDP0000397367\|MDP0000205890\|MDP0000157447\|MDP0000874800\|MDP0000931334\|MDP0000680997\|MDP0000448896\|MDP0000754054\|MDP0000280662\|MDP0000263180\|MDP0000686885\|MDP0000686666\|MDP0000686661\|MDP0000127757\|MDP0000547655\|MDP0000634676\|MDP0000239530\|MDP0000442206\|MDP0000272843\|MDP0000210077\|MDP0000294667\|MDP0000296410\|MDP0000146639 |
|  | mdm00592 | alpha-Linolenic acid metabolism | MDP0000296410\|MDP0000874800\|MDP0000272843\|MDP0000680997\|MDP0000523942\|MDP0000272901 |
|  | mdm03030 | DNA replication | MDP0000125070\|MDP0000144558\|MDP0000313603\|MDP0000770205\|MDP0000210654\|MDP0000231390 |
|  | mdm04712 | Circadian rhythm - plant | MDP0000139278\|MDP0000234215\|MDP0000686666\|MDP0000575740\|MDP0000686661 |
|  | mdm00944 | Flavone and flavonol biosynthesis | MDP0000286933\|MDP0000319451 |
|  | mdm00350 | Tyrosine metabolism | MDP0000296410\|MDP0000680997\|MDP0000523942\|MDP0000272901 |
|  | mdm00010 | Glycolysis / Gluconeogenesis | MDP0000238942\|MDP0000198843\|MDP0000296410\|MDP0000680997\|MDP0000523942\|MDP0000800945\|MDP0000272901 |
|  | mdm03430 | Mismatch repair | MDP0000313603\|MDP0000210654\|MDP0000248100\|MDP0000125070 |
|  | mdm00071 | Fatty acid degradation | MDP0000296410\|MDP0000680997\|MDP0000523942\|MDP0000272901 |

**Supplementary Table S9. Differentially expressed transcription factors related to anthocyanin metabolism in apple calli**

| Type of TFs | Number of DEGs | Description |
| --- | --- | --- |
| AP2-EREBP | 18 | Ethylene-responsive transcription factor |
| ARF | 2 | Auxin response factor |
| bHLH | 12 | Basic helix-loop-helix protein |
| bZIP | 2 | Basic Leucine Zipper Domain transcription factor |
| C2C2-Dof | 3 | Zinc finger protein |
| C2H2 | 7 | Zinc finger C2H2 domain-containing protein |
| C3H | 3 | Zinc finger CCCH domain-containing protein |
| CCAAT | 7 | Zinc finger CCCH domain-containing protein |
| GRAS | 3 | Scarecrow-like protein |
| HB | 6 | Homeobox-leucine zipper protein |
| HSF | 5 | Heat stress transcription factor |
| LOB | 4 | LOB domain-containing protei |
| MADS | 5 | MADS-box protein |
| MYB | 27 | Myb (Myeloblastosis) -related protein |
| NAC | 7 | NAC domain-containing protein |
| Orphans | 8 | Orphan Transcription Factor |
| TIFY | 2 | TIFY protein |
| Trihelix | 3 | Trihelix transcription factor |
| WRKY | 13 | WRKY transcription factor |
| Other TFs | 34 |  |
| Total TFs | 171 |  |

**Supplementary Table S10. Correlations between the relative expressions of MdMYB10 and MdMYB9 and anthocyanin metabolism genes under ALA treatment**

|  | Gene | *MdCHS* | *MdF3’H* | *MdDFR* | *MdANS* | *MdUFGT* | *MdGST* | *MdMATE* |
| --- | --- | --- | --- | --- | --- | --- | --- | --- |
| Correlations | *MdMYB10* | 0.96** | 0.97** | 0.99** | 0.98** | 0.98** | 0.88** | 0.92** |
|  | *MdMYB9* | 0.99** | 0.98** | 0.96** | 0.99** | 0.98** | 0.85** | 0.94** |
| Note: Pearson correlation test, ** represent correlation between MYB genes of apple and anthocyanin biosynthesis and transport genes is significant at the  *P* = 0.01 level. | | | | | | | | |


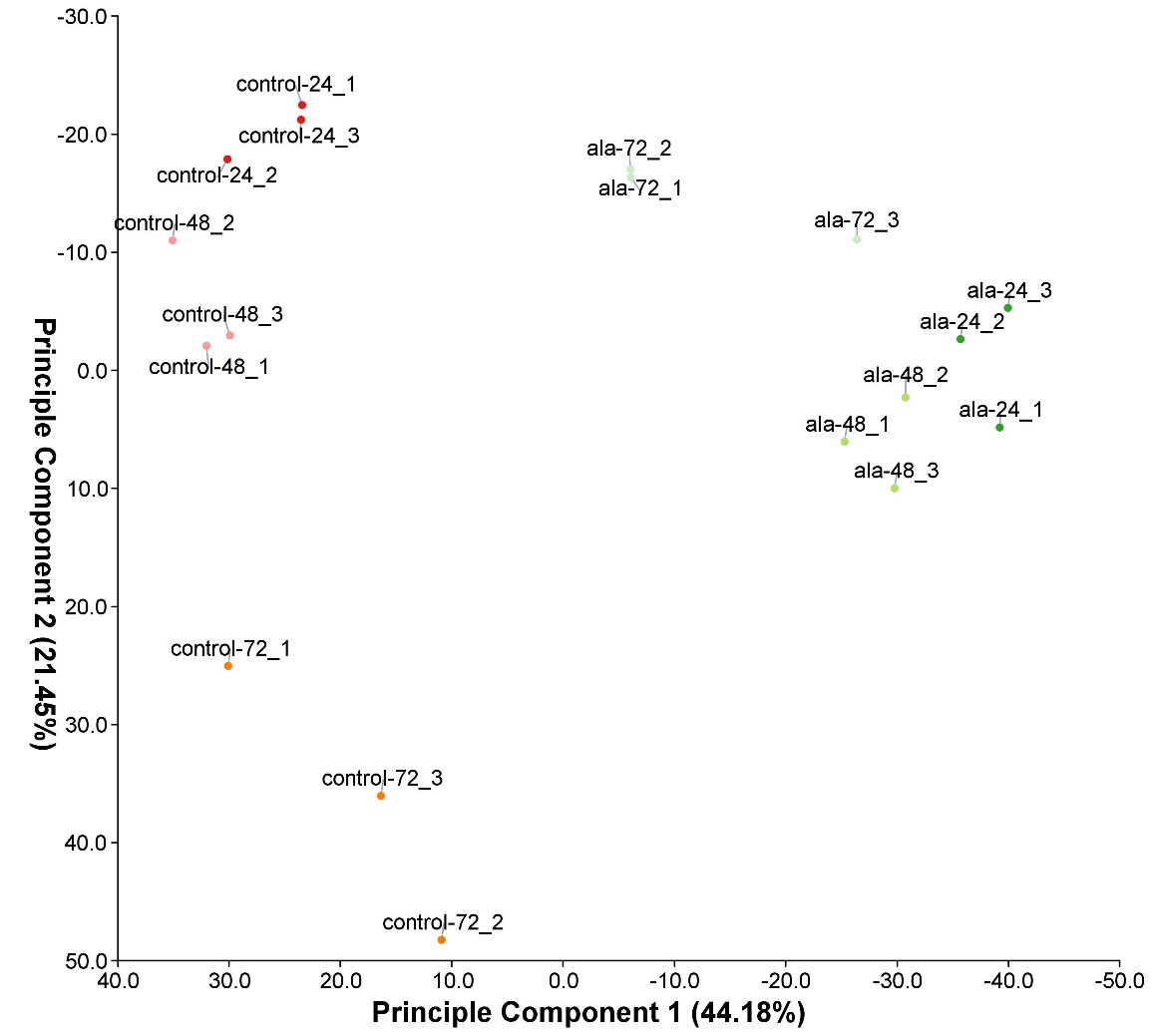


**Supplementary Figure 1.** Principal component analysis (PCA) showing the divergence of the respective transcriptomes in response to ALA treatment. The PCA was conducted with the using the internal steps of the *R* package version 3.5.3 (http://www.Bioconductor.org).


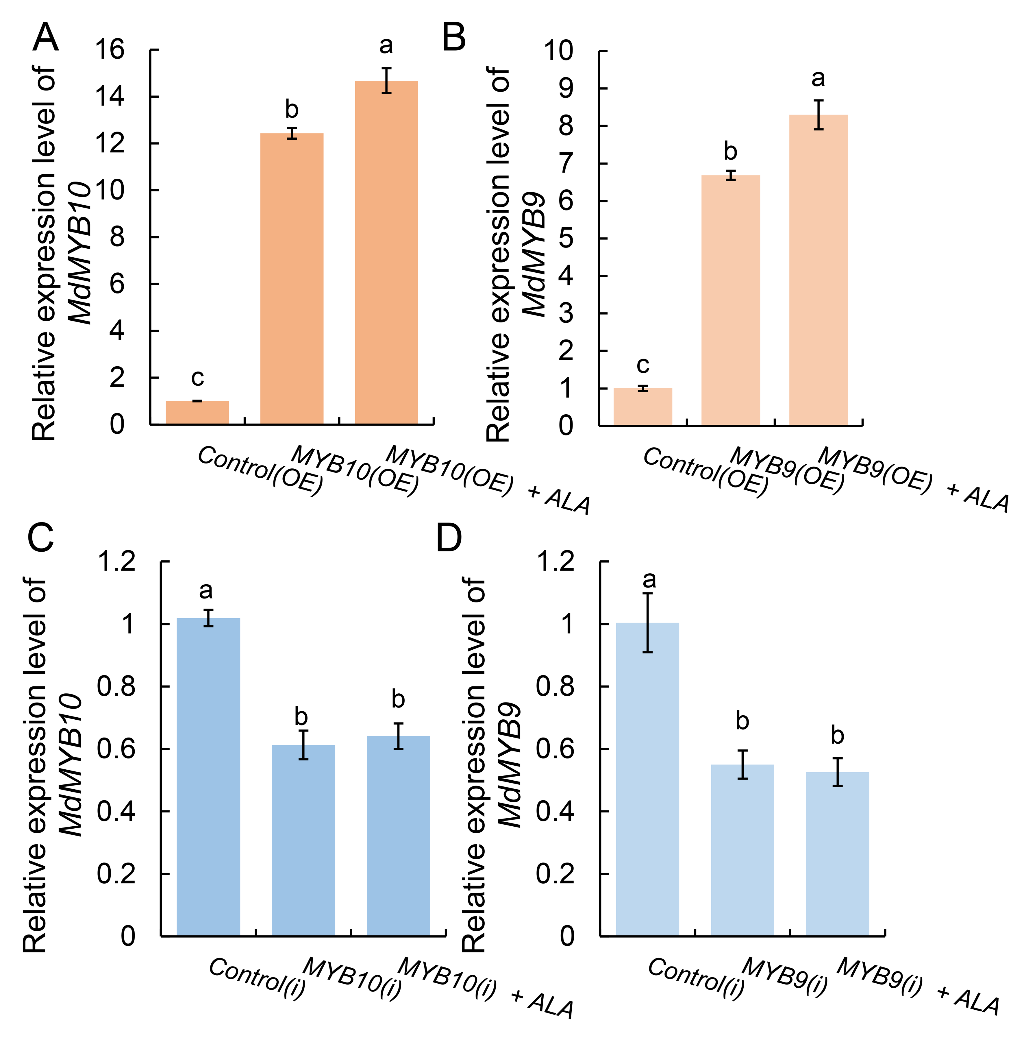


**Supplementary Figure 2.** qRT-PCR analysis of the relative expression of target genes in transgenic calli. Relative expression level was calculated using the 2^-ΔΔCT^ method with three replicates. The different letters in each gene represent significant differences (*P* < 0.05).


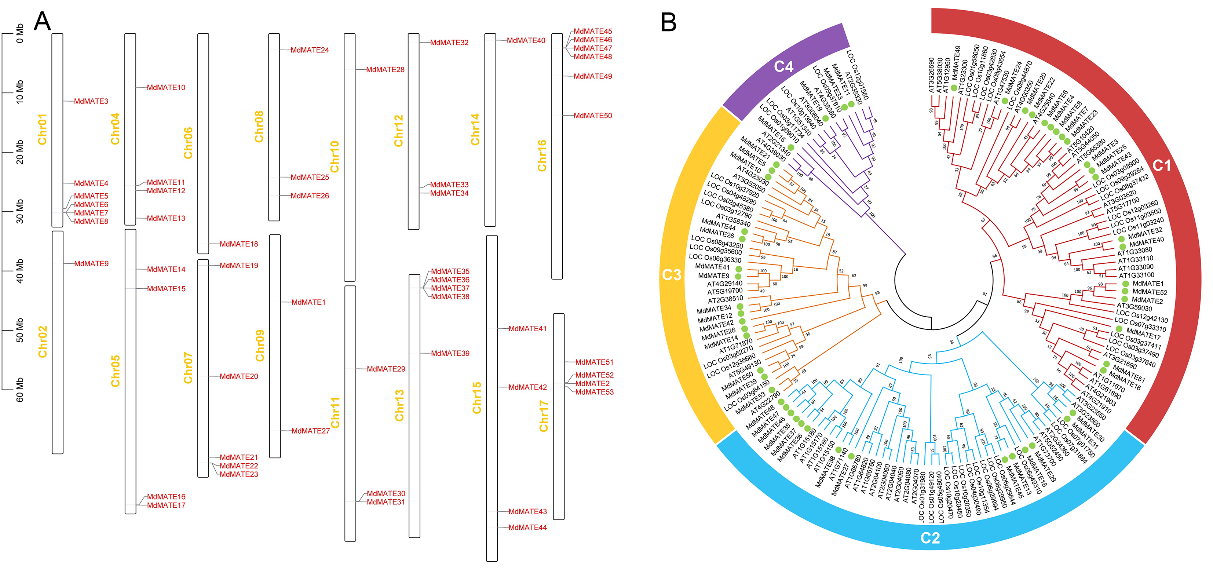


**Supplementary Figure 3.** Chromosomal location of MdMATEs and phylogenetic analysis of MdMATEs proteins and MATE proteins from other species. The phylogenetic tree was generated with MEGA X software using the maximum likelihood (ML) method. Bootstrap values from 1000 replicates are indicated at each branch.


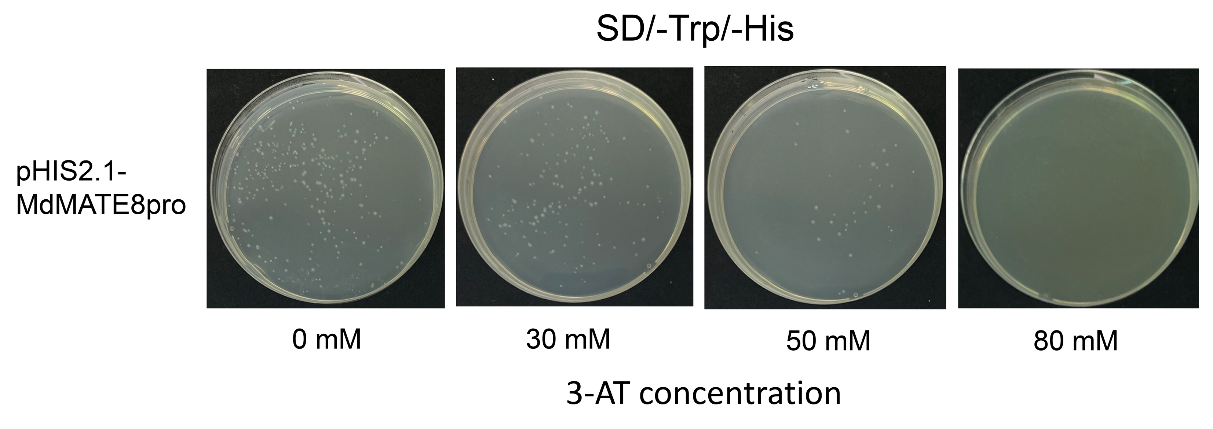


**Supplementary Figure 4.** Screening of the optimal concentration of 3-AT used for yeast one hybrid assay. The yeast strain Y187 transformed with the pHIS2-MdMATE8pro vectors were grown on -Trp/-His medium containing different concentrations of 3-AT.
